# Towards precision medicine for pain: diagnostic biomarkers and repurposed drugs

**DOI:** 10.1038/s41380-018-0345-5

**Published:** 2019-02-12

**Authors:** A. B. Niculescu, H. Le-Niculescu, D. F. Levey, K. Roseberry, K. C. Soe, J. Rogers, F. Khan, T. Jones, S. Judd, M. A. McCormick, A. R. Wessel, A. Williams, S. M. Kurian, F. A. White

**Affiliations:** 1 Department of Psychiatry, Indiana University School of Medicine, Indianapolis, IN USA; 2 Stark Neuroscience Research Institute, Indiana University School of Medicine, Indianapolis, IN USA; 3 Indianapolis VA Medical Center, Indianapolis, IN USA; 4 Department of Molecular and Experimental Medicine, The Scripps Research Institute, La Jolla, CA USA; 5 Department of Anesthesia, Indiana University School of Medicine, Indianapolis, IN USA

**Keywords:** Diagnostic markers, Genetics

## Abstract

We endeavored to identify objective blood biomarkers for pain, a subjective sensation with a biological basis, using a stepwise discovery, prioritization, validation, and testing in independent cohorts design. We studied psychiatric patients, a high risk group for co-morbid pain disorders and increased perception of pain. For discovery, we used a powerful within-subject longitudinal design. We were successful in identifying blood gene expression biomarkers that were predictive of pain state, and of future emergency department (ED) visits for pain, more so when personalized by gender and diagnosis. MFAP3, which had no prior evidence in the literature for involvement in pain, had the most robust empirical evidence from our discovery and validation steps, and was a strong predictor for pain in the independent cohorts, particularly in females and males with PTSD. Other biomarkers with best overall convergent functional evidence for involvement in pain were GNG7, CNTN1, LY9, CCDC144B, and GBP1. Some of the individual biomarkers identified are targets of existing drugs. Moreover, the biomarker gene expression signatures were used for bioinformatic drug repurposing analyses, yielding leads for possible new drug candidates such as SC-560 (an NSAID), and amoxapine (an antidepressant), as well as natural compounds such as pyridoxine (vitamin B6), cyanocobalamin (vitamin B12), and apigenin (a plant flavonoid). Our work may help mitigate the diagnostic and treatment dilemmas that have contributed to the current opioid epidemic.

## Introduction

“ The greatest evil is physical pain”

– Saint Augustine

Pain is a subjective feeling with objective roots and profound evolutionary biological utility. It reflects perceived or actual damage to the organism [1, 2]. Mental states can affect the perception of pain, and in turn be affected by pain. Psychiatric patients may have an increased perception of pain, as well as increased physical health reasons for pain, due to their often adverse life trajectory [3]. As such, they may be a particularly suitable population in which to try to identify peripheral blood biomarkers for pain, that may be complementary to genetic findings in the field [4]. Of note, a number of psychiatric medications are currently used to treat pain disorders [5]. Given these close inter-relationships, we expect our findings to be generalizable, trans-diagnostic, and have general relevance to pain, independent of specific psychiatric disorders.

First, we used a powerful longitudinal within-subject design in individuals with psychiatric disorders to discover blood gene expression changes between self-reported low pain and high pain states. Second, we prioritized the list of candidate biomarkers with a Bayesian-like Convergent Functional Genomics approach, comprehensively integrating previous published human and animal model evidence in the field for involvement in pain, and directly citing it. Third, we validated our top biomarkers from discovery and prioritization in an independent cohort of psychiatric subjects with a clinical diagnosis of a pain disorder and with high scores on pain severity and functional impact ratings. Fourth, we tested if the candidate biomarkers from the first three steps are able to predict high pain state, and future emergency department (ED) visits for pain, in another independent cohort of psychiatric subjects. We tested the biomarkers in all subjects in the independent test cohort, as well as in a more personalized fashion by gender and psychiatric diagnosis, showing increased accuracy with the personalized approach. Fifth, we assessed if our biomarkers have evidence for involvement in other psychiatric and related disorders, as well as analyzed the biological pathways and networks they are involved in. Sixth, we bioinformatically identified which of our individual biomarkers are modulated by existing drugs and thus can be used for pharmacogenomic population stratification and measuring of response to treatment, as well as used the gene expression signatures of the top predictive biomarkers to interrogate the Connectivity Map database from Broad/MIT to identify drugs and natural compounds that could be repurposed for treating pain.

## Materials and methods

### Cohorts

**Fig. 1:**
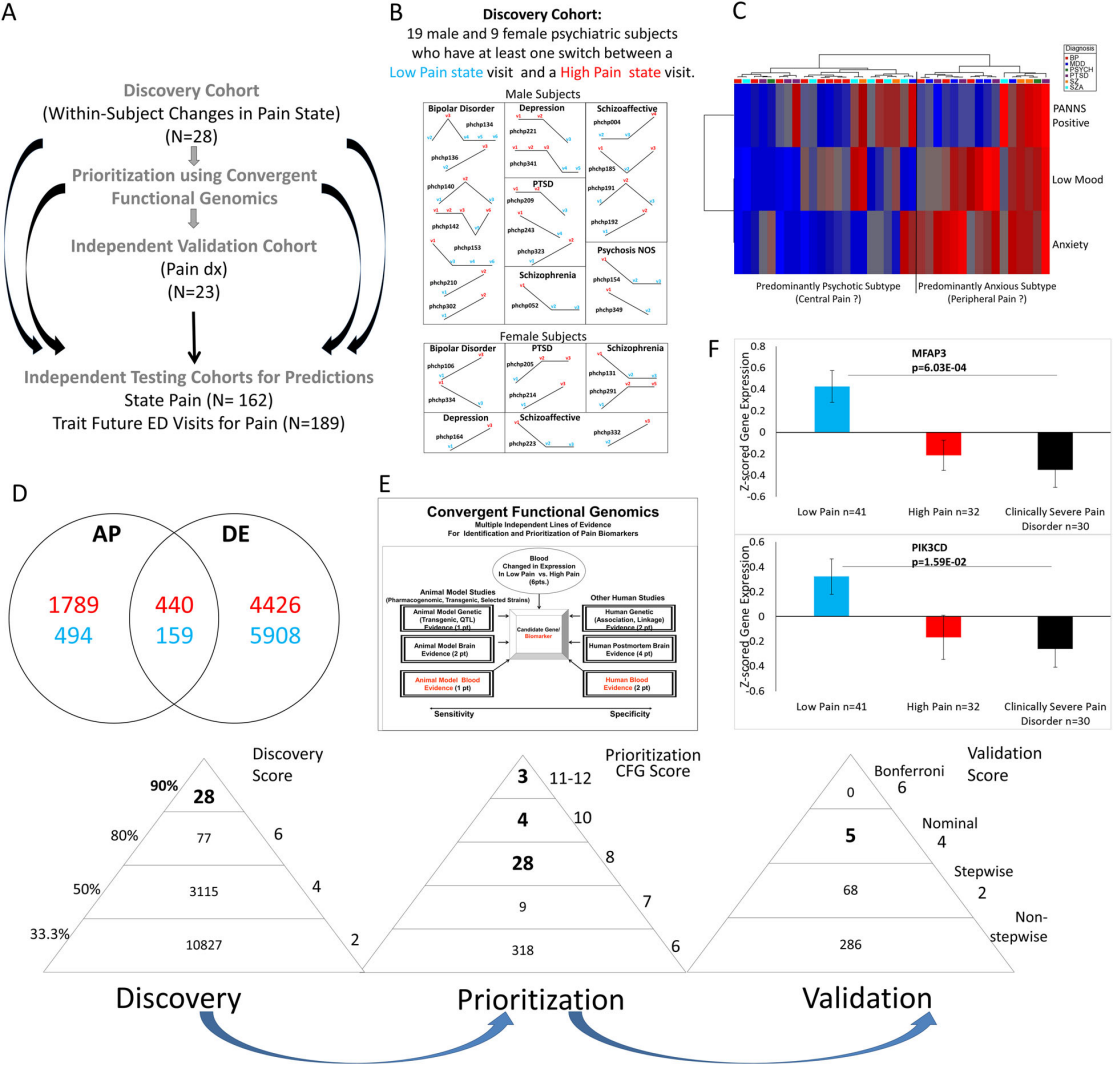
Steps 1–3: Discovery, prioritization, and validation. **a** Cohorts used in study, depicting flow of discovery, prioritization, and validation of biomarkers from each step. **b** Discovery cohort longitudinal within-subject analysis. Phchp### is study ID for each subject. V# denotes visit number. **c** Discovery of possible subtypes of Pain based on High Pain visits in the discovery cohort. Subjects were clustered using measures of mood and anxiety (Simplified Affective State Scale (SASS)), as well as psychosis (PANNS Positive). **d** Differential gene expression in the Discovery cohort—number of genes identified with differential expression (DE) and absent-present (AP) methods with an internal score of 2 and above. Red—increased in expression in High Pain, blue—decreased in expression in High Pain. At the discovery step probesets are identified based on their score for tracking pain with a maximum of internal points of 6 (33% (2 pt), 50% (4 pt), and 80% (6 pt)). **e** Prioritization with CFG for prior evidence of involvement in pain. In the prioritization step, probesets are converted to their associated genes using Affymetrix annotation and GeneCards. Genes are prioritized and scored using CFG for pain evidence with a maximum of 12 external points. Genes scoring at least six points out of a maximum possible of 18 total internal and external scores points are carried to the validation step. **f** Validation in an independent cohort of psychiatric patients with co-morbid pain disorders and severe subjective and functional pain ratings. In the validation step biomarkers are assessed for stepwise change from the discovery groups of subjects with Low Pain, to High Pain, to Clinically Severe Pain disorder, using ANOVA. *N* = number of testing visits. Five biomarkers were nominally significant, MFAP3 and PIK3CD were the most significant, and 68 biomarkers were stepwise changed

We used three independent cohorts: discovery (major psychiatric disorders), validation (major psychiatric disorders with clinically severe pain disorders), and testing (an independent major psychiatric disorders cohort for predicting pain state, and for predicting future ED visits for pain) (Fig. 1a).

Similar to our previous studies [6–8], the psychiatric subjects are part of a larger longitudinal cohort of adults that we are continuously collecting. Subjects were recruited from the patient population at the Indianapolis VA Medical Center. All subjects understood and signed informed consent forms detailing the research goals, procedure, caveats and safeguards, per Indiana University IRB approved protocol. Subjects completed diagnostic assessments by an extensive structured clinical interview—Diagnostic Interview for Genetic Studies, and up to six testing visits, 3–6 months apart or whenever a new psychiatric hospitalization occurred. At each testing visit, they received a series of rating scales, including a visual analog scale (1–10) for assessing pain and the SF 36 quality of life scale, which has two pain-related items (items 21 and 22), and the blood was drawn. We collected whole blood (10 ml) in two RNA-stabilizing PAXgene tubes, labeled with an anonymized ID number, and stored at −80 ºC in a locked freezer until the time of future processing. Whole-blood RNA was extracted for microarray gene expression studies from the PAXgene tubes, as detailed below.

For this study, our within-subject discovery cohort, from which the biomarker data were derived, consisted of 28 subjects (19 males, 9 females) with multiple testing visits, who each had at least one diametric change in pain from Low Pain (VAS of 2 and below) to High Pain (VAS of 6 and above) from one testing visit to another (Fig. 1b and Fig. S1). There were three subjects with five visits each, one subject with four visits, twelve subjects with three visits each, and twelve subjects with two visits each resulting in a total of 79 blood samples for subsequent gene expression microarray studies (Fig. 1 and Table S1).

Our validation cohort, in which the top biomarker findings were validated for being even more changed in expression, consisted of 13 male and 10 female subjects with a pain disorder diagnosis and clinically severe pain (Table S1). This was determined as having a pain VAS of 6 and above and a sum of SF36 scale items 21 (pain intensity) and 22 (impairment by pain of daily activities) of 10 and above (Table S1).

Our independent test cohort for predicting state (High Pain) consisted of 134 male and 28 female subjects with psychiatric disorders, demographically matched with the discovery cohort, with one or multiple testing visits in our lab, with either Low Pain, intermediate Pain, or High Pain, resulting in a total of 414 blood samples in which whole-genome blood gene expression data were obtained (Fig. 1 and Table S1).

Our test cohort for predicting trait (future ED visits with pain as the primary reason in the first year of follow-up, and all future ED visits for pain) (Fig. 1) consisted of 171 males and 19 female subjects for which we had longitudinal follow-up with electronic medical records. The subjects’ subsequent number of ED pain-related visits in the year following testing was tabulated from electronic medical records by a clinical researcher, who used the key word “pain” in the reasons for ED visit, or “ache” with a mention of acute pain in the text of the note.

#### Medications

The subjects in the discovery cohort were all diagnosed with various psychiatric disorders, and had various medical co-morbidities (Table 1). Their medications were listed in their electronic medical records, and documented by us at the time of each testing visit. Medications can have a strong influence on gene expression. However, our discovery of differentially expressed genes was based on within-subject analyses, which factor out not only genetic background effects but also minimizes medication effects, as the subjects rarely had major medication changes between visits. Moreover, there was no consistent pattern of any particular type of medication, as our subjects were on a wide variety of different medications, psychiatric and non-psychiatric. Some subjects may be non-compliant with their treatment and may thus have changes in medications or drug of abuse not reflected in their medical records. That being said, our goal is to find biomarkers that track pain, regardless if the reason for it is endogenous biology or driven by substance abuse or medication non-compliance. In fact, one would expect some of these biomarkers to be targets of medications, as we show in this paper. Overall, the discovery of biomarkers with our universal design occurs despite the subjects having different genders, diagnoses, being on various different medications, and other lifestyle variables.

**Table 1:** Aggregate Demographics

Cohorts	Number of subjects	Gender	Diagnosis	Ethnicity	Age at time of visit Mean (SD)	T-test for age
Discovery						
Discovery cohort (longitudinal withinsubject changes in pain scale (1–10)) Low Pain 0–2 to High Pain 6–10	28 (with 79 visits)	Male = 19 Female = 9	BP = 9 MDD= 3 SZA= 6 SZ= 3 PTSD= 5 PSYCH= 2	EA= 17 AA= 10 Mixed = 1	52 (7.94)	
Validation						
Independent validation cohort (clinical Severe pain diagnosis SF36 sum of scores on questions 21 and 22 ≥ 10 Pain scale ≥ 6)	23 (30 visits)	Male = 13 Female = 10	MDD=8 BP=6 SZ=2 SZA=2 PTSD=2 MOOD=3	EA= 17 AA= 6	51.9 (7.1)	
Testing						
Independent testing cohort for predicting State (High Pain State Pain Scale ≥6 at time of assessment)	162 (411 visits)	Male = 134 Female = 28	BP=52 MDD=39 SZA=19 SZ=26 PTSD=20 MOOD=4 PSYCH=2	EA= 112 AA= 48 Hispanic=2	50.3 (8.97) Others 50.12 High Pain 50.50	High Pain (n=101) Vs. Others (n=310) **0.824**
Independent testing cohort for predicting trait (Future ED visits for pain in the first year following assessment)	181 (470 visits)	Male = 163 Female = 18	BP = 46 MDD= 33 SZA= 45 SZ= 38 PTSD= 13 MOOD= 4 PSYCH= 2	EA= 117 AA= 62 Hispanic = 2	52.45 (6.13) Others 52.61 ED visits for Pain 51.87	ED visits for Pain (n=102) vs. Others (n=368) **0.237**
Independent testing cohort for predicting trait (Future ED visits for pain in All Years following assessment)	189 (501 visits)	Male = 170 Female = 19	BP = 49 MDD= 34 SZA= 45 SZ= 40 PTSD=15 MOOD= 4 PSYCH= 2	EA= 124 AA= 62 Hispanic=3	51.79 (6.75) Others 51.58 ED visits for Pain 52.02	ED visits for Pain (n=239) vs. Others (n=262) **0.4720**

*MDD* depression, *BP* bipolar, *SZ* schizophrenia, *SZA* schizoaffective, *PSYCHOSIS* schizophrenia and schizoaffective combined, *PTSD* post-traumatic stress disorder

### Blood gene expression experiments

#### RNA extraction

Whole blood (2.5–5 ml) was collected into each PaxGene tube by routine venipuncture. PaxGene tubes contain proprietary reagents for the stabilization of RNA. RNA was extracted and processed as previously described [6–8].

#### Microarrays

Microarray work was carried out using previously described methodology [6–9]. and as described below.

### Biomarkers

#### Step 1: Discovery

We have used the subject’s score from the VAS Pain Scale, assessed at the time of blood collection (Fig. 1). We analyzed gene expression differences between visits with Low Pain (defined as a score of 0–2) and visits with High Pain (defined as a score of 6 and above), using a powerful within-subject design, then an across-subjects summation (Fig. 1).

We analyzed the data in two ways: an Absent-Present (AP) approach, and a differential expression (DE) approach, as in previous work by us on suicide biomarkers [6–8]. The AP approach may capture turning on and off of genes, and the DE approach may capture gradual changes in expression. Analyses were performed as previously described [7–9]. We have developed in our labs R scripts to automate and conduct all these large dataset analyses in bulk, checked against human manual scoring [9].

Gene Symbol for the probesets were identified using NetAffyx (Affymetrix) for Affymetrix HG-U133 Plus 2.0 GeneChips, followed by GeneCards to confirm the primary gene symbol. In addition, for those probesets that were not assigned a gene symbol by NetAffyx, we used GeneAnnot (https://genecards.weizmann.ac.il/geneannot/index.shtml) to obtain gene symbols for these uncharacterized probesets, followed by GeneCard. Genes were then scored using our manually curated CFG databases as described below (Fig. 1e).

#### Step 2: Prioritization using Convergent Functional Genomics (CFG)

##### Databases

We have established in our laboratory (Laboratory of Neurophenomics, www.neurophenomics.info) manually curated databases of the human gene expression/protein expression studies (postmortem brain, peripheral tissue/fluids: CSF, blood and cell cultures), human genetic studies (association, copy number variations and linkage), and animal model gene expression and genetic studies, published to date on psychiatric disorders. Only findings deemed significant in the primary publication, by the study authors, using their particular experimental design and thresholds, are included in our databases. Our databases include only primary literature data and do not include review papers or other secondary data integration analyses to avoid redundancy and circularity. These large and constantly updated databases have been used in our CFG cross validation and prioritization platform (Fig. 1e). For this study, data from 355 papers on pain were present in the databases at the time of the CFG analyses (December 2017) (human genetic studies-212, human nervous tissue studies-3, human peripheral tissue/fluids- 57, non-human genetic studies-26, non-human brain/nervous tissue studies-48, non-human peripheral tissue/fluids- 9). Analyses were performed as previously described [7, 8].

#### Step 3: Validation analyses

Validation analyses of our candidate biomarker genes were conducted separately for AP and for DE. We examined which of the top candidate genes (total CFG score of 6 or above), were stepwise changed in expression from the Low Pain and High Pain group to the Clinically Severe Pain group. A CFG score of 6 or above reflects an empirical cutoff of 33.3% of the maximum possible CFG score of 12, which permits the inclusion of potentially novel genes with maximal internal score of 6 but no external evidence score. Subjects with Low Pain, as well as subjects with High Pain from the discovery cohort who did not have severe clinical pain (SF36 sum of item 21 and 22 < 10) were used, along with the independent validation cohort which all had severe clinical pain and a co-morbid pain disorder diagnosis (*n* = 23).

For the AP analyses, we imported the Affymetrix microarray.chp data files from the subjects in the validation cohort of Clinically Severe Pain into MAS5 Affymetrix Expression Console, alongside the data files from the Low Pain and High Pain groups in the live discovery cohort. We transferred the AP data to an Excel sheet and transformed *A* into 0, *M* into 0.5, and *P* into 1. We then *Z*-scored everything together by gender and diagnosis. If a probeset would have showed no variance and thus gave a non-determined (0/0) value in *Z*-scoring in a gender and diagnosis, we would have excluded the values from that probeset for that gender and diagnosis from the analysis.

For the DE analyses, the cohorts (Validation Clinically Severe Pain, alongside the Low Pain and High Pain groups in the Discovery cohort) were assembled out of Affymetrix .cel data that was RMA normalized by gender and diagnosis. We transferred the log transformed expression data to an Excel sheet, and non-log transformed the data by taking 2 to the power of the transformed expression value. We then *Z*-scored the values by gender and diagnosis.

We then imported the Excel sheets with the *Z*-scored by gender and diagnosis AP and DE expression data into Partek, and statistical analyses were performed using a one-way ANOVA for the stepwise changed probesets, and also attempted a stringent Bonferroni corrections for all the probesets tested (Figure 1F). We also wrote an R script that automatically analyzes the data directly from the Excel sheet, and used that to confirm our calculations.

### Choice of biomarkers to be carried forward

We carried forward into testing the top biomarkers from each step. The longer list of candidate biomarkers includes the top biomarkers from discovery step ( ≥ 90% of scores, *n* = 28), the top biomarkers from the prioritization step (CFG score ≥ 8, *n* = 32), and the nominally significant biomarkers after the validation step (*n* = 5), for a total of *n* = 65 probesets (*n* = 60 genes). The short list of top biomarkers after the validation step is five biomarkers. In Step 4, testing, we then predict with the biomarkers from the long list in independent cohorts High Pain state, and future ED visits for pain in the first year, and in all future years.

### Diagnostics

The test cohort for predicting High Pain (state), and the subset of it that is a test cohort for predicting future ED visits (trait), were assembled out of data that was RMA normalized by gender and diagnosis. The cohort was completely independent, there was no subject overlap with the discovery cohort. Phenomic (clinical) and gene expression markers used for predictions were *Z*-scored by gender and diagnosis, to be able to combine different markers into panels and to avoid potential artefacts due to different ranges of expression in different gender and diagnoses. Markers were combined by simple summation of the increased risk markers minus the decreased risk markers. Predictions were performed using R-studio.

#### Predicting state-high pain state

Receiver-operating characteristic (ROC) analyses between genomic and phenomic marker levels and Pain were performed by assigning subjects with a Pain score of 6 and greater into the High Pain category. We used the pROC package of R (Xavier Robin et al. BMC Bioinformatics 2011). We used the *Z*-scored biomarker and phene scores, running them in this ROC generating program against the diagnostic groups in the independent test cohort (High Pain vs. the rest of subjects). Additionally, a one-tailed t-test was performed between High Pain group vs. the rest, and Pearson R (one-tail) was calculated between Pain scores and marker levels (Supplementary Information- Complete Datasets and Analyses).

#### Predicting trait-future ED visits for pain in first year following testing

We conducted analyses for predicting ED visits for pain in the first year following each testing visit, in subjects that had at least 1 year of follow-up in the VA system, for which we have access to complete electronic medical records. ROC analyses between genomic and phenomic marker levels at a specific testing visit and future ED visits for pain were performed as described above, based on assigning if subjects had visited the ED with primary reason for pain or not within 1 year following a testing visit. Additionally, a one tailed *t*-test with unequal variance was performed between groups of subject visits with and without ED visits for Pain. Pearson R (one-tail) correlation was performed between hospitalization frequency (number of ED visits for Pain divided by duration of follow-up) and marker levels. A Cox regression was performed using the time in days from the testing visit date to first ED visit date in the case of patients who had been to the ED, or 365 days for those who did not. The hazard ratio was calculated such that a value >1 always indicates increased risk for ED visits, regardless if the biomarker is increased or decreased in expression.

We also conducted odds ratio analyses for ED visits for pain for all future ED visits due to pain, including those occurring beyond 1 year of follow-up, in the years following testing (on average 5.56 years per subject, range 0.44 to 11.27 years; see Table 1 and Table S1), as this calculation, unlike the ROC and *t*-test, accounts for the actual length of follow-up, which varied from subject to subject. The ROC and *t*-test might in fact, if used, under-represent the power of the markers to predict, as the more severe psychiatric patients are more likely to move geographically and/or be lost to follow-up. A Cox regression was also performed using the time in days from visit date to first ED Pain visit date in the case of patients who had been to the ED for Pain, or from visit date to last note date in the electronic medical records for those who did not. The hazard ratio was calculated such that a value >1 always indicates increased risk for ED Pain-related visits, regardless if the biomarker is increased or decreased in expression.

### Biological understanding

#### Pathway analyses

IPA (Ingenuity Pathway Analyses, version 24390178, Qiagen), David Functional Annotation Bioinformatics Microarray Analysis (National Institute of Allergy and Infectious Diseases) version 6.7 (August 2016), and Kyoto Encyclopedia of Genes and Genomes (KEGG) (through DAVID) were used to analyze the biological roles, including top canonical pathways and diseases (Table S4), of the candidate genes resulting from our work, as well as to identify genes in our dataset that are the target of existing drugs. We ran the pathway analyses for the combined AP and DE probesets 60 unique genes (65 probesets). For Network analysis of the 60 unique genes we performed STRING Interaction Network (https://string-db.org) by in putting the genes into the search window and performed Multiple Proteins Homo sapiens analysis.

#### CFG beyond Pain: evidence for involvement in other psychiatric and related disorders

We also used a CFG approach to examine evidence from other psychiatric and related disorders, for the long list of 65 candidate biomarkers (Table S3).

### Therapeutics

#### Pharmacogenomics

We analyzed which of our individual top biomarkers is known to be modulated by existing drugs using our CFG databases, and using Ingenuity Drugs analyses (Table S4).

#### New drug discovery/repurposing

We also analyzed which drugs and natural compounds are an opposite match for the gene expression profile of panels of our top biomarkers (*n* = 65), using the Connectivity Map (https://portals.broadinstitute.org, Broad Institute, MIT) (Table 3). Thirty-three out of 65 probesets were present in the HGU-133A array used for the Connectivity Map.

### Convergent functional evidence (CFE)

We tabulated into a convergent functional evidence (CFE) score all the evidence from discovery (up to 6 points), prioritization (up to 12 points), validation (up to 6 points), testing (state, trait first year ED visits, trait all future ED visits- up to 8 points each if significantly predicts in all subjects, 6 points if predicts by gender, 4 points if predicts in gender/diagnosis). The total score can be up to 48 points: 36 from our data and 12 from literature data. We weigh our data three times as much as the literature data. The goal is to highlight, based on the totality of our data and of the evidence in the field to date, biomarkers that have all around evidence: track pain, are reflective of pain pathology, and predict it. Such biomarkers merit priority evaluation in future clinical trials.

## Results

First, we used a discovery cohort composed of subjects with psychiatric disorders followed longitudinally over time [6–11], in which each subject had blood samples collected and neuropsychological testing done in at least one low pain state visit (Pain VAS ≤ 2 out of 10) and at least one high pain state visit (Pain VAS ≥ 6 out of 10) (Fig. 1 and Fig. S1).

We used a powerful longitudinal within-subject design [6–12] in individuals to discover blood gene expression changes between self-reported low pain and high pain states. A longitudinal within-subject design is orders of magnitude more powerful than a cross-sectional case-control design. Some of these candidate gene expression biomarkers are increased in expression in high pain states (being putative risk genes, or “*algogenes”*), and others are decreased in expression (being putative protective genes, or “*pain suppressor genes”*).

Second, we prioritized this list of candidate biomarkers with a Bayesian-like Convergent Functional Genomics approach [13, 14], comprehensively integrating previous published human and animal model evidence in the field for involvement in pain, and directly citing it.

Third, we further validated our top biomarkers from discovery and prioritization in an independent cohort of psychiatric subjects also carrying a clinical diagnosis of a pain disorder, and with high scores on pain severity and functional impairment ratings.

We ended up with a list of 65 candidate biomarkers (Table 2 and S2, S3, S5) from the first three steps, including a shorter list of five validated biomarkers (MFAP3, PIK3CD, SVEP1, TNFRSF11B, ELAC2). The biomarkers with the best evidence after validation were Hs.666804/MFAP3 (*p* = 6.03E-04) and PIK3CD (*p* = 1.59E-02).

**Table 2:** Convergent functional evidence (CFE) for top candidate biomarkers for pain (*n* = 60 genes, 65 probesets)

Gene symbol/gene name	Probesets	Step 1 Discovery in blood (direction of change) Method/ score/% Up to 6 pts	Step 2 External convergent functional genomics (CFG) Evidence for involvement in pain score Up to 12 pts	Step 3 Validation in blood ANOVA *p*-value/ score Up to 6 pts	Step 4 Best significant prediction of State-High Pain (Cases/Total) ROC AUC/ *p*-value 8 pts All 6 pts Gender 4 pts Gender/Dx	Step 4 Best Significant prediction of Trait-Future ED visits for Pain in the first year (Cases/Total) ROC AUC/ *p*-value 8 pts All 6 pts Gender 4 pts Gender/Dx	Step 4 Best significant predictions of Trait- Future ED visits for Pain in all future years (Cases/Total) OR/OR *p*-value 8 pts All 6 pts Gender 4 pts Gender/Dx	Step 5 Other psychiatric and related disorders evidence	Step 6 Drugs That Modulate the Biomarker in opposite Direction to Pain	CFE Polyevidence score for involvement in pain (based on steps 1–4)
*GNG7* G Protein subunit gamma 7	1566643_a_at	(D) DE/4 59%	6	6.81E-02/2 Stepwise	All C:(101/411) 0.56/3.52E-02 Gender Male C:(85/346) 0.56/3.95E-02 Gender/Dx M-SZ C:(11/64) 0.68/2.79E-02	Gender Females C:(7/44) 0.7/4.92E-02 Gender/Dx F-MDD C:(4/11) 0.82/4.45E-02 L:(2/6) 1/3.20E-02 F-PTSD C:(2/8) 0.92/4.78E-02	All C:(239/501) 1.28/**1.03E-04**^a^ L:(145/309) 1.22/1.70E-02 Gender Females C:(13/47) 1.69/4.69E-02 Males C:(226/454) 1.28/**1.92E-04**^**a**^ L:(138/282) 1.21/2.16E-02 Gender/Dx F-MDD C:(4/12) 14.54/2.23E-02 M-MDD L:(25/43) 1.8/2.70E-02 M-PSYCHOSIS C:(95/201) 1.52/**1.70E-04**^a^ L:(57/120) 1.34/2.47E-02 M-SZ C:(42/103) 1.58/2.08E-02 M-SZA C**:**(53/98) 1.71/**4.40E-04**^a^	Alcohol BP Hallucinogens MDD Stress SZ	Omega-3 fatty acids	34
*CNTN1* Contactin 1	1554784_at	(D) DE/4 52%	6	NS	All C:(101/411) 0.58/1.15E-02 L:(61/248) 0.63/1.42E-03 Gender Female C:(16/65) 0.65/3.38E-02 Male L:(51/212) 0.63/2.27E-03 Gender/Dx M-BP C:(24/123) 0.61/4.13E-02 L:(16/81) 0.64/4.06E-02 M-SZ C:(11/64) 0.68/3.15E-02 M-MDD L:(13/43) 0.66/4.53E-02 M-SZA L:(3/17) 0.83/3.89E-02	Gender Males C:(95/426) 0.56/3.08E-02	Gender/Dx M-MDD C**:**(42/72) 1.44/1.23E-02 L:(25/43) 1.64/4.17E-02	BP MDD SZ Suicide	Clozapine	28
*LY9* Lymphocyte antigen 9	231124_x_at	(I) DE/6 90%	2	NS	All C:(101/411) 0.56/4.40E-02 L:(61/248) 0.58/2.39E-02 Gender Male C:(85/346) 0.57/3.02E-02 L:(51/212) 0.62/5.19E-03 Gender/Dx M-BP C:(24/123) 0.63/2.66E-02 F-MDD C:(2/18) 0.97/1.75E-02 M-MDD L:(13/43) 0.8/9.87E-04	All C:(102/470) 0.56/2.30E-02 Gender Males C:(95/426) 0.59/2.61E-03 Gender/Dx M-BP C:(18/120) 0.68/6.91E-03 M-PTSD L:(10/16) 0.77/4.13E-02	Gender/Dx M-MDD C:(42/72) 1.65/3.85E-03 L:(25/43) 1.53/3.74E-02 M-PTSD L:(18/20) 2.07/6.77E-03	Acute stress	Omega-3 fatty acids	28
*CCDC144B* Coiled-coil domain containing 144B (pseudogene)	1557366_at	(D) DE/4 56%	6	NS	Gender/Dx F-BP C:(4/21) 0.79/3.66E-02 M-PSYCHOSIS C:(19/96) 0.68/8.95E-03 L:(10/56) 0.68/4.16E-02 M-SZA L:(3/17) 0.9/1.61E-02	Gender/Dx M-MDD C:(26/67) 0.63/3.43E-02	All C:(239/501) 1.23/2.27E-03 Gender Males C:(226/454) 1.23/3.34E-03 Gender/Dx M-PSYCHOSIS C:(95/201) 1.41/3.46E-03 L:(57/120) 1.43/1.32E-02 M-SZ C:(42/103) 1.84/4.65E-03 M-SZA L:(32/56) 1.47/3.49E-02			26
*GBP1* Guanylate binding protein 1	231578_at	(I) DE/2 37%	6	3.26E-01/2 Stepwise		All C:(102/470) 0.59/3.51E-03 Gender Females C:(7/44) 0.71/4.30E-02 Males C:(95/426) 0.58/1.04E-02 Gender/Dx F-MDD C:(4/11) 0.93/1.17E-02 M-PSYCHOSIS C:(33/198) 0.6/3.25E-02 M-SZA C:(23/97) 0.62/4.10E-02	All C:(239/501) 1.09/3.72E-02 Gender Females C:(13/47) 1.68/2.41E-02 Gender/Dx F-MDD C:(4/12) 3.1/4.43E-02 M-SZA C:(53/98) 1.22/3.65E-02	MDD PTSD SZ	Omega-3 fatty acids	26
*Hs.666804/ MFAP3* Microfibril associated protein 3	240949_x_at	(D) DE/6 81%	0	***6.03E-04/4 Nominal***	Gender/Dx F-PTSD C:(5/12) 0.8/4.41E-02	Gender/Dx M-BP L:(9/80) 0.75/7.27E-03	All L:(145/309) 1.28/2.28E-02 Gender Males C:(226/454) 1.17/2.64E-02 L:(138/282) 1.35/8.94E-03 Gender/Dx M-BP L:(34/91) 2.36/**4.86E-04**^a^ M-PTSD L:(18/20) 15.93/8.46E-04	Alcohol Suicide Stress		26
*CASP6* Caspase 6	209790_s_at	(I) DE/4 51%	4	NS	Gender Male L:(51/212) 0.59/2.92E-02 Gender/Dx F-MDD C:(2/18) 1/1.23E-02 M-MDD L:(13/43) 0.87/7.01E-05^a^	Gender Males C:(95/426) 0.57/2.54E-02 Gender/Dx M-PSYCHOSIS C:(33/198) 0.6/2.88E-02 M-SZA C:(23/97) 0.63/2.71E-02	Gender/Dx M-MDD C**:**(42/72) 1.31/3.97E-02	BP		24
*COMT* Catechol-O-methyltransferase	216204_at	(D) DE/4 54%	4	NS	Gender/Dx M-MDD L:(13/43) 0.71/1.41E-02	All C:(102/470) 0.55/4.48E-02 Gender Males C:(95/426) 0.57/1.95E-02 Gender/Dx M-MDD C:(26/67) 0.66/1.58E-02 M-PSYCHOSIS C:(33/198) 0.6/3.63E-02	Gender/Dx M-BP L:(34/91) 1.65/2.20E-02	ADHD Aggression Alcohol Anxiety BP Chronic stress MDD OCD Panic disorder Psychosis PTSD Suicide SZ	Clozapine Morphine Mood stabilizers	24
*RAB33A* RAB33A, member RAS oncogene family	206039_at	(I) DE/6 90%	0	NS	Gender/Dx F-MDD C:(2/18) 1/1.23E-02	Gender Males C:(95/426) 0.56/3.60E-02	All C:(239/501) 1.14/2.21E-02 Gender Males C:(226/454) 1.16/1.01E-02 Gender/Dx M-BP L:(34/91) 1.65/1.69E-03 M-MDD C:(42/72) 1.95/**6.59E-04**^a^ L:(25/43) 1.85/1.72E-02	Alcohol Stress MDD		24
*ZYX* Zyxin	238016_s_at	(D) DE/4 57%	4	NS	Gender/Dx F-BP C:(4/21) 0.78/4.44E-02	All C:(102/470) 0.55/4.80E-02 Gender Males C:(95/426) 0.57/1.58E-02 Gender/Dx M-PSYCHOSIS C:(33/198) 0.62/1.43E-02 M-SZA C:(23/97) 0.66/1.15E-02 M-BP L:(9/80) 0.71/2.26E-02	Gender/Dx M-BP L:(34/91) 1.85/1.67E-02 M-PTSD C:(26/31) 1.57/4.40E-02 L:(18/20) 2.2/1.53E-02	MDD	Clozapine	24
*(Hs.696420) MTERF1* Mitochondrial transcription termination factor 1	243125_x_at	(D) DE/6 100%	0	NS	Gender/Dx M-PSYCHOSIS C:(19/96) 0.67/1.01E-02 M-SZ C:(11/64) 0.77/2.27E-03 L:(7/39) 0.71/3.95E-02	Gender/Dx F-PTSD C:(2/8) 1/2.28E-02	All C:(239/501) 1.19/1.19E-02 L:(145/309) 1.2/4.81E-02 Gender Males C:(226/454) 1.19/1.51E-02 Gender/Dx M-PSYCHOSIS C:(95/201) 1.41/8.86E-03 M-SZ C:(42/103) 1.4/4.47E-02 M-SZA C:(53/98) 1.44/4.72E-02	PTSD Suicide		22
*COL27A1* Collagen type XXVII alpha 1 chain	225293_at	(D) DE/4 79%	4	7.47E-01/2 Stepwise	Gender/Dx M-MDD L:(13/43) 0.66/4.79E-02	Gender/Dx M-MDD C:(26/67) 0.63/3.38E-02 M-PSYCHOSIS C:(33/198) 0.61/2.79E-02 M-SZA C:(23/97) 0.68/4.96E-03 L:(13/55) 0.7/1.62E-02	Gender/Dx M-PTSD L**:**(18/20) 1.96/2.37E-02	Tourette syndrome	Lithium	22
*HRAS* HRas proto-oncogene, GTPase	212983_at	(I) DE/6 97%	0	NS	All C:(101/411) 0.56/3.47E-02 L:(61/248) 0.58/3.01E-02 Gender Male C:(85/346) 0.57/2.72E-02 L:(51/212) 0.61/1.18E-02 Gender/Dx M-SZ C:(11/64) 0.68/2.79E-02 M-MDD L:(13/43) 0.71/1.61E-02	Gender/Dx F-PTSD C:(2/8) 1/2.28E-02	Gender/Dx M-MDD C:(42/72) 2.2/3.38E-06^a^ L:(25/43) 2.25/**2.61E-04**^a^	Alcohol BP Longevity suicide SZ	ISIS 2503	22
*CALCA* Calcitonin related polypeptide alpha	210727_at	(D) DE/4 54%	7	NS	Gender Females C:(16/63) 0.66/3.12E-02 Gender/Dx F-MDD C:(2/18) 0.97/1.75E-02 F-BP L:(3/11) 0.88/3.31E-02 M-MDD L:(13/43) 0.66/4.79E-02	Gender/Dx F-PTSD C:(2/8) 1/2.28E-02 Gender/Dx M-PSYCHOSIS C:(33/198) 0.6/3.87E-02		Alcohol Anxiety Panic disorder	Omega-3 fatty acids Lithium	21
*(Hs.596713) PPP1R14B* Protein phosphatase 1 regulatory inhibitor subunit 14B	226138_s_at	(D) DE/6 90%	0	6.28E-02/2 Stepwise	Gender/Dx F-BP C:(4/21) 0.94/3.61E-03 L:(3/11) 0.92/2.06E-02 M-MDD L:(13/43) 0.73/9.98E-03		All C:(239/501) 1.15/1.43E-02 Gender Males C:(226/454) 1.19/4.84E-03 L:(138/282) 1.2/3.94E-02 Gender/Dx M-PSYCHOSIS C:(95/201) 1.35/3.06E-03 M-SZ C:(42/103) 1.53/3.19E-02 M-SZA C:(53/98) 1.41/9.26E-03	SZ	Lithium	20
*ASTN2* Astrotactin 2	1554816_at	(I) DE/6 83%	2	1.71E-01 /2 Stepwise		Gender/Dx F-MDD L:(2/6) 1/3.20E-02	Gender Female L:(7/27) 2.45/4.36E-02	Suicide SZ ASD BP MDD	Antipsychotics	20
*ELAC2* ElaC ribonuclease Z 2	201766_at	(D) DE/4 52%	2	***4.11E-02/4 Nominal***	Gender/Dx M-MDD L:(13/43) 0.73/8.66E-03		Gender Males L:(138/282) 1.2/4.61E-02 Gender/Dx M-BP L:(34/91) 1.55/4.79E-02 M-MDD C:(42/72) 1.69/2.47E-03 L:(25/43) 1.85/3.66E-02	ASD		20
*HLA-DQB1* Major histocompatibility complex, class II, DQ beta 1	212998_x_at	(I) DE/4 51%	8	NS	Gender/Dx M-SZ C:(11/64) 0.68/3.41E-02 F-MDD C:(2/18) 1/1.23E-02 M-MDD L:(13/43) 0.67/4.28E-02		Gender/Dx M-BP L:(34/91) 1.63/1.30E-02	Alcohol Depression Longevity stress Suicide SZ	Antipsychotics	20
*HLA-DQB1* Major histocompatibility complex, class II, DQ beta 1	211656_x_at	(I) DE/4 59%	8	NS	Gender/Dx F-MDD C:(2/18) 1/1.23E-02 M-SZ C:(11/64) 0.68/3.15E-02 M-SZ C:(11/64) 0.74/5.90E-03 L:(7/39) 0.72/3.36E-02 M-MDD L:(13/43) 0.69/2.68E-02 M-PSYCHOSIS L:(10/56) 0.69/3.29E-02	Gender/Dx M-MDD C:(26/67) 0.62/4.85E-02		Alcohol BP Depression Longevity PTSD Stress Suicide SZ	Antipsychotics	20
*PNOC* Prepronociceptin	205901_at	(I) DE/4 62%	4	NS	Gender/Dx M-SZ L:(7/39) 0.72/3.36E-02	Gender/Dx M-BP L:(9/80) 0.68/4.20E-02	Gender/Dx M-BP C:(53/134) 1.23/4.73E-02 L:(34/91) 1.26/2.67E-02 M-MDD C:(42/72) 1.4/2.09E-02	Addictions BP MDD SZ Stress		20
*TCF15* Transcription factor 15 (Basic helix-loop-helix)	207306_at	(D) DE/6 94%	2	NS	Gender/Dx F-MDD C:(2/18) 0.94/2.46E-02 M-MDD L:(13/43) 0.68/3.21E-02		All C:(239/501) 1.11/4.85E-02 Gender Males C:(226/454) 1.14/2.39E-02 Gender/Dx M-BP L:(34/91) 2.22/2.61E-03	Universal suicide Male suicide		20
*TOP3A* Topoisomerase (DNA) III alpha	214300_s_at	(D) DE/4 51%	4	NS	Gender/Dx F-BP C:(4/21) 0.84/1.97E-02		All L:(145/309) 1.18/4.66E-02 Gender Males L:(138/282) 1.2/3.88E-02 Gender/Dx M-SZ L:(25/64) 1.75/4.72E-02		Omega-3 fatty acids	20
*(H05785) LRRC75A* Leucine rich repeat containing 75A	236913_at	(D) AP/6 97%	0	NS	Gender/Dx F-MDD C:(2/18) 0.94/2.46E-02	All C:(102/470) 0.56/2.27E-02 L:(58/287) 0.58/3.38E-02 Gender Males C:(95/426) 0.57/1.64E-02 L:(54/261) 0.59/2.71E-02 Gender/Dx F-PTSD C:(2/8) 1/2.28E-02 M-PSYCHOSIS C:(33/198) 0.65/3.29E-03 M-SZA C:(23/97) 0.68/5.21E-03 M-SZA L:(13/55) 0.66/4.42E-02 M-MDD L:(16/39) 0.76/3.64E-03		Alcohol BP Suicide SZ	Clozapine	18
*CLSPN* Claspin	242150_at	(I) AP/6 95%	0	NS	Gender/Dx M-PSYCHOSIS C:(19/96) 0.65/2.48E-02	All L:(58/287) 0.57/4.62E-02 Gender/Dx F-MDD L:(2/6) 1/3.20E-02 M-MDD L:(16/39) 0.67/4.08E-02		Suicide		18
*COL2A1* Collagen type II alpha 1 chain	217404_s_at	(D) DE/4 54%	4	NS		Gender Males C:(95/426) 0.56/3.53E-02 Gender/Dx M-PSYCHOSIS C:(33/198) 0.63/7.32E-03 M-SZA C:(23/97) 0.66/1.08E-02 L:(13/55) 0.66/3.73E-02	Gender/Dx M-PTSD C:(26/31) 1.83/4.38E-03 L:(18/20) 2.3/1.08E-02	Aging		18
*HLA-DQB1* Major histocompatibility complex, class II, DQ beta 1	210747_at	(D) DE/2 44%	8	NS			All C:(239/501) 1.17/1.03E-02 Gender Males C:(226/454) 1.19/6.06E-03 Gender/Dx M-MDD C:(42/72) 1.35/3.68E-02 M-PSYCHOSIS C:(95/201) 1.26/1.33E-02 M-SZA C:(53/98) 1.33/2.06E-02	Addiction Stress	Benzodiazepines	18
*Hs.554262*	210703_at	(I) AP/6 100%	0	NS		All C:(102/470) 0.56/2.38E-02 L:(58/287) 0.58/2.49E-02 Gender Males C:(95/426) 0.56/4.18E-02 L:(54/261) 0.59/1.65E-02 Gender/Dx F-MDD C:(4/11) 0.82/4.45E-02 M-BP C:(18/120) 0.67/1.08E-02 M-MDD L:(16/39) 0.67/4.08E-02	Gender/Dx F-MDD C:(4/12) 7/4.47E-02 M-MDD L:(25/43) 2.13/7.30E-03	Suicide		18
*PIK3CD* Phosphatidylinositol-4,5-bisphosphate 3-kinase catalytic subunit delta	211230_s_at	(D) DE/6 83%	0	***1.59E-02/4 Nominal***			All C:(239/501) 1.13/3.18E-02 Gender Males C:(226/454) 1.14/2.71E-02 Gender/Dx M-BP C:(53/134) 1.3/2.85E-02 L:(34/91) 1.57/2.01E-02 M-MDD C:(42/72) 1.65/5.12E-03	Alcohol Chronic stress Longevity Suicide SZ	Clozapine Lithium Valproate	18
*SVEP1* Sushi, von willebrand factor type A, EGF And pentraxin domain containing 1	236927_at	(I) DE/2 49%	4	***2.17E-02/4 Nominal***	Gender/Dx F-PTSD C:(5/12) 0.8/4.41E-02 M-PTSD C:(13/38) 0.67/4.68E-02	Gender/Dx F-MDD C:(4/11) 0.82/4.41E-02		Addiction SZ	Omega-3 fatty acids	18
*TNFRSF11B* TNF receptor superfamily member 11b	204932_at	(D) DE/2 37%	4	***2.67E-02/4 Nominal***	Gender/Dx F-BP C:(4/21) 0.81/3.00E-02 M-MDD L:(13/43) 0.71/1.72E-02		Gender/Dx M-MDD C:(42/72) 1.42/4.25E-02 L:(25/43) 1.59/3.84E-02	Stress PTSD		18
*ZNF91* zinc finger protein 91	244259_s_at	(I) AP/6 95%	0	6.37E-01/2 Stepwise		Gender/Dx F-MDD C:(4/11) 0.93/1.17E-02	Gender Females C:(13/47) 2.12/1.03E-02 Gender/Dx F-BP C:(2/16) 4.21/4.55E-02 M-BP C:(53/134) 1.35/1.26E-02	Alcohol Circadian abnormalities PTSD		18
*CDK6* Cyclin dependent kinase 6	224851_at	(I) DE/4 56% (I) AP/2 42%	4	NS	Gender/Dx F-BP C:(4/21) 0.78/4.44E-02 L:(3/11) 1/7.15E-03	All C:(102/470) 0.57/1.03E-02 Gender Males C:(95/426) 0.59/5.57E-03 Gender/Dx M-MDD C:(26/67) 0.67/9.11E-03		Alcohol ASD Circadian abnormalities Longevity MDD SZ		17
*EDN1* Endothelin 1	1564630_at	(I) AP/4 56%	4	8.69E-02/2 Stepwise			Gender Females C:(13/47) 1.9/1.48E-02 Gender/Dx M-BP C:(53/134) 1.27/2.37E-02			16
*(AF090920) PPFIBP2* PPFIA binding protein 2	234739_at	(I) AP/6 94%	0	NS	Gender Female C:(16/65) 0.68/1.42E-02 L:(10/36) 0.69/3.87E-02 Gender/Dx F-PTSD C:(5/12) 0.8/4.41E-02		Gender/Dx M-PSYCHOSIS C:(95/201) 1.19/3.77E-02 M-SZ C:(42/103) 1.22/4.66E-02			16
*DCAF12* DDB1 and CUL4 associated factor 12	224789_at	(D) DE/6 86%	2	NS	Gender/Dx F-MDD C:(2/18) 1/1.23E-02		Gender/Dx M-BP C:(53/134) 1.61/4.42E-03	Cocaine Suicide	Omega-3 fatty acids Clozapine	16
*DNAJC18* DnaJ heat shock protein family (Hsp40) member C18	227166_at	(I) DE/6 94%	0	NS	Gender Female L:(10/36) 0.78/4.97E-03 Gender/Dx F-SZA L:(3/8) 0.93/2.63E-02 F-BP L:(3/11) 0.88/3.31E-02 F-PSYCHOSIS L:(3/8) 0.93/2.63E-02 F-PTSD L:(3/6) 1/2.48E-02	Gender/Dx F-MDD C:(4/11) 0.93/1.17E-02		BP		16
*HLA-DRB1* Major histocompatibility complex, class II, DR Beta 1	208306_x_at	(I) AP/4 52%	4	NS	Gender/Dx F-MDD C:(2/18) 0.91/3.39E-02 M-MDD L:(13/43) 0.66/4.79E-02 M-SZ L:(7/39) 0.71/4.27E-02	Gender/Dx M-SZA C:(23/97) 0.62/4.69E-02		Stress PTSD	Antipsychotics	16
*SEPT7P2* Septin 7 pseudogene 2	1569973_at	**(I)** **DE/6 100%** (I) AP/2 39%	0	NS	Gender Females C:(16/65) 0.65/3.27E-02 Gender/Dx F-PTSD C:(5/12) 0.97/3.69E-03 M-SZ C:(11/64) 0.77/2.83E-03		Gender/Dx M-MDD C:(42/72) 1.45/1.37E-02 L:(25/43) 2.25/**5.24E-04**^a^ M-PTSD C:(26/31) 2.38/**7.38E-04**^a^ L:(18/20) 3.59/1.77E-03	Suicide		16
*VEGFA* Vascular endothelial growth factor A	212171_x_at	(I) AP/4 65%	4	NS	Gender/Dx M-PSYCHOSIS C:(19/96) 0.66/1.78E-02 M-SZA C:(8/32) 0.7/4.48E-02		Gender/Dx M-MDD C:(42/72) 1.33/4.83E-02	BP MDD Stress SZ	Lithium Valproate Olanzapine	16
*WNK1* WNK lysine deficient protein kinase 1	1555068_at	(D) DE/6 92%	2	NS	Gender/Dx M-MDD L:(13/43) 0.77/2.75E-03		Gender/Dx M-BP C:(53/134) 1.41/3.18E-02	Alcohol Depression Suicide Methamphetamine Stress	Omega-3 Fatty acids SSRI	16
*(AF087971) PBRM1* Polybromo 1	1561067_at	(I) AP/6 90%	0	NS		All C:(102/470) 0.56/3.71E-02 Gender Males C:(95/426) 0.56/2.87E-02 Gender/Dx M-BP C:(18/120) 0.63/3.95E-02 M-PSYCHOSIS C:(33/198) 0.63/8.63E-03 M-SZA C:(23/97) 0.66/1.26E-02		BP Hallucinations Longevity MDD Methamphetamine Mood psychosis Stress Suicide		14
*(Hs.609761) SFPQ* Splicing factor proline and glutamine rich	244331_at	(D) DE/6 98%	0	NS	Gender/Dx M-SZ C:(11/64) 0.68/3.28E-02 L:(7/39) 0.75/2.21E-02		Gender/Dx M-MDD C:(42/72) 1.68/7.35E-03	Alcohol BP MDD Stress Suicide	Omega-3 fatty acids Clozapine Antidepressants Antipsychotics	14
*(Hs.659426) PHC3* Polyhomeotic homolog 3	240599_x_at	(D) DE/6 92%	0	NS	Gender/Dx F-MDD C:(2/18) 0.91/3.39E-02		Gender/Dx M-MDD C:(42/72) 1.48/1.83E-02	Suicide		14
*CCDC85C* Coiled-coil domain containing 85C	219018_s_at	(D) DE/6 94%	2	NS	Gender Female L:(10/36) 0.7/3.31E-02 Gender/Dx F-BP C:(4/21) 0.79/3.66E-02 L:(3/11) 0.92/2.06E-02 F-PTSD L:(3/6) 1/2.48E-02			Suicide		14
*GSPT1* G1 To S phase transition 1	215438_x_at	(D) DE/6 94%	0	NS	Gender/Dx F-MDD C:(2/18) 1/1.23E-02		Gender/Dx M-BP C:(53/134) 1.58/4.92E-03	BP Suicide MDD	Valproate	14
*HLA-DQB1* Major histocompatibility complex, class II, DQ Beta 1	211654_x_at	(I) DE/2 40%	8	NS	Gender/Dx M-PSYCHOSIS L:(10/56) 0.73/1.23E-02 M-SZ L:(7/39) 0.81/5.78E-03			Alcohol BP Depression Longevity PTSD Stress Suicide SZ	Antipsychotics	14
*LOXL2* Lysyl oxidase like 2	228808_s_at	(D) DE/4 59%	4	NS	Gender Females C:(16/65) 0.66/3.05E-02 Gender/Dx F-MDD C:(2/18) 1/1.23E-02			BP Suicide		14
*MBNL3* Muscleblind like splicing regulator 3	219814_at	(D) DE/6 92%	0	NS	Gender/Dx M-MDD L:(13/43) 0.71/1.51E-02		Gender/Dx M-BP C:(53/134) 1.43/8.16E-03	Psychosis Hallucination		14
*PTN* Pleiotrophin	211737_x_at	(D) DE/6 92%	0	NS			All C:(239/501) 1.16/1.17E-02 Gender Males C:(226/454) 1.2/4.66E-03 Gender/Dx M-PSYCHOSIS C:(95/201) 1.24/1.98E-02 M-SZA C:(53/98) 1.35/1.28E-02	SZ Stress Suicide	Omega-3 fatty acids Risperidone	14
*RALGAPA2* Ral GTPase activating protein catalytic alpha subunit 2	231826_at	(D) DE/6 97%	0	NS	Gender/Dx F-MDD C:(2/18) 0.94/2.46E-02		Gender/Dx M-MDD C:(42/72) 2.06/**4.52E-04**^a^ L:(25/43) 2.05/5.35E-03	BP		14
*YBX3* Y-Box binding protein 3	201160_s_at	(D) DE/6 94%	0	NS	Gender/Dx F-MDD C:(2/18) 0.97/1.75E-02		Gender/Dx M-BP C:(53/134) 1.39/1.23E-02	BP Suicide SZ	Mianserin	14
*ZNF441* Zinc finger protein 441	1553193_at	(I) AP/6 95% (I) DE/2 35%	0	NS		Gender/Dx M-SZA L:(13/55) 0.67/3.13E-02	Gender/Dx M-MDD L:(25/43) 1.72/1.92E-02			14
*CCND1* Cyclin D1	208712_at	(D) DE/4 57%	4	NS			Gender/Dx M-BP C:(53/134) 1.33/4.53E-02	Addiction MDD Stress Hallucinogens		12
*CDK6* Cyclin-dependent kinase 6	224847_at	(I) DE/4 63%	4	NS			Gender/Dx M-PTSD L:(18/20) 2.09/1.75E-02	Alcohol ASD Circadian abnormalities Longevity MDD SZ		12
*COMT* Catechol-O-methyltransferase	213981_at	(D) DE/4 54%	4	NS	Gender/Dx M-MDD L:(13/43) 0.71/1.41E-02			ADHD Aggression Alcohol Anxiety BP Chronic stress MDD OCD Panic disorder Psychosis PTSD Suicide SZ	Clozapine Morphine Mood stabilizers	12
*HTR2A* 5-Hydroxytryptamine receptor 2A	211616_s_at	(D) DE/4 52%	4	NS	Gender/Dx M-BP L:(16/81) 0.65/2.89E-02			Addictions Aging Alcohol Anxiety BP Depression MDD Mood disorders NOS OCD Panic disorder PTSD Stress Suicide SZ		12
*NF1* Neurofibromin 1	212676_at	(I) DE/4 59%	4	NS	Gender/Dx F-BP L:(3/11) 0.92/2.06E-02			Addiction BP PTSD	Fluoxetine SSRI	12
*SHMT1* Serine hydroxymethyltransferase 1	217304_at	(D) DE/2 43%	6	NS		Gender/Dx F-PTSD C:(2/8) 1/2.28E-02 M-SZA L:(13/55) 0.7/1.54E-02		Suicide	Clozapine	12
*TSPO* Translocator protein	202096_s_at	(I) DE/2 38%	6	NS	Gender/Dx M-SZ C:(11/64) 0.72/1.06E-02			SZ		12
*DENND1B* DENN domain containing 1B	1557309_at	(I) DE/6 90%; (I) AP/2 40%	0	NS	Gender/Dx M-SZA L:(3/17) 0.83/3.89E-02				Omega-3	10
*MCRS1* Microspherule protein 1	202556_s_at	(I) DE/6 90%	0	NS	Gender/Dx M-MDD L:(13/43) 0.75/5.16E-03			MDD		10
*OSBP2* Oxysterol binding protein 2	1569617_at	(D) DE/6 94%	0	NS	Gender/Dx F-MDD C:(2/18) 1/1.23E-02			Cocaine Suicide SZ		10
*FAM134B* Family with sequence similarity 134 member B	218510_x_at	(I) DE/4 51%; (I) AP/2 34%	4	NS				Antisocial personality Suicide	Omega-3 fatty acids	8
*ZNF429* Zinc finger protein 429	1561270_at	(D) DE/2 37%	6	NS						8
*(Hs.677263) SMURF2* SMAD specific E3 ubiquitin protein ligase 2	216444_at	(D) AP/6 100% (D) DE/4 71%	0	NS				Aging Suicide Stress		6

*DE* differential expression, *AP* absent/present, *NS* non-stepwise in validation. For predictions, *C*-cross-sectional (using levels from one visit), *L*-longitudinal (using levels and slopes from multiple visits). In All, by Gender, and personalized by Gender and Diagnosis (Gender/Dx). *M* males, *F* Females, *MDD* depression, *BP* bipolar, *SZ* schizophrenia, *SZA* schizoaffective, *PSYCHOSIS* schizophrenia and schizoaffective combined, *PTSD* post-traumatic stress disorder.

^a^Significant after Bonferroni correction for the number of biomarkers tested (65). For Steps 2, 5 and 6, see Supplementary Information tables for citations for the evidence.

**Fig. 2:**
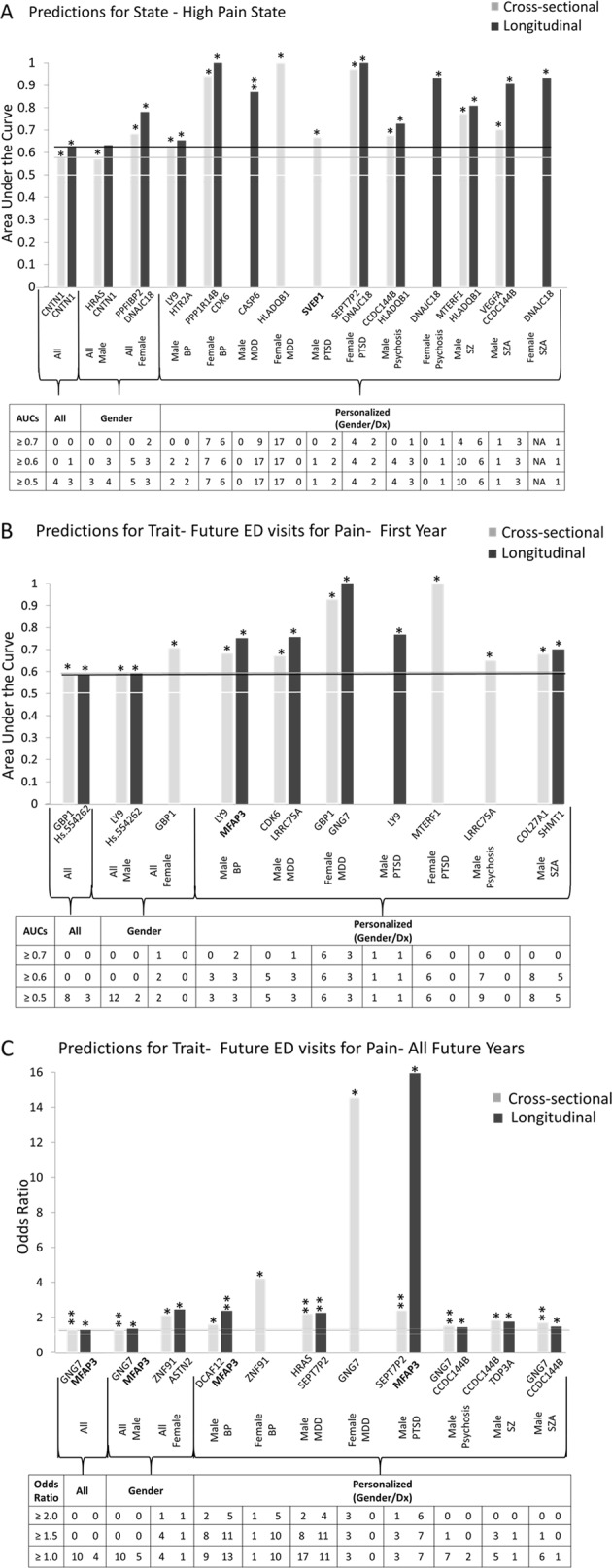
Best single biomarkers predictors. From the long list (*n* = 65). Those on short list (*n* = 5) are bolded. Bar graph shows best predictive biomarkers in each group. *Nominally significant *p* < 0.05. **Bonferroni significant for the 65 biomarkers tested. Table underneath the figures displays the actual number of biomarkers for each group whose ROC AUC *p*-values (**a**, **b**) and Cox odds ratio *p*-values (**c**) are at least nominally significant. Some female diagnostic group are missing from the graph as they did not have any significant biomarkers. Cross-sectional is based on levels at one visit. Longitudinal is based on levels at multiple visits (integrates levels at most recent visit, maximum levels, slope into most recent visit, and maximum slope). Dividing lines represent the cutoffs for a test performing at chance levels (white), and at the same level as the best biomarkers for all subjects in cross-sectional (gray) and longitudinal (black) based predictions. All biomarkers perform better than chance. Biomarkers performed better when personalized by gender and diagnosis

Fourth, we tested if the 65 candidate biomarkers are able to predict pain severity state, and future emergency department (ED) visits for pain, in another independent cohort of psychiatric subjects. We used biomarker levels information cross-sectionally, as well as expanded longitudinal information about biomarker levels at multiple visits, as predictors. We tested the biomarkers in all subjects in the test cohort, as well as in a more personalized fashion by gender and psychiatric diagnosis, showing increased accuracy with the personalized approach, in particular in women (Fig. 2). Across all subjects tested, CNTN1 was the best predictor for state (AUC 63%, *p* = 0.0014), GBP1 the best predictor for trait first year ED visits (AUC 59%, *p* = 0.0035), and GNG7 the best predictor for trait all future ED visits (OR 1.28, *p* = 0.00013, surviving Bonferroni correction for the 65 biomarkers tested). By gender, in females, DNAJC18 was the best predictor for state (AUC 78%, *p* = 0.0049), GBP1 the best predictor for trait first year ED visits (AUC 71%, *p* = 0.043) and ASTN2 for trait all future ED visits (OR 2.45, *p* = 0.044). In males, CNTN1 was the best predictor for state (AUC 63%, *p* = 0.0022), Hs.554262 the best predictor for trait first year ED visits (AUC 59%, *p* = 0.016), and MFAP3 the best predictor for trait all future ED visits (OR 1.35, *p* = 0.0089). Personalized by gender and diagnosis, in female bipolar CDK6 was a strong predictor for state (AUC 100%, *p* = 0.007), in female PTSD SHMT1 was a strong predictor for trait first year ED visits (AUC 100%, *p* = 0.022), and in female depression GNG7 for trait all future ED visits (OR 14.54, *p* = 0.022). In male depression CASP6 was a strong predictor for state (AUC 87%, *p* = 0.00007, surviving Bonferroni correction for the 65 biomarkers tested), in male PTSD LY9 was a strong predictor for trait first year ED visits (AUC 77%, *p* = 0.041), and in male PTSD MFAP3 was a strong predictor for trait all future ED visits (OR 15.93, *p* = 0.00085). In general, panels of all 65 top biomarkers or of the five validated biomarkers did not work as well as individual biomarkers, particularly when the later are tested by gender and diagnosis, consistent with there being heterogeneity in the population and supporting the need for personalization. The notable exception was predicting longitudinally all future ED visits for pain, where the panel of five validated biomarkers performed better than individual biomarkers (Supplementary Information—Complete Analyses). Importantly, predictions of future ED visits for pain in the independent cohorts were consistently stronger using biomarkers than clinical phenotypic markers (pain VAS scale, pain items 21 and 22 from SF 36) (see Supplementary Information—Complete Analyses), supporting the utility of biomarkers.

**Fig. 3:**
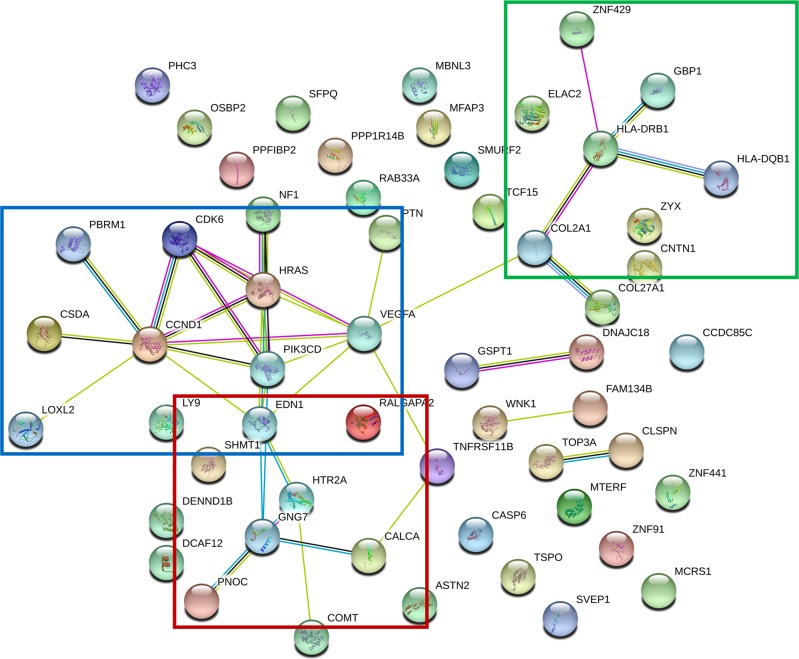
Biological roles. STRING interaction network for the top biomarkers for pain (65 probesets, 60 genes)

Fifth, we assessed if our biomarkers have evidence for involvement in other psychiatric and related disorders (Table S3). A majority of our biomarkers have some evidence in other disorders, whereas a few seem to be specific for pain, such as CCDC144B (Coiled-Coil Domain Containing 144B), COL2A1 (Collagen Type II Alpha 1 Chain), PPFIBP2 (PPFIA Binding Protein 2), DENND1B (DENN Domain Containing 1B), ZNF441 (Zinc Finger Protein 441), TOP3A (Topoisomerase (DNA) III Alpha), and ZNF429 (Zinc Finger Protein 429). A majority of our biomarkers (50 out of 60 genes, i.e., 83.3%) have prior evidence for involvement in suicide, suggesting an extensive molecular co-morbidity between pain and suicide, to go along with the clinical and phenomenological co-morbidity (physical pain, psychic pain) [15]. We also analyzed the biological pathways and networks our biomarkers are involved in (Table S4 and Fig. 3). There is a network centered on GNG7 (Fig. 3), that may be involved in connectivity/signaling, comprising HTR2A, EDN1, PNOC (involved in pain signaling) and CALCA (involved in Reflex Sympathetic Dystrophy and Complex Regional Pain Syndrome). It is reassuring that we see PNOC (Prepronociceptin) increased in expression in high pain states, i.e., as an algogene. Given its known roles in pain, it serves as a de facto positive control. A second network is centered on CCND1, may be involved in activity/trophicity, and comprises HRAS, CDK6, PBRM1, CSDA, LOXL2, EDN1, PIK3CD, and VEGFA. A third network is centered on HLA DRB1, may be involved in reactivity/immune response, and comprises GBP1, ZNF429, COL2A1, and HLA DQB1, from our list of 65 top biomarkers.

Sixth, we identified which of our biomarkers are targets of existing drugs and thus can be used for pharmacogenomics population stratification and measuring of response to treatment (Table 2 and Table S5), as well as used the biomarker gene expression signature to interrogate the Connectivity Map database from Broad/MIT to identify drugs and natural compounds that can be repurposed for treating pain (Table 3). The top drugs identified as potential new pain therapeutic is SC-560, an NSAID, haloperidol, and antipsychotic, and amoxapine, an antidepressant. The top natural compounds were pyridoxine (vitamin B6), cyanocobalamin (vitamin B12), and apigenin (a plant flavonoid).

**Table 3:** Therapeutics

Rank	CMAP name	Score	Description
1	**SC-560**	−1	SC-560 is an NSAID, member of the diaryl heterocycle class of cyclooxygenase (COX) inhibitors which includes celecoxib (Celebrex™) and rofecoxib (Vioxx™). However, unlike these selective COX-2 inhibitors, SC-560 is a selective inhibitor of COX-1.
2	*Pyridoxine*	−0.997	Pyridoxine is the 4-methanol form of vitamin B6 and is converted to pyridoxal 5-phosphate in the body. Pyridoxal 5-phosphate is a coenzyme for synthesis of amino acids, neurotransmitters (serotonin, norepinephrine), sphingolipids, aminolevulinic acid.
3	Methylergometrine	−0.975	Methylergometrine is a synthetic analog of ergonovine, a psychedelic alkaloid found in ergot, and many species of morning glory. It is chemically similar to LSD, ergine, ergometrine, and lysergic acid. Due to its oxytocic properties, it has a medical use in obstetrics.
4	LY-294002	−0.923	LY-294002 is a potent, cell permeable inhibitor of phosphatidylinositol 3-kinase (PI3K) that acts on the ATP binding site of the enzyme. The PI3K pathway has a role in inhibiting apoptosis in cancer. PI3K is also known to regulate TLR-mediated inflammatory responses.
5	Haloperidol	−0.917	Widely used typical antipsychotic medication
6	Cytisine	−0.909	Like varenicline, cytisine is a partial agonist of nicotinic acetylcholine receptors (nAChRs), with an affinity for the α4β2 receptor subtype, and a half-life of 4.8 h.
7	*Cyanocobalamin*	−0.902	Cyanocobalamin is a form of vitamin B12. Vitamin B12 is important for growth, cell reproduction, blood formation, and protein and tissue synthesis.
8	*Apigenin*	−0.899	Apigenin (4′,5,7-trihydroxyflavone), found in many plants such as chamomile, is a natural product belonging to the flavone class. Apigenin acts as a monoamine transporter activator, and is a weak ligand for central benzodiazepine receptors in vitro and exerts anxiolytic and slight sedative effects in an animal model. It has also effects on adenosine receptors and is an acute antagonist at the NMDA receptors (IC50 = 10 μM). In addition, like various other flavonoids, apigenin has been found to possess nanomolar affinity for the opioid receptors, acting as a non-selective antagonist of all three opioid receptors.
9	***Beta-escin***	−0.892	Escin, a natural mixture of triterpenoid saponins isolated from horse chestnut (*Aesculus hippocastanum*) seeds, is used and studied as a vasoprotective anti-inflammatory, anti-edematous, and anti-nociceptive agent.
13	**Amoxapine**	−0.875	Amoxapine is a tricyclic antidepressant of the dibenzoxazepine class. This drug is used to treat symptoms of depression and neuropathic pain.

New drug discovery/repurposing. Connectivity Map [43, 44] (CMAP) analysis- drugs that have *opposite* gene expression profile effects to our pain biomarkers signatures (i.e., best is -1). Out of 65 probesets, 14 of the 29 increased, and 19 of the 36 decreased were present in HG-U133A array used by Connectivity Map. A score of −1 indicates the perfect opposite match, i.e., the best potential therapeutic for pain. Drugs in Bold—drugs known to treat pain, which thus serve as a de facto positive control for our approach. Drugs in Italic—natural compounds

## Discussion

Biomarkers are emerging as important tools in disorders where subjective self-report of an individual and/or clinical impression of a healthcare professional are not always reliable. Recent work by our group has identified blood gene expression biomarkers that track suicidality using powerful longitudinal within-subject designs, validated them in suicide completers, and tested them in independent cohorts demonstrating their ability to predict state (suicidal ideation), and to predict trait (future hospitalizations for suicidality) (Niculescu et al. [7], Levey et al. [2], Niculescu et al. [8, 11]). Similar to suicidality, pain is a subjective feeling, with objective roots. It may reflect past or current injury events, their adverse consequences and compensatory mechanisms. The rationale for identifying validated and reproducible blood biomarkers is precisely because you cannot directly biopsy brain and spinal centers of pain perception. Blood biomarkers are easily accessible, and constitute a surrogate (liquid biopsy).

We present work employing a powerful longitudinal within-subject design, previously described by us for suicidality [6–12], and used now to discover blood gene expression changes between self-reported low pain and high pain states. Gene expression is more powerful than genetics, as it integrates a multitude of genetic variants and environmental effects. Longitudinal within-subject designs are more powerful than case-control designs, and can provide information with small Ns, as illustrated and discussed by Snyder and colleagues [16–18], as well as Schork, Topol, and colleagues [19, 20]. Some of these candidate gene expression biomarkers are increased in expression in high pain states (being putative risk genes), and others are decreased in expression (being putative protective/resilience genes). We cannot readily differentiate with our observational studies which of them are a reflection of damage and which are compensatory mechanisms. However, given the fact that these biomarkers are discovered in Step 1 by tracking present/state changes in the perception of pain and not past/trait exposure, they may be more likely a reflection of pathogenesis rather than adaptation.

Our systematic approach led to the identification of objective predictive biomarkers for pain, state and trait. We present evidence for universal biomarkers for pain, as well as show evidence that personalization by gender and diagnosis enhances precision, going from AUCs over 60% to AUCs over 80%. A majority of the top biomarkers we have identified overlap with biomarkers previously identified by us in suicide, and almost all have evidence in other psychiatric disorders (Table S3). Overlap and co-morbidity of genetic findings are in general the rule, not the exception, for neuropsychiatric disorders. Given the fact that pain disorders and psychiatric disorders are highly co-morbid clinically, that psychiatric medications are used to treat pain, and that pain medications can have psychiatric effects, the overlap is perhaps not surprising. It underlies the co-morbidity and impact of pain on mental health and on suicidality. Indeed, mood disorders were among the top diseases identified by pathway analyses of our pain biomarker data (Table S4B).

The biomarkers with the best overall convergent information evidence (CFE) across the multiple steps were GNG7, CNTN1, LY9, CCDC144B, GBP1, and MFAP3 (Table 2). GNG7 (G Protein Subunit Gamma 7), with roles in signal transduction, is decreased in expression in blood in High Pain states in our work, i.e., it is a pain suppressor gene. GNG7 is a strong predictor in the independent cohorts, particularly for all future ED visits for pain. There is evidence in other tissues in human studies for involvement in pain (diabetic neuropathy [21], vertebral disc [22]). GNG7 is a strong predictor in the independent cohorts, particularly for all future ED visits for pain. GNG7 also has trans-diagnostic evidence for involvement in other psychiatric disorders. It is decreased in expression in mouse brain by alcohol, hallucinogens, and stress [23, 24, 25], and increased in expression by omega-3 fatty acids. CNTN1 (Contactin 1), with roles in neuronal cell adhesion, is decreased in expression in blood in High Pain states in our work, i.e., it is a pain suppressor gene. Reassuringly, there is a possible mechanistic basis for its involvement in pain [26], and there is convergent evidence in other tissues in human studies for involvement in pain: CNTN1 has also been reported to be decreased in expression in CSF in women with chronic widespread pain (CWP) [27]. Anti-contactin 1 autoantibodies, that block/decrease levels of contactin 1, have been described in chronic inflammatory demyelinating polyneuropathy [28]. Such reproducibility across studies, tissues and populations provides strong reasons to consider it as a bona fide marker for pain, and it serves as a reassuring de facto positive control for the design and power of our study. CNTN1 has also trans-diagnostic evidence for involvement in psychiatric disorders. It is decreased in expression in schizophrenia brain [29] and blood [30], and in blood in suicidality in females [8]. CNTN1 is increased in expression by clozapine in mouse brain [24]. LY9 (lymphocyte antigen 9), with immunomodulatory roles, is increased in expression in blood in High Pain states in our work, i.e., it is an algogene. LY9 is a good predictor in the independent cohorts for state and trait, particularly for males with MDD and PTSD. It also has epigenetic evidence for involvement in exposure to stress [31], and is decreased in expression by omega-3 fatty acids in mouse brain [32]. CCDC144B (Coiled-Coil Domain Containing 144B) is decreased in expression in blood in High Pain states in our work. There is evidence in other tissues in human [33] and animal model [34] studies for involvement in pain. CCDC144B is a good predictor in the independent cohorts for state and trait, particularly for males with psychosis (SZ, SZA). It does not have trans-diagnostic evidence for involvement in other psychiatric disorders, seeming to be relatively specific for pain. GBP1 (guanylate binding protein 1), with interferon induced signaling roles, is increased in expression in blood in High Pain states in our work. There is other evidence in human studies, gene expression [33] and genetic [35], for involvement in pain. GBP1 is a predictor in the independent cohorts for trait, particularly in females. It is increased in expression in the brain in MDD [36], schizophrenia [37, 38], and suicide [36], and in blood in PTSD [39]. GBP1 is decreased in expression by omega-3 in mouse brain [40]. Hs.666804/MFAP3 (microfibril associated protein 3), another of the top markers, is a component of elastin-associated microfibrils. MFAP3 has the most robust empirical evidence from our discovery and validation steps, and is a strong predictor in the independent cohort, particularly for pain in females and males with PTSD. Interestingly, it has no prior evidence for pain in the literature curated to date for our Prioritization/CFG step, which demonstrates that we are casting a wide-enough net with our approach that can bring to the fore completely novel findings. MFAP3 is decreased in expression in blood in High Pain states in our work, i.e., it is a pain suppressor gene. It also has previous evidence for involvement in alcoholism [41], stress [42], and suicide [7, 9].

A phenotypic clustering analysis of the discovery cohort revealed two broad putative subtypes of High Pain states, a predominantly *psychotic subtype*, possibly related to mis-connectivity and increased perception of pain *centrally*, and a predominantly *anxious subtype*, possibly related to reactivity and increased physical health reasons for pain *peripherally*. Deeper analyses of the clustering in future studies may also substantiate further parsing of the subtypes, possibly into eight instead of only two subtypes, and of underlying differentiating biomarkers.

The biomarkers gene expression signatures also open the door to drug repurposing approaches, including of nutraceuticals. Nutraceuticals are particularly amenable to use in preventive population level approaches.

In conclusion, our work opens the door for precision medicine for pain, with objective diagnostics and targeted novel therapeutics. Given the massive negative impact of untreated pain on quality of life, the current lack of objective measures to determine appropriateness of treatment, and the severe addiction gateway potential of existing opioid-based pain medications, the importance of approaches such as ours cannot be overstated.

## Supplementary information

Detailed Demographics Tables S1

Figure S1 and Tables S2-S5

Complete Data and Analyses
