# Supplementary material for: Towards precision medicine for pain: diagnostic biomarkers and repurposed drugs
Source: Mol Psychiatry. 2019 Feb 12;24(4):501–22. doi: 10.1038/s41380-018-0345-5 (PMC6477790; doi:10.1038/s41380-018-0345-5)
Supplement: Supplementary file 1 — Detailed Demographics Tables S1 [file 41380_2018_345_MOESM1_ESM.docx]

**Table S1 Detailed Demographics**

| **Discovery Cohort (n=28) (79 visits)** | | | | | | | | | |
| --- | --- | --- | --- | --- | --- | --- | --- | --- | --- |
| **Participant ID and visit number** | **Diagnosis** | **Gender** | **Age** | **Ethnicity** | **Pain Scale(1-10)** | **Assignment** | **SF-36 Pain (Q21)** | **SF-36 Pain (Q22)** | **SF-36 Pain (Q21+ Q22) SEVERE>=10** |
| phchp004v3 | SZA | M | 60 | African American | 0 | Low Pain | 3 | 3 | 6 |
| phchp052v2 | SZ | M | 60 | Caucasian | 0 | Low Pain | 4 | 4 | 8 |
| phchp052v3 | SZ | M | 60 | Caucasian | 0 | Low Pain | 5 | 5 | 10 |
| phchp131v2 | SZ | F | 55 | African American | 0 | Low Pain | 1 | 1 | 2 |
| phchp131v3 | SZ | F | 56 | African American | 0 | Low Pain | 3 | 3 | 6 |
| phchp134v2 | BP | M | 59 | Caucasian | 0 | Low Pain | 4 | 4 | 8 |
| phchp134v5 | BP | M | 62 | Caucasian | 0 | Low Pain | 4 | 4 | 8 |
| phchp134v6 | BP | M | 62 | Caucasian | 0 | Low Pain | 4 | 4 | 8 |
| phchp136v2 | BP | M | 41 | Caucasian | 0 | Low Pain | 5 | 5 | 10 |
| phchp140v1 | BP | M | 38 | Caucasian | 0 | Low Pain | 3 | 3 | 6 |
| phchp140v3 | BP | M | 38 | Caucasian | 0 | Low Pain | 4 | 4 | 8 |
| phchp142v5 | BP | M | 57 | Caucasian | 0 | Low Pain | 5 | 5 | 10 |
| phchp153v4 | BP | M | 57 | Caucasian | 0 | Low Pain | 1 | 1 | 2 |
| phchp153v6 | BP | M | 58 | Caucasian | 0 | Low Pain | 2 | 2 | 4 |
| phchp154v2 | PSYCH | M | 51 | African American | 0 | Low Pain | 3 | 3 | 6 |
| phchp154v3 | PSYCH | M | 52 | African American | 0 | Low Pain | 3 | 3 | 6 |
| phchp185v2 | SZA | M | 51 | African American | 0 | Low Pain | 1 | 1 | 2 |
| phchp191v1 | SZA | M | 58 | African American | 0 | Low Pain | 1 | 1 | 2 |
| phchp191v3 | SZA | M | 59 | African American | 0 | Low Pain | 1 | 1 | 2 |
| phchp205v1 | PTSD | F | 54 | Caucasian | 0 | Low Pain | 3 | 3 | 6 |
| phchp209v3 | PTSD | M | 54 | African American | 0 | Low Pain | 3 | 3 | 6 |
| phchp349v2 | PSYCH | M | 58 | Caucasian | 0 | Low Pain | 4 | 4 | 8 |
| phchp134v4 | BP | M | 61 | Caucasian | 1 | Low Pain | 4 | 4 | 8 |
| phchp153v3 | BP | M | 56 | Caucasian | 1 | Low Pain | 2 | 2 | 4 |
| phchp210v1 | BP | M | 43 | Caucasian | 1 | Low Pain | 2 | 2 | 4 |
| phchp214v1 | PTSD | F | 52 | Caucasian | 1 | Low Pain | 4 | 4 | 8 |
| phchp223v3 | SZA | F | 61 | Caucasian | 1 | Low Pain | 2 | 2 | 4 |
| phchp004v2 | SZA | M | 60 | African American | 2 | Low Pain | 4 | 4 | 8 |
| phchp106v1 | BP | F | 28 | Mixed | 2 | Low Pain | 3 | 3 | 6 |
| phchp164v1 | MDD | F | 48 | Caucasian | 2 | Low Pain | 4 | 4 | 8 |
| phchp192v1 | SZA | M | 55 | African American | 2 | Low Pain | 4 | 4 | 8 |
| phchp221v3 | MDD | M | 52 | African American | 2 | Low Pain | 3 | 3 | 6 |
| phchp223v2 | SZA | F | 60 | Caucasian | 2 | Low Pain | 4 | 4 | 8 |
| phchp243v4 |  | M | 54 | African American | 2 | Low Pain | 3 | 3 | 6 |
| phchp291v1 | SZ | F | 45 | Caucasian | 2 | Low Pain | 3 | 3 | 6 |
| phchp302v1 | BP | M | 61 | Caucasian | 2 | Low Pain | 5 | 5 | 10 |
| phchp323v1 | PTSD | M | 32 | Caucasian | 2 | Low Pain | 4 | 4 | 8 |
| phchp332v2 | SZA | F | 48 | African American | 2 | Low Pain | 4 | 4 | 8 |
| phchp334v3 | BP | F | 51 | Caucasian | 2 | Low Pain | 4 | 4 | 8 |
| phchp341v4 | MDD | M | 46 | Caucasian | 2 | Low Pain | 3 | 3 | 6 |
| phchp341v5 | MDD | M | 46 | Caucasian | 2 | Low Pain | 4 | 4 | 8 |
| phchp106v3 | BP | F | 29 | Mixed | 6 | High Pain | 5 | 5 | 10 |
| phchp140v2 | BP | M | 38 | Caucasian | 6 | High Pain | 4 | 4 | 8 |
| phchp142v2 | BP | M | 55 | Caucasian | 6 | High Pain | 5 | 5 | 10 |
| phchp153v1 | BP | M | 55 | Caucasian | 6 | High Pain | 2 | 2 | 4 |
| phchp164v3 | MDD | F | 49 | Caucasian | 6 | High Pain | 5 | 5 | 10 |
| phchp209v1 | PTSD | M | 54 | African American | 6 | High Pain | 4 | 4 | 8 |
| phchp209v2 | PTSD | M | 54 | African American | 6 | High Pain | 4 | 4 | 8 |
| phchp210v2 | BP | M | 43 | Caucasian | 6 | High Pain | 5 | 5 | 10 |
| phchp221v1 | MDD | M | 51 | African American | 6 | High Pain | 5 | 5 | 10 |
| phchp221v2 | MDD | M | 51 | African American | 6 | High Pain | 4 | 4 | 8 |
| phchp291v2 | SZ | F | 46 | Caucasian | 6 | High Pain | 4 | 4 | 8 |
| phchp291v5 | SZ | F | 48 | Caucasian | 6 | High Pain | 4 | 4 | 8 |
| phchp302v2 | BP | M | 61 | Caucasian | 6 | High Pain | 6 | 6 | 12 |
| phchp334v1 | BP | F | 50 | Caucasian | 6 | High Pain | 4 | 4 | 8 |
| phchp349v1 | PSYCH | M | 58 | Caucasian | 6 | High Pain | 3 | 3 | 6 |
| phchp243v1 | PTSD | M | 50 | African American | 6.5 | High Pain | 5 | 5 | 10 |
| phchp341v1 | MDD | M | 45 | Caucasian | 6.5 | High Pain | 5 | 5 | 10 |
| phchp052v1 | SZ | M | 60 | Caucasian | 7 | High Pain | 2 | 2 | 4 |
| phchp131v1 | SZ | F | 54 | African American | 7 | High Pain | 2 | 2 | 4 |
| phchp134v3 | BP | M | 59 | Caucasian | 7 | High Pain | 6 | 6 | 12 |
| phchp142v6 | BP | M | 58 | Caucasian | 7 | High Pain | 5 | 5 | 10 |
| phchp185v1 | SZA | M | 51 | African American | 7 | High Pain | 6 | 6 | 12 |
| phchp185v3 | SZA | M | 52 | African American | 7 | High Pain | 1 | 1 | 2 |
| phchp192v2 | SZA | M | 56 | African American | 7 | High Pain | 4 | 4 | 8 |
| phchp205v3 | PTSD | F | 54 | Caucasian | 7 | High Pain | 4 | 4 | 8 |
| phchp214v3 | PTSD | F | 57 | Caucasian | 7 | High Pain | 5 | 5 | 10 |
| phchp323v2 | PTSD | M | 33 | Caucasian | 7 | High Pain | 5 | 5 | 10 |
| phchp341v2 | MDD | M | 45 | Caucasian | 7 | High Pain | 5 | 5 | 10 |
| phchp004v4 | SZA | M | 63 | African American | 8 | High Pain | 5 | 5 | 10 |
| phchp136v3 | BP | M | 41 | Caucasian | 8 | High Pain | 6 | 6 | 12 |
| phchp142v1 | BP | M | 55 | Caucasian | 8 | High Pain | 4 | 4 | 8 |
| phchp154v1 | PSYCH | M | 51 | African American | 8 | High Pain | 6 | 6 | 12 |
| phchp191v2 | SZA | M | 58 | African American | 8 | High Pain | 5 | 5 | 10 |
| phchp223v1 | SZA | F | 60 | Caucasian | 8 | High Pain | 5 | 5 | 10 |
| phchp332v3 | SZA | F | 48 | African American | 8 | High Pain | 4 | 4 | 8 |
| phchp341v3 | MDD | M | 45 | Caucasian | 8 | High Pain | 5 | 5 | 10 |
| phchp142v3 | BP | M | 55 | Caucasian | 9 | High Pain | 6 | 6 | 12 |
| phchp205v2 | PTSD | F | 54 | Caucasian | 10 | High Pain | 4 | 4 | 8 |

| **Independent Validation Cohort (n=23) (30 visits)** | | | | | | | | | |
| --- | --- | --- | --- | --- | --- | --- | --- | --- | --- |
| **Participant ID and visit number** | **Diagnosis** | **Gender** | **Age** | **Ethnicity** | **Pain Scale(1-10)** | **Validation cohort** | **SF-36 Pain (Q21)** | **SF-36 Pain (Q22)** | **SF-36 Pain (Q21+ Q22) SEVERE>=10** |
| phchp088v5 | BP | M | 50 | Caucasian | 7 | Severe Clinical Pain | 6 | 5 | 11 |
| phchp121v1 | MOOD | M | 55 | Caucasian | 10 | Severe Clinical Pain | 6 | 5 | 11 |
| phchp133v1 | SZ | M | 55 | Caucasian | 8 | Severe Clinical Pain | 6 | 5 | 11 |
| phchp141v1 | BP | F | 47 | Caucasian | 6 | Severe Clinical Pain | 6 | 4 | 10 |
| phchp141v2 | BP | F | 47 | Caucasian | 7 | Severe Clinical Pain | 6 | 5 | 11 |
| phchp141v3 | BP | F | 47 | Caucasian | 6 | Severe Clinical Pain | 6 | 4 | 10 |
| phchp155v1 | MDD | M | 37 | Caucasian | 10 | Severe Clinical Pain | 6 | 5 | 11 |
| phchp160v3 | SZA | F | 41 | Caucasian | 6 | Severe Clinical Pain | 6 | 4 | 10 |
| phchp182v2 | MDD | M | 39 | Caucasian | 7 | Severe Clinical Pain | 5 | 5 | 10 |
| phchp203v1 | MOOD | M | 59 | African American | 8 | Severe Clinical Pain | 5 | 5 | 10 |
| phchp206v1 | MDD | M | 59 | African American | 7 | Severe Clinical Pain | 6 | 4 | 10 |
| phchp215v1 | PTSD | F | 58 | Caucasian | 7.5 | Severe Clinical Pain | 5 | 5 | 10 |
| phchp254v2 | MDD | F | 49 | Caucasian | 8 | Severe Clinical Pain | 6 | 5 | 11 |
| phchp282v1 | SZ | M | 56 | Caucasian | 7 | Severe Clinical Pain | 5 | 5 | 10 |
| phchp282v2 | SZ | M | 57 | Caucasian | 7 | Severe Clinical Pain | 5 | 5 | 10 |
| phchp285v3 | BP | F | 57 | Caucasian | 10 | Severe Clinical Pain | 6 | 5 | 11 |
| phchp287v1 | SZA | M | 59 | Caucasian | 10 | Severe Clinical Pain | 6 | 4 | 10 |
| phchp287v2 | SZA | M | 60 | Caucasian | 9 | Severe Clinical Pain | 6 | 4 | 10 |
| phchp310v3 | MOOD | M | 54 | African American | 7 | Severe Clinical Pain | 5 | 5 | 10 |
| phchp311v1 | MDD | F | 60 | African American | 8 | Severe Clinical Pain | 5 | 5 | 10 |
| phchp311v2 | MDD | F | 61 | African American | 8 | Severe Clinical Pain | 6 | 5 | 11 |
| phchp336v2 | MDD | M | 61 | African American | 6 | Severe Clinical Pain | 6 | 4 | 10 |
| phchp338v2 | BP | F | 51 | Caucasian | 8 | Severe Clinical Pain | 6 | 5 | 11 |
| phchp338v3 | BP | F | 51 | Caucasian | 7 | Severe Clinical Pain | 6 | 4 | 10 |
| phchp338v4 | BP | F | 52 | Caucasian | 6 | Severe Clinical Pain | 6 | 4 | 10 |
| phchp340v3 | MDD | F | 52 | Caucasian | 7 | Severe Clinical Pain | 5 | 5 | 10 |
| phchp346v1 | PTSD | F | 36 | African American | 7 | Severe Clinical Pain | 6 | 5 | 11 |
| phchp350v1 | BP | M | 48 | Caucasian | 9 | Severe Clinical Pain | 6 | 4 | 10 |
| phchp355v2 | MDD | F | 50 | Caucasian | 7 | Severe Clinical Pain | 6 | 4 | 10 |
| phchp367v1 | BP | M | 48 | Caucasian | 8 | Severe Clinical Pain | 5 | 5 | 10 |

| **Independent Test Cohort (n=218) (565 visits)** | | | | | | | | | | | | |
| --- | --- | --- | --- | --- | --- | --- | --- | --- | --- | --- | --- | --- |
| **Participant ID and visit number** | **Diagnosis** | **Gender** | **Age** | **Ethnicity** | **Pain Scale(1-10)** | **State Test Cohort** | **First Year ED Cohort** | **Number of First Year**  **Future ED Pain visits** | **All Future ED Cohort** | **Number of**  **All Future ED Pain visits** | **Frequency of All Future ED Pain visits/day** | **Length of Future Follow-up**  **(days)** |
| phchp304v2 | MDD | M | 52 | Caucasian | 7 | Yes | Yes | 6 | Yes | 7 | 0.00505 | 1386 |
| phchp198v1 | MDD | M | 61 | Caucasian | 8 | Yes | Yes | 5 | Yes | 6 | 0.00269 | 2227 |
| phchp198v2 | MDD | M | 61 | Caucasian | 8 | Yes | Yes | 5 | Yes | 6 | 0.00281 | 2138 |
| phchp198v4 | MDD | M | 62 | Caucasian | 9 | Yes | Yes | 5 | Yes | 6 | 0.00306 | 1962 |
| phchp304v1 | MDD | M | 52 | Caucasian | 6 | Yes | Yes | 5 | Yes | 7 | 0.00472 | 1484 |
| phchp304v3 | MDD | M | 52 | Caucasian | 6 | Yes | Yes | 5 | Yes | 6 | 0.00489 | 1228 |
| phchp188v3 | PSYCH | M | 54 | African American | 8 | Yes | Yes | 4 | Yes | 6 | 0.0029 | 2070 |
| phchp212v1 | MDD | M | 56 | African American | 7 | Yes | Yes | 4 | Yes | 15 | 0.008 | 1876 |
| phchp365v1 | MDD | M | 59 | African American | 2 | Yes | Yes | 4 | Yes | 7 | 0.0119 | 588 |
| phchp178v1 | BP | M | 49 | Caucasian | 5 | Yes | Yes | 3 | Yes | 5 | 0.00237 | 2108 |
| phchp197v1 | SZ | M | 56 | Caucasian | 4 | Yes | Yes | 3 | Yes | 4 | 0.00178 | 2247 |
| phchp208v3 | MDD | M | 58 | African American | 7 | Yes | Yes | 3 | Yes | 7 | 0.00458 | 1529 |
| phchp212v2 | MDD | M | 56 | African American | 8 | Yes | Yes | 3 | Yes | 13 | 0.00724 | 1796 |
| phchp240v3 | MDD | F | 56 | Caucasian | 4 | Yes | Yes | 3 | Yes | 5 | 0.00346 | 1445 |
| phchp327v1 | MDD | M | 42 | Caucasian | 6 | Yes | Yes | 3 | Yes | 4 | 0.00334 | 1197 |
| phchp357v4 | BP | M | 45 | Caucasian | 1 | Yes | Yes | 3 | Yes | 3 | 0.00633 | 474 |
| phchp187v1 | SZ | M | 49 | African American | 3 | Yes | Yes | 2 | Yes | 12 | 0.00508 | 2364 |
| phchp236v3 | MDD | M | 54 | Caucasian | 0 | Yes | Yes | 2 | Yes | 6 | 0.00512 | 1173 |
| phchp240v2 | MDD | F | 55 | Caucasian | 3 | Yes | Yes | 2 | Yes | 5 | 0.00317 | 1576 |
| phchp242v2 | MDD | M | 57 | African American | 4 | Yes | Yes | 2 | Yes | 3 | 0.00282 | 1063 |
| phchp298v4 | SZA | M | 58 | Caucasian | 1 | Yes | Yes | 2 | Yes | 3 | 0.00487 | 616 |
| phchp342v1 | MDD | M | 52 | Caucasian | 7 | Yes | Yes | 2 | Yes | 3 | 0.00291 | 1030 |
| phchp353v1 | MDD | F | 45 | Caucasian | 3.5 | Yes | Yes | 2 | Yes | 2 | 0.00239 | 836 |
| phchp357v2 | BP | M | 45 | Caucasian | 1 | Yes | Yes | 2 | Yes | 3 | 0.00426 | 705 |
| phchp357v3 | BP | M | 45 | Caucasian | 2 | Yes | Yes | 2 | Yes | 3 | 0.0053 | 566 |
| phchp237v1 | PTSD | M | 39 | Caucasian | 5 | Yes |  | 2 | Yes | 3 | 0.00147 | 2038 |
| phchp237v2 | PTSD | M | 39 | Caucasian | 6 | Yes |  | 2 | Yes | 2 | 0.00104 | 1922 |
| phchp019v5 | SZ | M | 59 | African American | 0 | Yes | Yes | 1 | Yes | 1 | 0.00158 | 634 |
| phchp099v3 | SZ | M | 49 | Caucasian | 0 | Yes | Yes | 1 | Yes | 15 | 0.00588 | 2550 |
| phchp161v1 | MDD | M | 54 | African American | 1 | Yes | Yes | 1 | Yes | 3 | 0.00139 | 2153 |
| phchp161v2 | MDD | M | 54 | African American | 4 | Yes | Yes | 1 | Yes | 3 | 0.00146 | 2055 |
| phchp161v3 | MDD | M | 54 | African American | 4 | Yes | Yes | 1 | Yes | 3 | 0.0015 | 1995 |
| phchp183v1 | BP | M | 48 | Caucasian | 3 | Yes | Yes | 1 | Yes | 13 | 0.00548 | 2374 |
| phchp183v2 | BP | M | 48 | Caucasian | 0 | Yes | Yes | 1 | Yes | 13 | 0.00571 | 2278 |
| phchp187v2 | SZ | M | 49 | African American | 9 | Yes | Yes | 1 | Yes | 11 | 0.0049 | 2245 |
| phchp194v1 | MDD | M | 47 | Caucasian | 1 | Yes | Yes | 1 | Yes | 2 | 0.0009 | 2227 |
| phchp194v2 | MDD | M | 47 | Caucasian | 1 | Yes | Yes | 1 | Yes | 2 | 0.00094 | 2138 |
| phchp194v3 | MDD | M | 47 | Caucasian | 3 | Yes | Yes | 1 | Yes | 2 | 0.00098 | 2046 |
| phchp197v2 | SZ | M | 57 | Caucasian | 1 | Yes | Yes | 1 | Yes | 1 | 0.00053 | 1876 |
| phchp213v3 | PTSD | M | 62 | Caucasian | 0 | Yes | Yes | 1 | Yes | 2 | 0.00135 | 1486 |
| phchp219v1 | BP | M | 61 | Caucasian | 5 | Yes | Yes | 1 | Yes | 2 | 0.00097 | 2052 |
| phchp224v1 | BP | M | 59 | Caucasian | 2 | Yes | Yes | 1 | Yes | 1 | 0.00049 | 2058 |
| phchp229v1 | PTSD | M | 55 | African American | 2 | Yes | Yes | 1 | Yes | 18 | 0.00893 | 2015 |
| phchp238v1 | MDD | M | 62 | Caucasian | 2 | Yes | Yes | 1 | Yes | 2 | 0.00099 | 2028 |
| phchp240v1 | MDD | F | 55 | Caucasian | 5 | Yes | Yes | 1 | Yes | 5 | 0.00278 | 1799 |
| phchp242v3 | MDD | M | 57 | African American | 0 | Yes | Yes | 1 | Yes | 2 | 0.0022 | 908 |
| phchp258v1 | BP | F | 52 | Caucasian | 5 | Yes | Yes | 1 | Yes | 3 | 0.002 | 1501 |
| phchp259v1 | MDD | M | 56 | Caucasian | 1 | Yes | Yes | 1 | Yes | 1 | 0.00142 | 702 |
| phchp259v2 | MDD | M | 57 | Caucasian | 3 | Yes | Yes | 1 | Yes | 1 | 0.00168 | 596 |
| phchp259v3 | MDD | M | 57 | Caucasian | 5 | Yes | Yes | 1 | Yes | 1 | 0.00222 | 451 |
| phchp274v1 | BP | M | 48 | Caucasian | 1 | Yes | Yes | 1 | Yes | 3 | 0.00159 | 1884 |
| phchp274v2 | BP | M | 48 | Caucasian | 2 | Yes | Yes | 1 | Yes | 3 | 0.00167 | 1793 |
| phchp274v3 | BP | M | 48 | Caucasian | 2 | Yes | Yes | 1 | Yes | 3 | 0.00177 | 1697 |
| phchp276v3 | SZ | M | 59 | African American | 0 | Yes | Yes | 1 | Yes | 1 | 0.00067 | 1483 |
| phchp299v1 | PTSD | M | 54 | Caucasian | 7 | Yes | Yes | 1 | Yes | 3 | 0.00186 | 1610 |
| phchp299v2 | PTSD | M | 54 | Caucasian | 6 | Yes | Yes | 1 | Yes | 3 | 0.002 | 1497 |
| phchp299v4 | PTSD | M | 55 | Caucasian | 7 | Yes | Yes | 1 | Yes | 2 | 0.00197 | 1013 |
| phchp313v1 | PTSD | M | 46 | African American | 9 | Yes | Yes | 1 | Yes | 1 | 0.00094 | 1061 |
| phchp313v2 | PTSD | M | 46 | African American | 7 | Yes | Yes | 1 | Yes | 1 | 0.0011 | 907 |
| phchp314v3 | BP | M | 54 | Caucasian | 0 | Yes | Yes | 1 | Yes | 1 | 0.00098 | 1022 |
| phchp319v2 | PTSD | M | 42 | African American | 8 | Yes | Yes | 1 | Yes | 2 | 0.00169 | 1182 |
| phchp319v3 | PTSD | M | 43 | African American | 8 | Yes | Yes | 1 | Yes | 2 | 0.00184 | 1088 |
| phchp319v4 | PTSD | M | 42 | African American | 8 | Yes | Yes | 1 | Yes | 1 | 0.0012 | 831 |
| phchp319v5 | PTSD | M | 44 | African American | 4 | Yes | Yes | 1 | Yes | 1 | 0.00141 | 710 |
| phchp342v2 | MDD | M | 52 | Caucasian | 6 | Yes | Yes | 1 | Yes | 2 | 0.00219 | 915 |
| phchp357v1 | BP | M | 45 | Caucasian | 0 | Yes | Yes | 1 | Yes | 3 | 0.00375 | 799 |
| phchp358v1 | PTSD | M | 52 | Hispanic | 5 | Yes | Yes | 1 | Yes | 2 | 0.00304 | 657 |
| phchp358v2 | PTSD | M | 52 | Hispanic | 0 | Yes | Yes | 1 | Yes | 2 | 0.00356 | 562 |
| phchp358v3 | PTSD | M | 53 | Hispanic | 5 | Yes | Yes | 1 | Yes | 2 | 0.00471 | 425 |
| phchp361v2 | PTSD | F | 60 | African American | 6 | Yes | Yes | 1 | Yes | 1 | 0.00167 | 598 |
| phchp361v3 | PTSD | F | 60 | African American | 4 | Yes | Yes | 1 | Yes | 1 | 0.00211 | 475 |
| phchp319v6 | PTSD | M | 44 | African American | 5 | Yes |  | 1 | Yes | 1 | 0.00171 | 586 |
| phchp003v4 | SZ | M | 57 | African American | 1 | Yes | Yes | 0 | Yes | 0 | 0 | 1117 |
| phchp003v5 | SZ | M | 59 | African American | 4 | Yes | Yes | 0 | Yes | 0 | 0 | 561 |
| phchp070v4 | SZ | M | 56 | African American | 0 | Yes | Yes | 0 | Yes | 0 | 0 | 1813 |
| phchp070v5 | SZ | M | 56 | African American | 0 | Yes | Yes | 0 | Yes | 0 | 0 | 1726 |
| phchp070v6 | SZ | M | 57 | African American | 0 | Yes | Yes | 0 | Yes | 0 | 0 | 1637 |
| phchp079v4 | BP | M | 49 | Caucasian | 0 | Yes | Yes | 0 | Yes | 0 | 0 | 803 |
| phchp079v5 | BP | M | 50 | Caucasian | 0 | Yes | Yes | 0 | Yes | 0 | 0 | 668 |
| phchp079v6 | BP | M | 50 | Caucasian | 0 | Yes | Yes | 0 | Yes | 0 | 0 | 535 |
| phchp084v4 | BP | F | 57 | Caucasian | 4 | Yes | Yes | 0 | Yes | 0 | 0 | 1450 |
| phchp092v3 | BP | M | 46 | African American | 0 | Yes | Yes | 0 | Yes | 0 | 0 | 2813 |
| phchp093v1 | BP | M | 51 | Caucasian | 4 | Yes | Yes | 0 | Yes | 0 | 0 | 2845 |
| phchp093v2 | BP | M | 51 | Caucasian | 6 | Yes | Yes | 0 | Yes | 0 | 0 | 2747 |
| phchp093v3 | BP | M | 52 | Caucasian | 7 | Yes | Yes | 0 | Yes | 0 | 0 | 2658 |
| phchp093v4 | BP | M | 56 | Caucasian | 4 | Yes | Yes | 0 | Yes | 0 | 0 | 859 |
| phchp093v5 | BP | M | 57 | Caucasian | 4 | Yes | Yes | 0 | Yes | 0 | 0 | 633 |
| phchp093v6 | BP | M | 57 | Caucasian | 4 | Yes | Yes | 0 | Yes | 0 | 0 | 486 |
| phchp096v1 | SZ | M | 55 | African American | 8 | Yes | Yes | 0 | Yes | 0 | 0 | 2793 |
| phchp096v3 | SZ | M | 56 | African American | 6 | Yes | Yes | 0 | Yes | 0 | 0 | 2619 |
| phchp096v4 | SZ | M | 58 | African American | 5 | Yes | Yes | 0 | Yes | 0 | 0 | 1912 |
| phchp098v1 | SZ | M | 59 | African American | 0 | Yes | Yes | 0 | Yes | 0 | 0 | 2780 |
| phchp099v1 | SZ | M | 49 | Caucasian | 4 | Yes | Yes | 0 | Yes | 15 | 0.00545 | 2751 |
| phchp099v2 | SZ | M | 49 | Caucasian | 0 | Yes | Yes | 0 | Yes | 15 | 0.00564 | 2661 |
| phchp101v1 | SZA | M | 74 | Caucasian | 8 | Yes | Yes | 0 | Yes | 0 | 0 | 1333 |
| phchp105v1 | SZA | M | 59 | Caucasian | 0 | Yes | Yes | 0 | Yes | 0 | 0 | 1030 |
| phchp108v1 | SZ | M | 42 | Caucasian | 3 | Yes | Yes | 0 | Yes | 0 | 0 | 2617 |
| phchp108v2 | SZ | M | 42 | Caucasian | 2 | Yes | Yes | 0 | Yes | 0 | 0 | 2526 |
| phchp108v3 | SZ | M | 43 | Caucasian | 0 | Yes | Yes | 0 | Yes | 0 | 0 | 2430 |
| phchp112v1 | BP | M | 46 | Caucasian/Native Australian | 0 | Yes | Yes | 0 | Yes | 0 | 0 | 1569 |
| phchp112v2 | BP | M | 46 | Caucasian | 0 | Yes | Yes | 0 | Yes | 0 | 0 | 1480 |
| phchp112v3 | BP | M | 47 | Caucasian | 0 | Yes | Yes | 0 | Yes | 0 | 0 | 1359 |
| phchp114v1 | SZA | M | 54 | African American | 5 | Yes | Yes | 0 | Yes | 2 | 0.00082 | 2433 |
| phchp115v1 | BP | M | 67 | Caucasian | 2 | Yes | Yes | 0 | Yes | 0 | 0 | 2617 |
| phchp115v2 | BP | M | 67 | Caucasian | 1 | Yes | Yes | 0 | Yes | 0 | 0 | 2514 |
| phchp115v3 | BP | M | 68 | Caucasian | 1 | Yes | Yes | 0 | Yes | 0 | 0 | 2413 |
| phchp116v1 | SZA | M | 47 | Caucasian | 7 | Yes | Yes | 0 | Yes | 0 | 0 | 2507 |
| phchp117v1 | BP | M | 43 | Caucasian | 2 | Yes | Yes | 0 | Yes | 2 | 0.00077 | 2589 |
| phchp117v2 | BP | M | 43 | Caucasian | 3 | Yes | Yes | 0 | Yes | 2 | 0.0008 | 2505 |
| phchp117v3 | BP | M | 43 | Caucasian | 5 | Yes | Yes | 0 | Yes | 2 | 0.00083 | 2414 |
| phchp118v1 | SZA | M | 46 | African American | 4 | Yes | Yes | 0 | Yes | 0 | 0 | 2433 |
| phchp118v2 | SZA | M | 47 | African American | 5 | Yes | Yes | 0 | Yes | 0 | 0 | 2239 |
| phchp118v4 | SZA | M | 50 | African American | 6 | Yes | Yes | 0 | Yes | 0 | 0 | 1187 |
| phchp119v2 | SZA | M | 56 | African American | 1 | Yes | Yes | 0 | Yes | 0 | 0 | 2134 |
| phchp119v3 | SZA | M | 56 | African American | 1 | Yes | Yes | 0 | Yes | 0 | 0 | 2042 |
| phchp120v1 | SZ | M | 51 | Caucasian | 6 | Yes | Yes | 0 | Yes | 0 | 0 | 2476 |
| phchp120v2 | SZ | M | 51 | Caucasian | 4 | Yes | Yes | 0 | Yes | 0 | 0 | 2392 |
| phchp120v3 | SZ | M | 51 | Caucasian | 6 | Yes | Yes | 0 | Yes | 0 | 0 | 2301 |
| phchp122v1 | BP | M | 51 | Caucasian | 0 | Yes | Yes | 0 | Yes | 0 | 0 | 2387 |
| phchp122v2 | BP | M | 51 | Caucasian | 4 | Yes | Yes | 0 | Yes | 0 | 0 | 2308 |
| phchp124v1 | BP | M | 53 | Caucasian | 3 | Yes | Yes | 0 | Yes | 0 | 0 | 2489 |
| phchp124v2 | BP | M | 54 | Caucasian | 3 | Yes | Yes | 0 | Yes | 0 | 0 | 2372 |
| phchp127v1 | SZA | F | 58 | Caucasian | 7 | Yes | Yes | 0 | Yes | 0 | 0 | 747 |
| phchp127v2 | SZA | F | 58 | Caucasian | 6 | Yes | Yes | 0 | Yes | 0 | 0 | 626 |
| phchp127v3 | SZA | F | 59 | Caucasian | 5 | Yes | Yes | 0 | Yes | 0 | 0 | 535 |
| phchp128v1 | BP | M | 45 | Caucasian | 4 | Yes | Yes | 0 | Yes | 0 | 0 | 2349 |
| phchp128v2 | BP | M | 45 | Caucasian | 1 | Yes | Yes | 0 | Yes | 0 | 0 | 2246 |
| phchp130v1 | MDD | F | 42 | Caucasian | 2 | Yes | Yes | 0 | Yes | 0 | 0 | 2433 |
| phchp130v2 | MDD | F | 42 | Caucasian | 4 | Yes | Yes | 0 | Yes | 0 | 0 | 2324 |
| phchp130v3 | MDD | F | 42 | Caucasian | 4 | Yes | Yes | 0 | Yes | 0 | 0 | 2231 |
| phchp132v1 | BP | M | 51 | Caucasian | 7 | Yes | Yes | 0 | Yes | 0 | 0 | 2376 |
| phchp132v2 | BP | M | 51 | Caucasian | 7 | Yes | Yes | 0 | Yes | 0 | 0 | 2285 |
| phchp132v3 | BP | M | 52 | Caucasian | 7 | Yes | Yes | 0 | Yes | 0 | 0 | 2173 |
| phchp132v4 | BP | M | 54 | Caucasian | 8 | Yes | Yes | 0 | Yes | 0 | 0 | 1378 |
| phchp132v5 | BP | M | 54 | Caucasian | 8 | Yes | Yes | 0 | Yes | 0 | 0 | 1214 |
| phchp132v6 | BP | M | 55 | Caucasian | 8 | Yes | Yes | 0 | Yes | 0 | 0 | 1111 |
| phchp138v1 | MOOD | M | 59 | African American | 0 | Yes | Yes | 0 | Yes | 1 | 0.00043 | 2299 |
| phchp138v2 | MOOD | M | 59 | African American | 5 | Yes | Yes | 0 | Yes | 1 | 0.00045 | 2198 |
| phchp138v3 | MOOD | M | 59 | African American | 0 | Yes | Yes | 0 | Yes | 1 | 0.00047 | 2124 |
| phchp143v1 | BP | F | 62 | African American | 5 | Yes | Yes | 0 | Yes | 0 | 0 | 1928 |
| phchp143v2 | BP | F | 63 | African American | 8 | Yes | Yes | 0 | Yes | 0 | 0 | 1828 |
| phchp143v3 | BP | F | 63 | African American | 7 | Yes | Yes | 0 | Yes | 0 | 0 | 1739 |
| phchp144v1 | SZ | M | 56 | African American | 0 | Yes | Yes | 0 | Yes | 0 | 0 | 2202 |
| phchp149v1 | MOOD | M | 45 | Caucasian | 0 | Yes | Yes | 0 | Yes | 0 | 0 | 1355 |
| phchp149v2 | MOOD | M | 45 | Caucasian | 0 | Yes | Yes | 0 | Yes | 0 | 0 | 1264 |
| phchp149v3 | MOOD | M | 46 | Caucasian | 1 | Yes | Yes | 0 | Yes | 0 | 0 | 1129 |
| phchp152v1 | BP | M | 45 | Caucasian | 4 | Yes | Yes | 0 | Yes | 0 | 0 | 2287 |
| phchp162v1 | MDD | M | 57 | Caucasian | 5 | Yes | Yes | 0 | Yes | 0 | 0 | 2167 |
| phchp162v2 | MDD | M | 57 | Caucasian | 4 | Yes | Yes | 0 | Yes | 0 | 0 | 2036 |
| phchp162v3 | MDD | M | 57 | Caucasian | 5 | Yes | Yes | 0 | Yes | 0 | 0 | 1931 |
| phchp165v1 | SZ | M | 60 | African American | 0 | Yes | Yes | 0 | Yes | 0 | 0 | 2160 |
| phchp165v2 | SZ | M | 60 | African American | 0 | Yes | Yes | 0 | Yes | 0 | 0 | 2069 |
| phchp165v3 | SZ | M | 61 | African American | 0 | Yes | Yes | 0 | Yes | 0 | 0 | 1978 |
| phchp166v1 | BP | M | 56 | Caucasian | 4 | Yes | Yes | 0 | Yes | 0 | 0 | 2123 |
| phchp166v2 | BP | M | 56 | Caucasian | 0 | Yes | Yes | 0 | Yes | 0 | 0 | 2018 |
| phchp166v3 | BP | M | 56 | Caucasian | 4 | Yes | Yes | 0 | Yes | 0 | 0 | 1922 |
| phchp166v4 | BP | M | 58 | Caucasian | 3 | Yes | Yes | 0 | Yes | 0 | 0 | 1344 |
| phchp166v5 | BP | M | 58 | Caucasian | 3 | Yes | Yes | 0 | Yes | 0 | 0 | 1213 |
| phchp166v6 | BP | M | 59 | Caucasian | 4 | Yes | Yes | 0 | Yes | 0 | 0 | 1040 |
| phchp167v1 | MDD | M | 49 | Caucasian | 8 | Yes | Yes | 0 | Yes | 0 | 0 | 2148 |
| phchp168v1 | MDD | M | 48 | African American | 2 | Yes | Yes | 0 | Yes | 0 | 0 | 2153 |
| phchp168v2 | MDD | M | 48 | African American | 0 | Yes | Yes | 0 | Yes | 0 | 0 | 2062 |
| phchp168v3 | MDD | M | 49 | African American | 0 | Yes | Yes | 0 | Yes | 0 | 0 | 1970 |
| phchp169v1 | SZA | M | 50 | African American | 10 | Yes | Yes | 0 | Yes | 15 | 0.00749 | 2003 |
| phchp173v1 | MDD | M | 48 | Caucasian | 4 | Yes | Yes | 0 | Yes | 0 | 0 | 1890 |
| phchp173v2 | MDD | M | 49 | Caucasian | 3 | Yes | Yes | 0 | Yes | 0 | 0 | 1801 |
| phchp173v3 | MDD | M | 49 | Caucasian | 6 | Yes | Yes | 0 | Yes | 0 | 0 | 1710 |
| phchp174v1 | MDD | M | 54 | Caucasian | 6 | Yes | Yes | 0 | Yes | 0 | 0 | 851 |
| phchp175v1 | SZA | M | 42 | Caucasian | 0 | Yes | Yes | 0 | Yes | 0 | 0 | 2130 |
| phchp184v1 | BP | M | 64 | Caucasian | 4 | Yes | Yes | 0 | Yes | 7 | 0.00298 | 2352 |
| phchp184v2 | BP | M | 64 | Caucasian | 0 | Yes | Yes | 0 | Yes | 7 | 0.0031 | 2259 |
| phchp184v3 | BP | M | 64 | Caucasian | 5 | Yes | Yes | 0 | Yes | 7 | 0.00323 | 2168 |
| phchp186v1 | BP | M | 43 | Caucasian | 2 | Yes | Yes | 0 | Yes | 1 | 0.00043 | 2310 |
| phchp186v2 | BP | M | 44 | Caucasian | 3 | Yes | Yes | 0 | Yes | 1 | 0.00045 | 2206 |
| phchp186v3 | BP | M | 44 | Caucasian | 2 | Yes | Yes | 0 | Yes | 1 | 0.00047 | 2128 |
| phchp186v4 | BP | M | 46 | Caucasian | 3 | Yes | Yes | 0 | Yes | 1 | 0.00076 | 1319 |
| phchp190v1 | BP | M | 49 | Caucasian | 0 | Yes | Yes | 0 | Yes | 0 | 0 | 2344 |
| phchp190v2 | BP | M | 49 | Caucasian | 0 | Yes | Yes | 0 | Yes | 0 | 0 | 2248 |
| phchp190v3 | BP | M | 50 | Caucasian | 0 | Yes | Yes | 0 | Yes | 0 | 0 | 2139 |
| phchp195v1 | SZ | M | 52 | Caucasian | 5 | Yes | Yes | 0 | Yes | 0 | 0 | 1241 |
| phchp195v2 | SZ | M | 53 | Caucasian | 6 | Yes | Yes | 0 | Yes | 0 | 0 | 1150 |
| phchp195v3 | SZ | M | 53 | Caucasian | 4 | Yes | Yes | 0 | Yes | 0 | 0 | 1061 |
| phchp196v1 | MDD | M | 56 | African American | 6 | Yes | Yes | 0 | Yes | 10 | 0.00533 | 1876 |
| phchp196v2 | MDD | M | 56 | African American | 6 | Yes | Yes | 0 | Yes | 10 | 0.0056 | 1785 |
| phchp196v3 | MDD | M | 57 | African American | 5 | Yes | Yes | 0 | Yes | 10 | 0.00598 | 1673 |
| phchp197v3 | SZ | M | 57 | Caucasian | 2 | Yes | Yes | 0 | Yes | 0 | 0 | 1778 |
| phchp197v4 | SZ | M | 58 | Caucasian | 2 | Yes | Yes | 0 | Yes | 0 | 0 | 1641 |
| phchp199v1 | SZ | M | 49 | African American | 0 | Yes | Yes | 0 | Yes | 3 | 0.00133 | 2259 |
| phchp199v2 | SZ | M | 49 | African American | 0 | Yes | Yes | 0 | Yes | 3 | 0.00139 | 2161 |
| phchp199v3 | SZ | M | 50 | African American | 0 | Yes | Yes | 0 | Yes | 3 | 0.00147 | 2047 |
| phchp200v1 | MDD | M | 56 | Caucasian | 2 | Yes | Yes | 0 | Yes | 0 | 0 | 2092 |
| phchp200v2 | MDD | M | 57 | Caucasian | 2 | Yes | Yes | 0 | Yes | 0 | 0 | 2001 |
| phchp200v3 | MDD | M | 57 | Caucasian | 0 | Yes | Yes | 0 | Yes | 0 | 0 | 1904 |
| phchp207v1 | SZ | M | 48 | African American | 6 | Yes | Yes | 0 | Yes | 0 | 0 | 1991 |
| phchp208v1 | MDD | M | 56 | African American | 4 | Yes | Yes | 0 | Yes | 7 | 0.00322 | 2171 |
| phchp208v2 | MDD | M | 56 | African American | 7 | Yes | Yes | 0 | Yes | 7 | 0.00338 | 2074 |
| phchp211v1 | SZ | M | 62 | Caucasian | 0 | Yes | Yes | 0 | Yes | 1 | 0.00047 | 2127 |
| phchp211v2 | SZ | M | 62 | Caucasian | 2 | Yes | Yes | 0 | Yes | 1 | 0.00052 | 1929 |
| phchp211v3 | SZ | M | 62 | Caucasian | 2 | Yes | Yes | 0 | Yes | 1 | 0.00054 | 1835 |
| phchp213v1 | PTSD | M | 62 | Caucasian | 5 | Yes | Yes | 0 | Yes | 2 | 0.00118 | 1690 |
| phchp213v2 | PTSD | M | 62 | Caucasian | 4 | Yes | Yes | 0 | Yes | 2 | 0.00125 | 1596 |
| phchp216v1 | MOOD | M | 50 | African American | 8 | Yes | Yes | 0 | Yes | 6 | 0.00287 | 2094 |
| phchp216v2 | MOOD | M | 51 | African American | 9 | Yes | Yes | 0 | Yes | 6 | 0.00305 | 1969 |
| phchp216v3 | MOOD | M | 51 | African American | 8 | Yes | Yes | 0 | Yes | 6 | 0.0032 | 1877 |
| phchp219v2 | BP | M | 61 | Caucasian | 1 | Yes | Yes | 0 | Yes | 1 | 0.00051 | 1943 |
| phchp219v3 | BP | M | 62 | Caucasian | 1 | Yes | Yes | 0 | Yes | 1 | 0.00057 | 1742 |
| phchp222v2 | SZ | M | 60 | Caucasian | 4 | Yes | Yes | 0 | Yes | 3 | 0.00166 | 1810 |
| phchp222v3 | SZ | M | 61 | Caucasian | 3 | Yes | Yes | 0 | Yes | 3 | 0.00174 | 1721 |
| phchp227v1 | MDD | M | 55 | Caucasian | 3 | Yes | Yes | 0 | Yes | 1 | 0.00049 | 2046 |
| phchp227v2 | MDD | M | 55 | Caucasian | 4 | Yes | Yes | 0 | Yes | 1 | 0.00051 | 1959 |
| phchp227v3 | MDD | M | 55 | Caucasian | 5 | Yes | Yes | 0 | Yes | 1 | 0.00054 | 1856 |
| phchp228v1 | PTSD | M | 43 | African American | 4 | Yes | Yes | 0 | Yes | 0 | 0 | 2057 |
| phchp234v1 | BP | M | 44 | Caucasian | 2 | Yes | Yes | 0 | Yes | 5 | 0.00276 | 1814 |
| phchp234v2 | BP | M | 45 | Caucasian | 2 | Yes | Yes | 0 | Yes | 5 | 0.00312 | 1604 |
| phchp234v3 | BP | M | 45 | Caucasian | 1 | Yes | Yes | 0 | Yes | 5 | 0.0033 | 1513 |
| phchp235v1 | MDD | M | 54 | African American | 7 | Yes | Yes | 0 | Yes | 0 | 0 | 2040 |
| phchp235v2 | MDD | M | 55 | African American | 3 | Yes | Yes | 0 | Yes | 0 | 0 | 1948 |
| phchp235v3 | MDD | M | 55 | African American | 3 | Yes | Yes | 0 | Yes | 0 | 0 | 1829 |
| phchp236v1 | MDD | M | 51 | Caucasian | 1 | Yes | Yes | 0 | Yes | 11 | 0.00542 | 2031 |
| phchp236v2 | MDD | M | 51 | Caucasian | 1 | Yes | Yes | 0 | Yes | 11 | 0.00567 | 1939 |
| phchp238v2 | MDD | M | 63 | Caucasian | 2 | Yes | Yes | 0 | Yes | 1 | 0.00052 | 1938 |
| phchp238v3 | MDD | M | 63 | Caucasian | 4 | Yes | Yes | 0 | Yes | 1 | 0.00055 | 1818 |
| phchp242v1 | MDD | M | 55 | African American | 3 | Yes | Yes | 0 | Yes | 3 | 0.00157 | 1912 |
| phchp247v1 | MDD | M | 55 | African American | 8 | Yes | Yes | 0 | Yes | 1 | 0.00049 | 2024 |
| phchp248v1 | SZ | M | 52 | African American | 0 | Yes | Yes | 0 | Yes | 0 | 0 | 1516 |
| phchp248v2 | SZ | M | 52 | African American | 0 | Yes | Yes | 0 | Yes | 0 | 0 | 1417 |
| phchp248v3 | SZ | M | 53 | African American | 2 | Yes | Yes | 0 | Yes | 0 | 0 | 1325 |
| phchp258v2 | BP | F | 52 | Caucasian | 8 | Yes | Yes | 0 | Yes | 2 | 0.00156 | 1278 |
| phchp258v3 | BP | F | 54 | Caucasian | 5 | Yes | Yes | 0 | Yes | 0 | 0 | 618 |
| phchp265v1 | PTSD | M | 43 | Caucasian | 2 | Yes | Yes | 0 | Yes | 0 | 0 | 1478 |
| phchp266v1 | MOOD | M | 41 | Caucasian | 3 | Yes | Yes | 0 | Yes | 0 | 0 | 1977 |
| phchp266v2 | MOOD | M | 42 | Caucasian | 5 | Yes | Yes | 0 | Yes | 0 | 0 | 1876 |
| phchp266v3 | MOOD | M | 42 | Caucasian | 4 | Yes | Yes | 0 | Yes | 0 | 0 | 1780 |
| phchp270v3 | BP | M | 41 | Caucasian | 1 | Yes | Yes | 0 | Yes | 0 | 0 | 1586 |
| phchp270v4 | BP | M | 41 | Caucasian | 2 | Yes | Yes | 0 | Yes | 0 | 0 | 1476 |
| phchp270v5 | BP | M | 42 | Caucasian | 3.5 | Yes | Yes | 0 | Yes | 0 | 0 | 1259 |
| phchp270v6 | BP | M | 44 | Caucasian | 3 | Yes | Yes | 0 | Yes | 0 | 0 | 539 |
| phchp274v4 | BP | M | 50 | Caucasian | 1 | Yes | Yes | 0 | Yes | 2 | 0.00239 | 836 |
| phchp275v1 | SZ | M | 63 | Caucasian | 0 | Yes | Yes | 0 | Yes | 0 | 0 | 1472 |
| phchp275v2 | SZ | M | 63 | Caucasian | 0 | Yes | Yes | 0 | Yes | 0 | 0 | 1363 |
| phchp275v3 | SZ | M | 63 | Caucasian | 1 | Yes | Yes | 0 | Yes | 0 | 0 | 1246 |
| phchp276v1 | SZ | M | 59 | African American | 0 | Yes | Yes | 0 | Yes | 1 | 0.0006 | 1662 |
| phchp276v2 | SZ | M | 59 | African American | 0 | Yes | Yes | 0 | Yes | 1 | 0.00064 | 1568 |
| phchp276v4 | SZ | M | 61 | African American | 0 | Yes | Yes | 0 | Yes | 0 | 0 | 777 |
| phchp277v1 | SZ | M | 49 | Caucasian | 5 | Yes | Yes | 0 | Yes | 0 | 0 | 1876 |
| phchp277v2 | SZ | M | 50 | Caucasian | 4 | Yes | Yes | 0 | Yes | 0 | 0 | 1783 |
| phchp277v3 | SZ | M | 50 | Caucasian | 4 | Yes | Yes | 0 | Yes | 0 | 0 | 1690 |
| phchp277v4 | SZ | M | 52 | Caucasian | 4 | Yes | Yes | 0 | Yes | 0 | 0 | 836 |
| phchp277v5 | SZ | M | 52 | Caucasian | 5 | Yes | Yes | 0 | Yes | 0 | 0 | 731 |
| phchp279v1 | SZ | M | 60 | African American | 6 | Yes | Yes | 0 | Yes | 3 | 0.00259 | 1160 |
| phchp279v2 | SZ | M | 61 | African American | 8 | Yes | Yes | 0 | Yes | 3 | 0.00281 | 1068 |
| phchp279v3 | SZ | M | 61 | African American | 6 | Yes | Yes | 0 | Yes | 3 | 0.00311 | 965 |
| phchp279v4 | SZ | M | 61 | African American | 7 | Yes | Yes | 0 | Yes | 3 | 0.00371 | 809 |
| phchp283v1 | SZ | M | 51 | Caucasian | 2 | Yes | Yes | 0 | Yes | 0 | 0 | 1666 |
| phchp289v1 | PTSD | F | 50 | Caucasian | 2 | Yes | Yes | 0 | Yes | 5 | 0.00276 | 1810 |
| phchp290v1 | BP | M | 55 | Caucasian | 5 | Yes | Yes | 0 | Yes | 3 | 0.00187 | 1608 |
| phchp290v2 | BP | M | 55 | Caucasian | 4 | Yes | Yes | 0 | Yes | 3 | 0.00204 | 1473 |
| phchp290v3 | BP | M | 55 | Caucasian | 4 | Yes | Yes | 0 | Yes | 3 | 0.00218 | 1376 |
| phchp292v1 | BP | M | 42 | Caucasian | 0 | Yes | Yes | 0 | Yes | 1 | 0.00058 | 1736 |
| phchp292v2 | BP | M | 42 | Caucasian | 3 | Yes | Yes | 0 | Yes | 1 | 0.00061 | 1645 |
| phchp292v3 | BP | M | 42 | Caucasian | 4 | Yes | Yes | 0 | Yes | 1 | 0.00067 | 1499 |
| phchp293v1 | BP | M | 43 | Caucasian | 4 | Yes | Yes | 0 | Yes | 0 | 0 | 1746 |
| phchp293v2 | BP | M | 44 | Caucasian | 0 | Yes | Yes | 0 | Yes | 0 | 0 | 1644 |
| phchp295v1 | SZ | M | 52 | African American | 0 | Yes | Yes | 0 | Yes | 0 | 0 | 375 |
| phchp296v1 | BP | M | 48 | Caucasian | 1 | Yes | Yes | 0 | Yes | 0 | 0 | 1634 |
| phchp296v2 | BP | M | 49 | Caucasian | 2 | Yes | Yes | 0 | Yes | 0 | 0 | 1366 |
| phchp296v3 | BP | M | 50 | Caucasian | 2 | Yes | Yes | 0 | Yes | 0 | 0 | 892 |
| phchp297v1 | SZA | M | 54 | African American | 0 | Yes | Yes | 0 | Yes | 0 | 0 | 1422 |
| phchp297v2 | SZA | M | 55 | African American | 0 | Yes | Yes | 0 | Yes | 0 | 0 | 1314 |
| phchp297v3 | SZA | M | 55 | African American | 0 | Yes | Yes | 0 | Yes | 0 | 0 | 1204 |
| phchp297v4 | SZA | M | 57 | African American | 0 | Yes | Yes | 0 | Yes | 0 | 0 | 590 |
| phchp298v1 | SZA | M | 56 | Caucasian | 1 | Yes | Yes | 0 | Yes | 3 | 0.00213 | 1410 |
| phchp298v2 | SZA | M | 56 | Caucasian | 2 | Yes | Yes | 0 | Yes | 3 | 0.0023 | 1305 |
| phchp298v3 | SZA | M | 56 | Caucasian | 2 | Yes | Yes | 0 | Yes | 3 | 0.00249 | 1206 |
| phchp299v3 | PTSD | M | 54 | Caucasian | 6 | Yes | Yes | 0 | Yes | 2 | 0.00147 | 1361 |
| phchp299v5 | PTSD | M | 56 | Caucasian | 7 | Yes | Yes | 0 | Yes | 1 | 0.00115 | 873 |
| phchp300v1 | BP | M | 47 | Caucasian | 4 | Yes | Yes | 0 | Yes | 0 | 0 | 1393 |
| phchp300v2 | BP | M | 47 | Caucasian | 5 | Yes | Yes | 0 | Yes | 0 | 0 | 1300 |
| phchp300v3 | BP | M | 48 | Caucasian | 3 | Yes | Yes | 0 | Yes | 0 | 0 | 1111 |
| phchp300v4 | BP | M | 49 | Caucasian | 2 | Yes | Yes | 0 | Yes | 0 | 0 | 630 |
| phchp307v1 | PTSD | F | 53 | Caucasian | 8 | Yes | Yes | 0 | Yes | 0 | 0 | 994 |
| phchp308v1 | SZA | M | 47 | African American | 0 | Yes | Yes | 0 | Yes | 2 | 0.00139 | 1437 |
| phchp308v2 | SZA | M | 47 | African American | 0 | Yes | Yes | 0 | Yes | 2 | 0.00167 | 1200 |
| phchp308v3 | SZA | M | 48 | African American | 0 | Yes | Yes | 0 | Yes | 2 | 0.00238 | 842 |
| phchp312v1 | BP | M | 64 | Caucasian | 0 | Yes | Yes | 0 | Yes | 0 | 0 | 1398 |
| phchp312v2 | BP | M | 65 | Caucasian | 1 | Yes | Yes | 0 | Yes | 0 | 0 | 1258 |
| phchp312v3 | BP | M | 65 | Caucasian | 1 | Yes | Yes | 0 | Yes | 0 | 0 | 1163 |
| phchp313v3 | PTSD | M | 46 | African American | 8 | Yes | Yes | 0 | Yes | 1 | 0.0013 | 767 |
| phchp314v1 | BP | M | 54 | Caucasian | 2 | Yes | Yes | 0 | Yes | 1 | 0.00082 | 1217 |
| phchp314v2 | BP | M | 54 | Caucasian | 2 | Yes | Yes | 0 | Yes | 1 | 0.0009 | 1113 |
| phchp315v1 | MDD | M | 62 | Caucasian | 4 | Yes | Yes | 0 | Yes | 1 | 0.00076 | 1321 |
| phchp316v1 | BP | M | 50 | Caucasian | 4 | Yes | Yes | 0 | Yes | 0 | 0 | 1140 |
| phchp316v2 | BP | M | 50 | Caucasian | 3 | Yes | Yes | 0 | Yes | 0 | 0 | 987 |
| phchp316v3 | BP | M | 51 | Caucasian | 3 | Yes | Yes | 0 | Yes | 0 | 0 | 698 |
| phchp316v4 | BP | M | 51 | Caucasian | 3 | Yes | Yes | 0 | Yes | 0 | 0 | 683 |
| phchp316v5 | BP | M | 51 | Caucasian | 6 | Yes | Yes | 0 | Yes | 0 | 0 | 452 |
| phchp318v1 | MDD | F | 57 | Caucasian | 3 | Yes | Yes | 0 | Yes | 0 | 0 | 1677 |
| phchp318v2 | MDD | F | 57 | Caucasian | 2 | Yes | Yes | 0 | Yes | 0 | 0 | 1053 |
| phchp318v3 | MDD | F | 58 | Caucasian | 2 | Yes | Yes | 0 | Yes | 0 | 0 | 858 |
| phchp319v1 | PTSD | M | 42 | African American | 4 | Yes | Yes | 0 | Yes | 2 | 0.00154 | 1301 |
| phchp320v1 | BP | M | 58 | African American | 6 | Yes | Yes | 0 | Yes | 0 | 0 | 1305 |
| phchp320v2 | BP | M | 58 | African American | 6 | Yes | Yes | 0 | Yes | 0 | 0 | 1161 |
| phchp320v3 | BP | M | 59 | African American | 7 | Yes | Yes | 0 | Yes | 0 | 0 | 1068 |
| phchp325v1 | PTSD | M | 44 | Caucasian | 4 | Yes | Yes | 0 | Yes | 1 | 0.00077 | 1298 |
| phchp325v2 | PTSD | M | 44 | Caucasian | 3 | Yes | Yes | 0 | Yes | 1 | 0.00086 | 1165 |
| phchp325v3 | PTSD | M | 44 | Caucasian | 4 | Yes | Yes | 0 | Yes | 1 | 0.00095 | 1053 |
| phchp329v2 | SZA | M | 50 | African American | 8 | Yes | Yes | 0 | Yes | 0 | 0 | 1160 |
| phchp329v3 | SZA | M | 51 | African American | 7 | Yes | Yes | 0 | Yes | 0 | 0 | 1065 |
| phchp330v1 | BP | F | 45 | Caucasian | 4 | Yes | Yes | 0 | Yes | 0 | 0 | 1215 |
| phchp342v3 | MDD | M | 52 | Caucasian | 4 | Yes | Yes | 0 | Yes | 1 | 0.00126 | 796 |
| phchp343v1 | MDD | M | 52 | Caucasian | 4 | Yes | Yes | 0 | Yes | 0 | 0 | 917 |
| phchp343v2 | MDD | M | 52 | Caucasian | 4 | Yes | Yes | 0 | Yes | 0 | 0 | 825 |
| phchp343v3 | MDD | M | 53 | Caucasian | 7 | Yes | Yes | 0 | Yes | 0 | 0 | 659 |
| phchp348v1 | BP | M | 52 | Caucasian | 5 | Yes | Yes | 0 | Yes | 0 | 0 | 904 |
| phchp351v1 | MDD | M | 44 | Caucasian | 0 | Yes | Yes | 0 | Yes | 0 | 0 | 874 |
| phchp351v2 | MDD | M | 44 | Caucasian | 1 | Yes | Yes | 0 | Yes | 0 | 0 | 776 |
| phchp359v1 | PTSD | F | 56 | Caucasian | 7 | Yes | Yes | 0 | Yes | 0 | 0 | 780 |
| phchp359v2 | PTSD | F | 56 | Caucasian | 6 | Yes | Yes | 0 | Yes | 0 | 0 | 677 |
| phchp359v3 | PTSD | F | 57 | Caucasian | 6 | Yes | Yes | 0 | Yes | 0 | 0 | 528 |
| phchp360v1 | BP | F | 56 | Caucasian | 7 | Yes | Yes | 0 | Yes | 0 | 0 | 708 |
| phchp360v3 | BP | F | 57 | Caucasian | 3 | Yes | Yes | 0 | Yes | 0 | 0 | 459 |
| phchp361v1 | PTSD | F | 59 | African American | 4 | Yes | Yes | 0 | Yes | 1 | 0.00138 | 723 |
| phchp362v1 | MDD | M | 54 | Caucasian | 3 | Yes | Yes | 0 | Yes | 0 | 0 | 713 |
| phchp362v2 | MDD | M | 54 | Caucasian | 4 | Yes | Yes | 0 | Yes | 0 | 0 | 602 |
| phchp363v1 | MDD | M | 46 | African American | 1 | Yes | Yes | 0 | Yes | 0 | 0 | 606 |
| phchp366v1 | SZ | M | 56 | Caucasian | 2 | Yes | Yes | 0 | Yes | 0 | 0 | 549 |
| phchp368v1 | MDD | F | 43 | Caucasian | 6 | Yes | Yes | 0 | Yes | 0 | 0 | 504 |
| phchp177v1 | SZ | F | 39 | Caucasian | 0 | Yes |  | 0 | Yes | 0 | 0 | 1718 |
| phchp177v2 | SZ | F | 39 | Caucasian | 0 | Yes |  | 0 | Yes | 0 | 0 | 1609 |
| phchp186v5 | BP | M | 48 | Caucasian | 1 | Yes |  | 0 | Yes | 1 | 0.00158 | 632 |
| phchp190v4 | BP | M | 54 | Caucasian | 0 | Yes |  | 0 | Yes | 0 | 0 | 497 |
| phchp193v3 | BP | M | 39 | Hispanic | 0 | Yes |  | 0 | Yes | 0 | 0 | 2094 |
| phchp193v4 | BP | M | 40 | Hispanic | 0 | Yes |  | 0 | Yes | 0 | 0 | 2001 |
| phchp226v3 | MDD | M | 30 | Caucasian | 1 | Yes |  | 0 | Yes | 0 | 0 | 1464 |
| phchp248v5 | SZ | M | 55 | African American | 1 | Yes |  | 0 | Yes | 0 | 0 | 294 |
| phchp300v5 | BP | M | 50 | Caucasian | 3 | Yes |  | 0 | Yes | 0 | 0 | 313 |
| phchp300v6 | BP | M | 50 | Caucasian | 3 | Yes |  | 0 | Yes | 0 | 0 | 236 |
| phchp308v4 | SZA | M | 49 | African American | 4 | Yes |  | 0 | Yes | 1 | 0.00253 | 395 |
| phchp316v6 | BP | M | 52 | Caucasian | 3 | Yes |  | 0 | Yes | 0 | 0 | 361 |
| phchp325v4 | PTSD | M | 46 | Caucasian | 4 | Yes |  | 0 | Yes | 0 | 0 | 542 |
| phchp333v3 | PTSD | M | 39 | Caucasian | 2 | Yes |  | 0 | Yes | 0 | 0 | 660 |
| phchp333v4 | PTSD | M | 39 | Caucasian | 1 | Yes |  | 0 | Yes | 0 | 0 | 532 |
| phchp356v1 | BP | M | 40 | Caucasian | 3 | Yes |  | 0 | Yes | 0 | 0 | 812 |
| phchp357v5 | BP | M | 46 | Caucasian | 0 | Yes |  | 0 | Yes | 1 | 0.00313 | 320 |
| phchp362v3 | MDD | M | 55 | Caucasian | 4 | Yes |  | 0 | Yes | 0 | 0 | 180 |
| phchp363v2 | MDD | M | 48 | African American | 1 | Yes |  | 0 | Yes | 0 | 0 | 220 |
| phchp368v2 | MDD | F | 44 | Caucasian | 6 | Yes |  | 0 | Yes | 0 | 0 | 161 |
| phchp089v4 | SZA | M | 38 | Caucasian | 5 | Yes |  |  |  |  |  |  |
| phchp097v1 | SZA | F | 25 | Caucasian | 3 | Yes |  |  |  |  |  |  |
| phchp097v2 | SZA | F | 26 | Caucasian | 5 | Yes |  |  |  |  |  |  |
| phchp097v3 | SZA | F | 26 | Caucasian | 7 | Yes |  |  |  |  |  |  |
| phchp102v1 | SZA | M | 56 | Caucasian | 0 | Yes |  |  |  |  |  |  |
| phchp102v2 | SZA | M | 56 | Caucasian | 5 | Yes |  |  |  |  |  |  |
| phchp102v3 | SZA | M | 56 | Caucasian | 1 | Yes |  |  |  |  |  |  |
| phchp113v1 | BP | M | 37 | Caucasian | 7 | Yes |  |  |  |  |  |  |
| phchp150v1 | SZA | M | 61 | Caucasian | 7 | Yes |  |  |  |  |  |  |
| phchp150v2 | SZA | M | 61 | Caucasian | 4 | Yes |  |  |  |  |  |  |
| phchp150v3 | SZA | M | 62 | Caucasian | 7 | Yes |  |  |  |  |  |  |
| phchp156v1 | BP | F | 35 | Caucasian | 4 | Yes |  |  |  |  |  |  |
| phchp157v1 | BP | M | 57 | African American | 7 | Yes |  |  |  |  |  |  |
| phchp157v2 | BP | M | 57 | African American | 6 | Yes |  |  |  |  |  |  |
| phchp157v3 | BP | M | 58 | African American | 7 | Yes |  |  |  |  |  |  |
| phchp170v1 | MDD | F | 26 | Caucasian | 1 | Yes |  |  |  |  |  |  |
| phchp170v2 | MDD | F | 26 | Caucasian | 2 | Yes |  |  |  |  |  |  |
| phchp170v3 | MDD | F | 26 | Caucasian | 2 | Yes |  |  |  |  |  |  |
| phchp172v1 | BP | F | 24 | Caucasian | 2 | Yes |  |  |  |  |  |  |
| phchp172v3 | BP | F | 25 | Caucasian | 2 | Yes |  |  |  |  |  |  |
| phchp180v1 | BP | F | 47 | Caucasian | 0 | Yes |  |  |  |  |  |  |
| phchp180v2 | BP | F | 47 | Caucasian | 0 | Yes |  |  |  |  |  |  |
| phchp180v3 | BP | F | 47 | Caucasian | 0 | Yes |  |  |  |  |  |  |
| phchp181v1 | BP | F | 28 | Caucasian | 5 | Yes |  |  |  |  |  |  |
| phchp181v3 | BP | F | 28 | Caucasian | 1 | Yes |  |  |  |  |  |  |
| phchp204v1 | BP | F | 49 | Caucasian | 0 | Yes |  |  |  |  |  |  |
| phchp204v2 | BP | F | 49 | Caucasian | 0 | Yes |  |  |  |  |  |  |
| phchp204v3 | BP | F | 49 | Caucasian | 3 | Yes |  |  |  |  |  |  |
| phchp217v1 | PTSD | M | 36 | African American | 0 | Yes |  |  |  |  |  |  |
| phchp225v1 | PSYCH | M | 58 | African American | 1 | Yes |  |  |  |  |  |  |
| phchp231v1 | MDD | M | 55 | Caucasian | 7 | Yes |  |  |  |  |  |  |
| phchp232v1 | SZA | F | 38 | Caucasian | 6 | Yes |  |  |  |  |  |  |
| phchp232v2 | SZA | F | 38 | Caucasian | 6 | Yes |  |  |  |  |  |  |
| phchp232v3 | SZA | F | 38 | Caucasian | 3 | Yes |  |  |  |  |  |  |
| phchp239v1 | SZA | F | 54 | African American | 2 | Yes |  |  |  |  |  |  |
| phchp239v2 | SZA | F | 54 | African American | 5 | Yes |  |  |  |  |  |  |
| phchp239v3 | SZA | F | 54 | African American | 1 | Yes |  |  |  |  |  |  |
| phchp241v1 | BP | M | 52 | Caucasian | 8 | Yes |  |  |  |  |  |  |
| phchp253v1 | BP | M | 25 | Caucasian | 6 | Yes |  |  |  |  |  |  |
| phchp253v2 | BP | M | 26 | Caucasian | 8 | Yes |  |  |  |  |  |  |
| phchp253v3 | BP | M | 26 | Caucasian | 6 | Yes |  |  |  |  |  |  |
| phchp273v1 | BP | M | 27 | Caucasian | 6 | Yes |  |  |  |  |  |  |
| phchp273v2 | BP | M | 28 | Caucasian | 6 | Yes |  |  |  |  |  |  |
| phchp286v1 | BP | M | 54 | Caucasian | 6 | Yes |  |  |  |  |  |  |
| phchp286v2 | BP | M | 54 | Caucasian | 7 | Yes |  |  |  |  |  |  |
| phchp286v3 | BP | M | 55 | Caucasian | 3 | Yes |  |  |  |  |  |  |
| phchp309v1 | PTSD | F | 27 | Caucasian | 4 | Yes |  |  |  |  |  |  |
| phchp309v2 | PTSD | F | 28 | Caucasian | 4 | Yes |  |  |  |  |  |  |
| phchp309v3 | PTSD | F | 28 | Caucasian | 1 | Yes |  |  |  |  |  |  |
| phchp324v1 | MDD | M | 33 | African American | 0 | Yes |  |  |  |  |  |  |
| phchp328v1 | MDD | F | 37 | Caucasian | 5 | Yes |  |  |  |  |  |  |
| phchp328v2 | MDD | F | 38 | Caucasian | 5 | Yes |  |  |  |  |  |  |
| phchp328v3 | MDD | F | 38 | Caucasian | 0 | Yes |  |  |  |  |  |  |
| phchp331v1 | BP | M | 53 | Caucasian | 1 | Yes |  |  |  |  |  |  |
| phchp333v1 | PTSD | M | 38 | Caucasian | 0 | Yes |  |  |  |  |  |  |
| phchp333v2 | PTSD | M | 38 | Caucasian | 0 | Yes |  |  |  |  |  |  |
| phchp337v1 | PTSD | M | 34 | Caucasian | 0 | Yes |  |  |  |  |  |  |
| phchp337v2 | PTSD | M | 34 | Caucasian | 2 | Yes |  |  |  |  |  |  |
| phchp337v3 | PTSD | M | 35 | Caucasian | 0 | Yes |  |  |  |  |  |  |
| phchp345v1 | PTSD | M | 33 | Caucasian | 6 | Yes |  |  |  |  |  |  |
| phchp352v1 | MDD | M | 24 | African American | 6 | Yes |  |  |  |  |  |  |
| phchp352v2 | MDD | M | 24 | African American | 6 | Yes |  |  |  |  |  |  |
| phchp352v3 | MDD | M | 25 | African American | 7 | Yes |  |  |  |  |  |  |
| phchp364v1 | PTSD | F | 40 | Caucasian | 0 | Yes |  |  |  |  |  |  |
| phchp021v2 | SZA | M | 49 | Hispanic |  |  | Yes | 5 | Yes | 35 | 0.00919 | 3808 |
| phchp021v3 | SZA | M | 49 | Hispanic |  |  | Yes | 5 | Yes | 35 | 0.00947 | 3694 |
| phchp024v1 | SZA | M | 49 | African American |  |  | Yes | 3 | Yes | 12 | 0.00309 | 3880 |
| phchp068v1 | SZA | M | 57 | African American |  |  | Yes | 3 | Yes | 10 | 0.00287 | 3481 |
| phchp068v2 | SZA | M | 57 | African American |  |  | Yes | 3 | Yes | 9 | 0.00269 | 3350 |
| phchp075v3 | SZA | M | 58 | Caucasian |  |  | Yes | 3 | Yes | 17 | 0.00539 | 3154 |
| phchp021v1 | SZA | M | 48 | Hispanic |  |  | Yes | 2 | Yes | 35 | 0.00899 | 3895 |
| phchp027v1 | SZA | M | 40 | Caucasian |  |  | Yes | 2 | Yes | 6 | 0.00156 | 3858 |
| phchp030v1 | BP | M | 49 | Caucasian |  |  | Yes | 2 | Yes | 5 | 0.0013 | 3846 |
| phchp040v3 | SZA | M | 50 | Caucasian |  |  | Yes | 2 | Yes | 3 | 0.00156 | 1921 |
| phchp046v3 | SZA | M | 45 | Caucasian |  |  | Yes | 2 | Yes | 6 | 0.00172 | 3491 |
| phchp068v3 | SZA | M | 57 | African American |  |  | Yes | 2 | Yes | 8 | 0.00246 | 3255 |
| phchp075v1 | SZA | M | 57 | Caucasian |  |  | Yes | 2 | Yes | 17 | 0.00511 | 3324 |
| phchp075v2 | SZA | M | 58 | Caucasian |  |  | Yes | 2 | Yes | 17 | 0.00526 | 3231 |
| phchp188v1 | PSYCH | M | 48 | African American |  |  | Yes | 2 | Yes | 18 | 0.00459 | 3924 |
| phchp188v2 | PSYCH | M | 49 | African American |  |  | Yes | 2 | Yes | 18 | 0.00475 | 3791 |
| phchp005v1 | SZA | M | 45 | Caucasian |  |  | Yes | 1 | Yes | 4 | 0.00099 | 4040 |
| phchp014v1 | PSYCH | M | 55 | African American |  |  | Yes | 1 | Yes | 8 | 0.00202 | 3958 |
| phchp016v1 | SZ | M | 54 | African American |  |  | Yes | 1 | Yes | 2 | 0.00099 | 2022 |
| phchp030v3 | BP | M | 49 | Caucasian |  |  | Yes | 1 | Yes | 3 | 0.00083 | 3618 |
| phchp039v1 | BP | M | 52 | Caucasian |  |  | Yes | 1 | Yes | 8 | 0.0021 | 3801 |
| phchp039v3 | BP | M | 52 | Caucasian |  |  | Yes | 1 | Yes | 7 | 0.00196 | 3563 |
| phchp040v1 | SZA | M | 50 | Caucasian |  |  | Yes | 1 | Yes | 3 | 0.00142 | 2110 |
| phchp040v2 | SZA | M | 50 | Caucasian |  |  | Yes | 1 | Yes | 3 | 0.00148 | 2026 |
| phchp046v1 | SZA | M | 45 | Caucasian |  |  | Yes | 1 | Yes | 7 | 0.00192 | 3654 |
| phchp046v2 | SZA | M | 45 | Caucasian |  |  | Yes | 1 | Yes | 7 | 0.00195 | 3582 |
| phchp051v1 | SZA | M | 52 | Caucasian |  |  | Yes | 1 | Yes | 3 | 0.00082 | 3659 |
| phchp069v1 | SZ | M | 47 | Caucasian |  |  | Yes | 1 | Yes | 4 | 0.00114 | 3523 |
| phchp069v2 | SZ | M | 47 | Caucasian |  |  | Yes | 1 | Yes | 4 | 0.00117 | 3414 |
| phchp073v1 | SZA | M | 50 | Caucasian |  |  | Yes | 1 | Yes | 2 | 0.00057 | 3486 |
| phchp073v2 | SZA | M | 50 | Caucasian |  |  | Yes | 1 | Yes | 2 | 0.0006 | 3360 |
| phchp073v3 | SZA | M | 50 | Caucasian |  |  | Yes | 1 | Yes | 2 | 0.00061 | 3271 |
| phchp080v1 | BP | M | 44 | Caucasian |  |  | Yes | 1 | Yes | 5 | 0.00153 | 3274 |
| phchp003v1 | SZ | M | 50 | African American |  |  | Yes | 0 | Yes | 0 | 0 | 4115 |
| phchp003v2 | SZ | M | 50 | African American |  |  | Yes | 0 | Yes | 0 | 0 | 3947 |
| phchp003v3 | SZ | M | 50 | African American |  |  | Yes | 0 | Yes | 0 | 0 | 3856 |
| phchp005v2 | SZA | M | 45 | Caucasian |  |  | Yes | 0 | Yes | 3 | 0.00077 | 3909 |
| phchp005v3 | SZA | M | 45 | Caucasian |  |  | Yes | 0 | Yes | 3 | 0.00079 | 3814 |
| phchp006v1 | SZA | M | 52 | African American |  |  | Yes | 0 | Yes | 1 | 0.00025 | 4014 |
| phchp006v2 | SZA | M | 52 | African American |  |  | Yes | 0 | Yes | 1 | 0.00025 | 3929 |
| phchp008v1 | SZ | M | 47 | African American |  |  | Yes | 0 | Yes | 3 | 0.00158 | 1902 |
| phchp009v1 | SZ | M | 55 | African American |  |  | Yes | 0 | Yes | 0 | 0 | 2375 |
| phchp009v3 | SZ | M | 56 | African American |  |  | Yes | 0 | Yes | 0 | 0 | 2195 |
| phchp010v1 | SZA | M | 45 | Caucasian |  |  | Yes | 0 | Yes | 4 | 0.001 | 3993 |
| phchp010v2 | SZA | M | 45 | Caucasian |  |  | Yes | 0 | Yes | 4 | 0.00102 | 3909 |
| phchp010v3 | SZA | M | 45 | Caucasian |  |  | Yes | 0 | Yes | 4 | 0.00105 | 3818 |
| phchp012v1 | SZA | M | 55 | Caucasian |  |  | Yes | 0 | Yes | 0 | 0 | 3972 |
| phchp012v2 | SZA | M | 55 | Caucasian |  |  | Yes | 0 | Yes | 0 | 0 | 3897 |
| phchp012v3 | SZA | M | 55 | Caucasian |  |  | Yes | 0 | Yes | 0 | 0 | 3806 |
| phchp013v1 | SZA | M | 53 | African American |  |  | Yes | 0 | Yes | 2 | 0.0005 | 3962 |
| phchp013v3 | SZA | M | 54 | African American |  |  | Yes | 0 | Yes | 2 | 0.00053 | 3780 |
| phchp016v2 | SZ | M | 54 | African American |  |  | Yes | 0 | Yes | 1 | 0.00052 | 1924 |
| phchp016v3 | SZ | M | 54 | African American |  |  | Yes | 0 | Yes | 1 | 0.00055 | 1831 |
| phchp017v2 | SZA | M | 53 | African American |  |  | Yes | 0 | Yes | 0 | 0 | 556 |
| phchp017v3 | SZA | M | 54 | African American |  |  | Yes | 0 | Yes | 0 | 0 | 385 |
| phchp019v1 | SZ | M | 50 | African American |  |  | Yes | 0 | Yes | 15 | 0.00384 | 3904 |
| phchp019v2 | SZ | M | 51 | African American |  |  | Yes | 0 | Yes | 15 | 0.00396 | 3790 |
| phchp019v3 | SZ | M | 51 | African American |  |  | Yes | 0 | Yes | 15 | 0.00412 | 3643 |
| phchp020v1 | BP | M | 62 | Caucasian |  |  | Yes | 0 | Yes | 0 | 0 | 3886 |
| phchp020v2 | BP | M | 62 | Caucasian |  |  | Yes | 0 | Yes | 0 | 0 | 3805 |
| phchp020v3 | BP | M | 63 | Caucasian |  |  | Yes | 0 | Yes | 0 | 0 | 3668 |
| phchp022v1 | SZ | M | 48 | Caucasian |  |  | Yes | 0 | Yes | 0 | 0 | 3864 |
| phchp022v2 | SZ | M | 48 | Caucasian |  |  | Yes | 0 | Yes | 0 | 0 | 3761 |
| phchp025v1 | SZ | M | 42 | Caucasian |  |  | Yes | 0 | Yes | 3 | 0.00078 | 3858 |
| phchp026v1 | SZA | M | 49 | African American |  |  | Yes | 0 | Yes | 3 | 0.00077 | 3892 |
| phchp026v2 | SZA | M | 49 | African American |  |  | Yes | 0 | Yes | 3 | 0.00079 | 3798 |
| phchp026v3 | SZA | M | 49 | African American |  |  | Yes | 0 | Yes | 3 | 0.00081 | 3693 |
| phchp031v1 | BP | M | 51 | Caucasian |  |  | Yes | 0 | Yes | 1 | 0.00026 | 3866 |
| phchp031v2 | BP | M | 51 | Caucasian |  |  | Yes | 0 | Yes | 1 | 0.00027 | 3768 |
| phchp031v3 | BP | M | 52 | Caucasian |  |  | Yes | 0 | Yes | 1 | 0.00027 | 3657 |
| phchp033v1 | SZA | M | 48 | Caucasian |  |  | Yes | 0 | Yes | 0 | 0 | 3634 |
| phchp038v1 | SZA | M | 58 | African American |  |  | Yes | 0 | Yes | 0 | 0 | 2664 |
| phchp038v2 | SZA | M | 58 | African American |  |  | Yes | 0 | Yes | 0 | 0 | 2557 |
| phchp038v3 | SZA | M | 59 | African American |  |  | Yes | 0 | Yes | 0 | 0 | 2473 |
| phchp041v1 | SZ | M | 62 | African American |  |  | Yes | 0 | Yes | 1 | 0.00027 | 3729 |
| phchp042v1 | SZA | M | 43 | Caucasian |  |  | Yes | 0 | Yes | 0 | 0 | 3713 |
| phchp042v2 | SZA | M | 43 | Caucasian |  |  | Yes | 0 | Yes | 0 | 0 | 3615 |
| phchp042v3 | SZA | M | 44 | Caucasian |  |  | Yes | 0 | Yes | 0 | 0 | 3530 |
| phchp047v1 | SZA | M | 57 | African American |  |  | Yes | 0 | Yes | 0 | 0 | 2817 |
| phchp047v2 | SZA | M | 57 | African American |  |  | Yes | 0 | Yes | 0 | 0 | 2725 |
| phchp047v3 | SZA | M | 58 | African American |  |  | Yes | 0 | Yes | 0 | 0 | 2633 |
| phchp048v1 | SZA | M | 56 | African American |  |  | Yes | 0 | Yes | 3 | 0.00156 | 1925 |
| phchp048v2 | SZA | M | 57 | African American |  |  | Yes | 0 | Yes | 3 | 0.00163 | 1837 |
| phchp048v3 | SZA | M | 57 | African American |  |  | Yes | 0 | Yes | 3 | 0.00173 | 1732 |
| phchp049v1 | SZA | M | 46 | Caucasian |  |  | Yes | 0 | Yes | 0 | 0 | 3494 |
| phchp049v2 | SZA | M | 47 | Caucasian |  |  | Yes | 0 | Yes | 0 | 0 | 3413 |
| phchp053v1 | BP | M | 58 | Caucasian |  |  | Yes | 0 | Yes | 2 | 0.00056 | 3601 |
| phchp053v2 | BP | M | 58 | Caucasian |  |  | Yes | 0 | Yes | 2 | 0.00057 | 3500 |
| phchp053v3 | BP | M | 58 | Caucasian |  |  | Yes | 0 | Yes | 2 | 0.00059 | 3398 |
| phchp055v1 | BP | F | 46 | Caucasian |  |  | Yes | 0 | Yes | 0 | 0 | 3545 |
| phchp055v2 | BP | F | 46 | Caucasian |  |  | Yes | 0 | Yes | 0 | 0 | 3433 |
| phchp055v3 | BP | F | 46 | Caucasian |  |  | Yes | 0 | Yes | 0 | 0 | 3330 |
| phchp057v1 | SZA | M | 47 | Caucasian |  |  | Yes | 0 | Yes | 0 | 0 | 3576 |
| phchp058v1 | SZ | M | 56 | African American |  |  | Yes | 0 | Yes | 2 | 0.00077 | 2593 |
| phchp058v2 | SZ | M | 56 | African American |  |  | Yes | 0 | Yes | 2 | 0.0008 | 2501 |
| phchp058v3 | SZ | M | 56 | African American |  |  | Yes | 0 | Yes | 2 | 0.00084 | 2395 |
| phchp060v1 | SZ | M | 62 | Caucasian |  |  | Yes | 0 | Yes | 0 | 0 | 2068 |
| phchp061v1 | SZ | M | 49 | Caucasian |  |  | Yes | 0 | Yes | 0 | 0 | 3501 |
| phchp061v2 | SZ | M | 49 | Caucasian |  |  | Yes | 0 | Yes | 0 | 0 | 3415 |
| phchp061v3 | SZ | M | 50 | Caucasian |  |  | Yes | 0 | Yes | 0 | 0 | 3193 |
| phchp062v1 | SZ | M | 56 | Caucasian |  |  | Yes | 0 | Yes | 0 | 0 | 3554 |
| phchp062v2 | SZ | M | 56 | Caucasian |  |  | Yes | 0 | Yes | 0 | 0 | 3467 |
| phchp062v3 | SZ | M | 57 | Caucasian |  |  | Yes | 0 | Yes | 0 | 0 | 3372 |
| phchp065v1 | SZA | M | 62 | Caucasian |  |  | Yes | 0 | Yes | 0 | 0 | 3483 |
| phchp065v2 | SZA | M | 62 | Caucasian |  |  | Yes | 0 | Yes | 0 | 0 | 3397 |
| phchp065v3 | SZA | M | 62 | Caucasian |  |  | Yes | 0 | Yes | 0 | 0 | 3299 |
| phchp069v3 | SZ | M | 48 | Caucasian |  |  | Yes | 0 | Yes | 3 | 0.0009 | 3323 |
| phchp070v1 | SZ | M | 52 | African American |  |  | Yes | 0 | Yes | 0 | 0 | 3452 |
| phchp070v2 | SZ | M | 52 | African American |  |  | Yes | 0 | Yes | 0 | 0 | 3329 |
| phchp070v3 | SZ | M | 52 | African American |  |  | Yes | 0 | Yes | 0 | 0 | 3238 |
| phchp072v1 | SZA | M | 60 | Caucasian |  |  | Yes | 0 | Yes | 0 | 0 | 3429 |
| phchp072v2 | SZA | M | 60 | Caucasian |  |  | Yes | 0 | Yes | 0 | 0 | 3317 |
| phchp072v3 | SZA | M | 60 | Caucasian |  |  | Yes | 0 | Yes | 0 | 0 | 3209 |
| phchp074v1 | SZA | F | 46 | African American |  |  | Yes | 0 | Yes | 0 | 0 | 3390 |
| phchp074v2 | SZA | F | 46 | African American |  |  | Yes | 0 | Yes | 0 | 0 | 3281 |
| phchp074v3 | SZA | F | 46 | African American |  |  | Yes | 0 | Yes | 0 | 0 | 3187 |
| phchp076v1 | SZA | F | 41 | African American |  |  | Yes | 0 | Yes | 20 | 0.00597 | 3349 |
| phchp076v2 | SZA | F | 41 | African American |  |  | Yes | 0 | Yes | 20 | 0.00616 | 3247 |
| phchp076v3 | SZA | F | 41 | African American |  |  | Yes | 0 | Yes | 20 | 0.00632 | 3167 |
| phchp079v1 | BP | M | 44 | Caucasian |  |  | Yes | 0 | Yes | 2 | 0.00074 | 2691 |
| phchp079v2 | BP | M | 44 | Caucasian |  |  | Yes | 0 | Yes | 2 | 0.00077 | 2596 |
| phchp079v3 | BP | M | 45 | Caucasian |  |  | Yes | 0 | Yes | 2 | 0.0008 | 2502 |
| phchp081v1 | SZA | M | 53 | African American |  |  | Yes | 0 | Yes | 0 | 0 | 3344 |
| phchp081v2 | SZA | M | 53 | African American |  |  | Yes | 0 | Yes | 0 | 0 | 3235 |
| phchp083v1 | SZ | M | 50 | African American |  |  | Yes | 0 | Yes | 0 | 0 | 3242 |
| phchp083v2 | SZ | M | 50 | African American |  |  | Yes | 0 | Yes | 0 | 0 | 3142 |
| phchp083v3 | SZ | M | 51 | African American |  |  | Yes | 0 | Yes | 0 | 0 | 3049 |
| phchp084v1 | BP | F | 49 | Caucasian |  |  | Yes | 0 | Yes | 0 | 0 | 3205 |
| phchp084v2 | BP | F | 49 | Caucasian |  |  | Yes | 0 | Yes | 0 | 0 | 3133 |
| phchp084v3 | BP | F | 50 | Caucasian |  |  | Yes | 0 | Yes | 0 | 0 | 3035 |
| phchp085v1 | SZA | M | 57 | Caucasian |  |  | Yes | 0 | Yes | 7 | 0.00223 | 3139 |
| phchp085v2 | SZA | M | 57 | Caucasian |  |  | Yes | 0 | Yes | 7 | 0.00229 | 3051 |
| phchp085v3 | SZA | M | 57 | Caucasian |  |  | Yes | 0 | Yes | 7 | 0.00235 | 2974 |
| phchp086v1 | SZ | M | 49 | Caucasian |  |  | Yes | 0 | Yes | 1 | 0.00032 | 3122 |
| phchp086v2 | SZ | M | 49 | Caucasian |  |  | Yes | 0 | Yes | 1 | 0.00033 | 3013 |
| phchp086v3 | SZ | M | 49 | Caucasian |  |  | Yes | 0 | Yes | 1 | 0.00034 | 2905 |
| phchp087v1 | SZA | M | 65 | Caucasian |  |  | Yes | 0 | Yes | 1 | 0.00032 | 3141 |
| phchp087v2 | SZA | M | 66 | Caucasian |  |  | Yes | 0 | Yes | 1 | 0.00033 | 3022 |
| phchp087v3 | SZA | M | 66 | Caucasian |  |  | Yes | 0 | Yes | 1 | 0.00034 | 2931 |
| phchp091v1 | SZA | M | 55 | Caucasian |  |  | Yes | 0 | Yes | 0 | 0 | 2542 |
| phchp091v2 | SZA | M | 55 | Caucasian |  |  | Yes | 0 | Yes | 0 | 0 | 2462 |
| phchp091v3 | SZA | M | 55 | Caucasian |  |  | Yes | 0 | Yes | 0 | 0 | 2364 |
| phchp092v1 | BP | M | 45 | African American |  |  | Yes | 0 | Yes | 0 | 0 | 3021 |
| phchp092v2 | BP | M | 46 | African American |  |  | Yes | 0 | Yes | 0 | 0 | 2888 |
| phchp094v1 | BP | M | 41 | African American |  |  | Yes | 0 | Yes | 0 | 0 | 1696 |
| phchp103v1 | SZA | M | 61 | Caucasian |  |  | Yes | 0 | Yes | 0 | 0 | 938 |
| phchp067v1 | BP | M | 39 | Caucasian |  |  |  | 0 | Yes | 0 | 0 | 3459 |
| phchp067v3 | BP | M | 40 | Caucasian |  |  |  | 0 | Yes | 0 | 0 | 3249 |
| phchp139v1 | SZ | M | 24 | Caucasian | 0 |  |  | 0 | Yes | 2 | 0.00086 | 2332 |
| phchp193v1 | BP | M | 39 | Hispanic |  |  |  | 0 | Yes | 0 | 0 | 2318 |
| phchp226v1 | MDD | M | 29 | Caucasian | 1 |  |  | 0 | Yes | 0 | 0 | 1904 |
| phchp226v2 | MDD | M | 29 | Caucasian | 1 |  |  | 0 | Yes | 0 | 0 | 1822 |
| phchp270v1 | BP | M | 36 | Caucasian |  |  |  | 0 | Yes | 0 | 0 | 3497 |
| phchp270v2 | BP | M | 36 | Caucasian |  |  |  | 0 | Yes | 0 | 0 | 3280 |
