# Supplementary material for: Towards precision medicine for pain: diagnostic biomarkers and repurposed drugs
Source: Mol Psychiatry. 2019 Feb 12;24(4):501–22. doi: 10.1038/s41380-018-0345-5 (PMC6477790; doi:10.1038/s41380-018-0345-5)
Supplement: Supplementary file 2 — Figure S1 and Tables S2-S5 [file 41380_2018_345_MOESM2_ESM.docx]

**Supplementary Information**

**Figure S1:** Pain Scale (1-10)

**
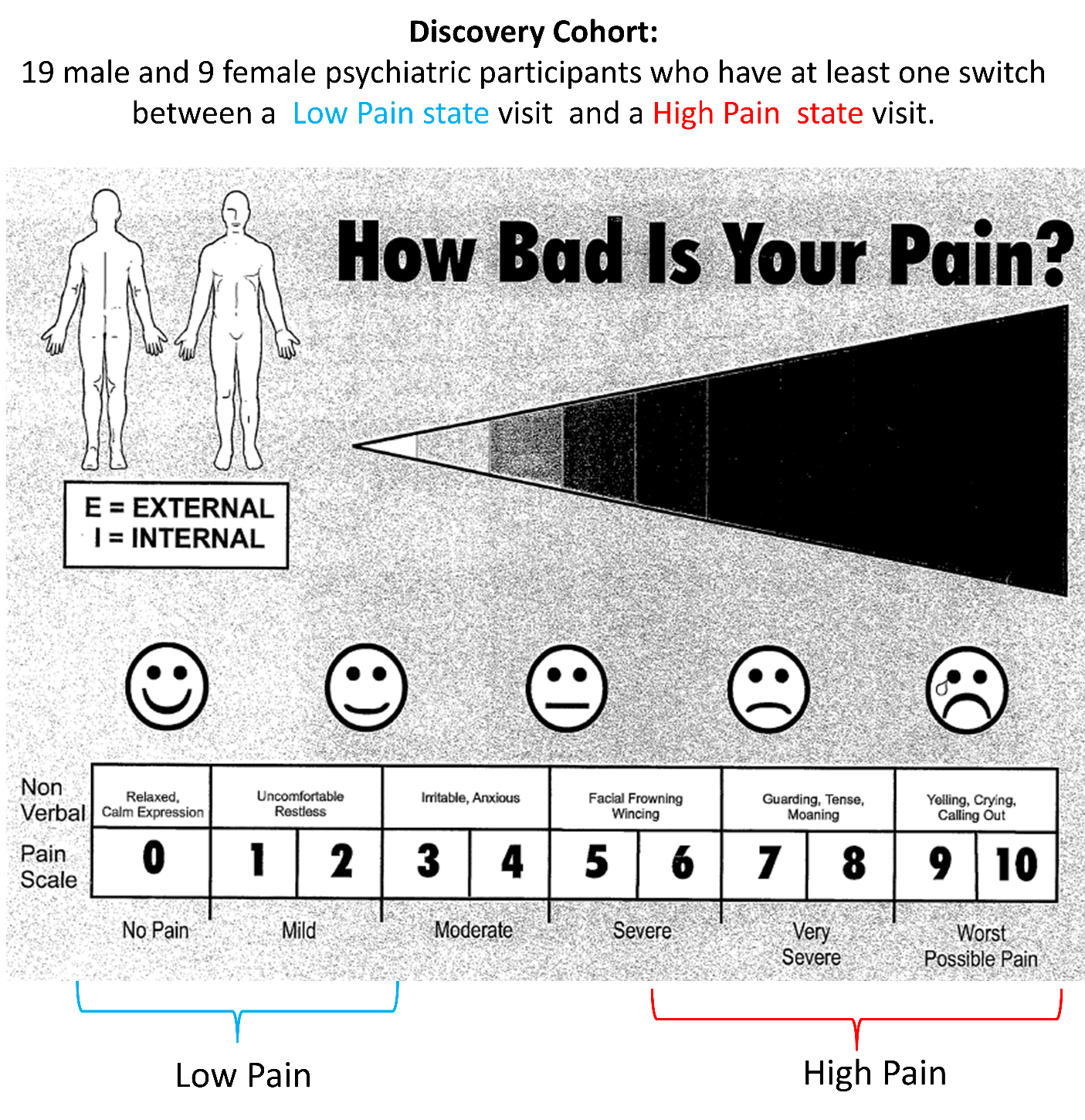
**

**SF-36**

**Item 21 How much bodily pain have you had during the past 4 weeks?**

**1- None**

**2- Very mild**

**3- Mild**

**4- Moderate**

**5- Severe**

**6- Very severe**

**Item 22 During the past 4 weeks, how much did pain interfere with your normal work (including both work outside the home and housework)?**

**1- Not at all**

**2- Slightly**

**3- Moderately**

**4- Quite a bit**

**5- Extremely**

**Table S2. Top Biomarkers for Pain (n=60 genes, 65 probesets) - evidence for involvement in pain.** Red- increased in expression (I) in Pain, Blue- decreased in expression (D). DE- differential expression, AP-Absent/Present. DRG- dorsal root ganglia.

| **Gene Symbol/Gene Name Name** | **Probeset** | **Discovery** (Change) Method/Score | Prior Human Genetic Evidence | Prior Human Nervous Tissue Evidence | Prior Human Peripheral Evidence | Prior Non-human Genetic Evidence | Prior Non-human Nervous Tissue Evidence | Prior Non-human Peripheral Evidence | **Prioritization** Total CFG Score For Pain | **Validation** Anova p-value |
| --- | --- | --- | --- | --- | --- | --- | --- | --- | --- | --- |
| **HLA-DQB1** Major Histocompatibility Complex, Class II, DQ Beta 1 | 212998_x_at | (I) DE/4  51% |  | (D) DRG Neurological Pain [^1^](#_ENREF_1) | (D)Blood Neurological Pain [^2^](#_ENREF_2) |  | (I) Spinal Cord  Neuropathic Pain [^3^](#_ENREF_3) |  | 12 | NS |
| **HLA-DQB1** Major Histocompatibility Complex, Class II, DQ Beta 1 | 211656_x_at | (I) DE/4  59% |  | (D) DRG Neurological Pain [^1^](#_ENREF_1) | (D) Blood Neurological Pain [^2^](#_ENREF_2) |  | (I) Spinal Cord  Neuropathic Pain [^3^](#_ENREF_3) |  | 12 | NS |
| **CALCA** Calcitonin Related Polypeptide Alpha | 210727_at | (D) DE/4  54% | Analgesia[^4^](#_ENREF_4)  Migraine [^5^](#_ENREF_5) |  | (D) Vertebral disc, Neurological Pain [^6^](#_ENREF_6)  (D)  Blood Neuropathic Pain[^7^](#_ENREF_7)  (I) Migraine/Headache [^8^](#_ENREF_8) |  | (I) DRG Pain [^9^](#_ENREF_9)  (I) Neurological Pain [^10^](#_ENREF_10)  (I) Dorsal Horn Neurological Pain [^11^](#_ENREF_11) | (I) blood Acute Pain  [^12^](#_ENREF_12) | 11 | NS |
| **CCDC144B**  Coiled-Coil Domain Containing 144B (Pseudogene) | 1557366_at | (D)  DE/4  56% |  | (I)  Neurological Pain [^1^](#_ENREF_1) |  |  | (D) NAC Neuropathic Pain  [^13^](#_ENREF_13) |  | 10 | NS |
| **CNTN1**  Contactin 1 | 1554784_at | (D) DE/4  52% |  | (D) DRG Neuropathy[^14^](#_ENREF_14) | (D)  CSF[^15^](#_ENREF_15) |  |  |  | 10 | NS |
| **GNG7**  G Protein Subunit Gamma 7 | 1566643_a_at | (D)  DE/4  59% |  | (I) sural nerve Diabetic Neuropathy [^16^](#_ENREF_16) | (I) vertebral disc Neurological Pain [^6^](#_ENREF_6) |  |  |  | 10 | 6.81E-02  Stepwise |
| **HLA-DQB1**  Major Histocompatibility Complex, Class II, DQ Beta 1 | 210747_at | (D)  DE/2  44% |  | (D) DRG Neurological Pain [^1^](#_ENREF_1) | (D) Whole blood Neurological Pain [^2^](#_ENREF_2) |  | (I) Spinal Cord  Neuropathic Pain [^3^](#_ENREF_3) |  | 10 | NS |
| **HLA-DQB1** Major Histocompatibility Complex, Class II, DQ Beta 1 | 211654_x_at | (I)  DE/2  40% |  | (D) DRG Neurological Pain [^1^](#_ENREF_1) | (D) Whole blood Neurological, Pain [^2^](#_ENREF_2) |  | (I) Spinal Cord  Neuropathic Pain [^3^](#_ENREF_3) |  | 10 | NS |
| **ASTN2** Astrotactin 2 | 1554816_at | (I) DE/6  83% | Chronic Migraine [^17^](#_ENREF_17),[^18^](#_ENREF_18),[^19^](#_ENREF_19),[^20^](#_ENREF_20) |  |  |  |  |  | 8 | 1.71E-01  Stepwise |
| **CASP6**  Caspase 6 | 209790_s_at | (I)  DE/4  51% |  |  | (I) vertebral disc Neurological [^6^](#_ENREF_6) |  | DRG Neuropathic pain [^21^](#_ENREF_21) |  | 8 | NS |
| **CCDC85C** Coiled-Coil Domain Containing 85C | 219018_s_at | (D) DE/6  94% |  |  |  |  | (I)  PAG Neuropathic Pain [^13^](#_ENREF_13) |  | 8 | NS |
| **CCND1**  Cyclin D1 | 208712_at | (D)  DE/4  57% |  |  | (D) Serum Chronic Pain  [^22^](#_ENREF_22) |  | (I) (DRG) Neurological Pain [^10^](#_ENREF_10) |  | 8 | NS |
| **CDK6**  Cyclin Dependent Kinase 6 | 224851_at | (I)  DE/4  56%  (I)  AP/2  42% |  |  | (D) Serum Chronic Pain [^22^](#_ENREF_22) |  | (I) Neuropathic Pain [^23^](#_ENREF_23) |  | 8 | NS |
| **CDK6**  Cyclin Dependent Kinase 6 | 224847_at | (I)  DE/4  63% |  |  | (D) Serum Chronic Pain [^22^](#_ENREF_22) |  | (I) Neuropathic Pain [^23^](#_ENREF_23) |  | 8 | NS |
| **COL27A1**  Collagen Type XXVII Alpha 1 Chain | 225293_at | (D)  DE/4  79% |  |  | (D) Lymphoblast Migraine [^24^](#_ENREF_24) |  | (I) PAG Neuropathic Pain [^13^](#_ENREF_13) |  | 8 | 7.47E-01  Stepwise |
| **COL2A1**  Collagen Type II Alpha 1 Chain | 217404_s_at | (D) DE/4  54% |  |  | (I) vertebral disc Neurological Pain [^6^](#_ENREF_6) |  | (I)  PAG  Chronic Neuropathic Pain [^13^](#_ENREF_13) |  | 8 | NS |
| **COMT** Catechol-O-Methyltransferase | 216204_at | (D) DE/4  54% | Neurological Pain [^25^](#_ENREF_25), [^26^](#_ENREF_26)  Chronic Pain MSK [^27^](#_ENREF_27)  [^28^](#_ENREF_28)^,^ [^29^](#_ENREF_29)  [^30^](#_ENREF_30)^,^ [^31^](#_ENREF_31)^,^[^32^](#_ENREF_32)^,^[^33^](#_ENREF_33)^,^[^34^](#_ENREF_34)^,^[^35^](#_ENREF_35)^,^[^36^](#_ENREF_36)^,^[^37^](#_ENREF_37)  Pain, Acute, Thermal [^38^](#_ENREF_38)  Treatments  [^39^](#_ENREF_39)  Pain MSK  [^29^](#_ENREF_29),[^28^](#_ENREF_28),[^27^](#_ENREF_27)  Pain  [^40^](#_ENREF_40)  Morphine  [^41^](#_ENREF_41) |  | (D) Blood Chronic Pain, MSK [^42^](#_ENREF_42) |  |  |  | 8 | NS |
| **COMT** Catechol-O-Methyltransferase | 213981_at | (D) DE/4  54% | Neurological Pain [^25^](#_ENREF_25), [^26^](#_ENREF_26)  Chronic Pain MSK [^27^](#_ENREF_27)  [^28^](#_ENREF_28)^,^ [^29^](#_ENREF_29)  [^30^](#_ENREF_30)^,^ [^31^](#_ENREF_31)^,^[^32^](#_ENREF_32)^,^[^33^](#_ENREF_33)^,^[^34^](#_ENREF_34)^,^[^35^](#_ENREF_35)^,^[^36^](#_ENREF_36)^,^[^37^](#_ENREF_37)  Pain, Acute, Thermal [^38^](#_ENREF_38)  Treatments  [^39^](#_ENREF_39)  Pain MSK  [^29^](#_ENREF_29),[^28^](#_ENREF_28),[^27^](#_ENREF_27)  Pain  [^40^](#_ENREF_40)  Morphine  [^41^](#_ENREF_41) |  | (D) blood Chronic Pain, MSK [^42^](#_ENREF_42) |  |  |  | 8 | NS |
| **DCAF12** DDB1 And CUL4 Associated Factor 12 | 224789_at | (D) DE/6  86% |  |  | (I) Whole blood Neurological, Pain [^2^](#_ENREF_2) |  |  |  | 8 | NS |
| **EDN1**  Endothelin 1 | 1564630_at | (I)  AP/4  56% | Fibromyalgia [^43^](#_ENREF_43) |  | (I)  Blister fluid Chronic Pain  [^44^](#_ENREF_44) |  |  |  | 8 | 8.69E-02  Stepwise |
| **FAM134B** Family With Sequence Similarity 134 Member B | 218510_x_at | (I) DE/4  51%; (I) AP/2  34% | Chronic, Neuropathic Pain [^45^](#_ENREF_45) |  | (I) vertebral disc Neurological Pain [^6^](#_ENREF_6) |  |  |  | 8 | NS |
| **GBP1**  Guanylate Binding Protein 1 | 231578_at | (I)  DE/2  37% | Fibromyalgia [^46^](#_ENREF_46) | (D) Neurological Pain [^1^](#_ENREF_1) |  |  |  |  | 8 | 3.26E-01  Stepwise |
| **HLA-DRB1**  Major Histocompatibility Complex, Class II, DR Beta 1 | 208306_x_at | (I)  AP/4  52% | Migraine[^47^](#_ENREF_47) |  | (I) Whole blood Neurological Pain [^2^](#_ENREF_2) |  |  |  | 8 | NS |
| **HTR2A** 5-Hydroxytryptamine Receptor 2A | 211616_s_at | (D) DE/4  52% | Neurological, Pain [^48^](#_ENREF_48)  Chronic, MSK [^31^](#_ENREF_31),[^49^](#_ENREF_49),[^50^](#_ENREF_50)  Fibromyalgia [^51^](#_ENREF_51),[^52^](#_ENREF_52),  [^53^](#_ENREF_53)  Pain, Acute, disease/lesion [^54^](#_ENREF_54)  Pain  [^40^](#_ENREF_40),[^55^](#_ENREF_55) |  | (D) whole blood, Neuropathic [^7^](#_ENREF_7) |  |  |  | 8 | NS |
| **LOXL2**  Lysyl Oxidase Like 2 | 228808_s_at | (D) DE/4  59% |  |  | (I) vertebral disc Neurological Pain [^6^](#_ENREF_6) |  | (I)  PFC  Chronic Neuropathic Pain [^13^](#_ENREF_13) |  | 8 | NS |
| **LY9** Lymphocyte Antigen 9 | 231124_x_at | (I) DE/6  90% |  |  |  |  | (D)  NAC  Chronic Neuropathic Pain [^13^](#_ENREF_13) |  | 8 | NS |
| **NF1**  Neurofibromin 1 | 212676_at | (I)  DE/4  59% | Migraine [^56^](#_ENREF_56) |  | (I) vertebral disc Neurological Pain [^6^](#_ENREF_6) |  |  |  | 8 | NS |
| **PNOC**  Prepronociceptin | 205901_at | (I) DE/4  62% |  |  | (D) vertebral disc Neurological Pain  [^6^](#_ENREF_6)  (I) whole blood Neuropathic Pain [^7^](#_ENREF_7) |  | (I)  PAG  Chronic Neuropathic Pain [^13^](#_ENREF_13) |  | 8 | NS |
| **SHMT1**  Serine Hydroxymethyltransferase 1 | 217304_at | (D)  DE/2  43% | Musculoskeletal Pain [^57^](#_ENREF_57) | (D)  Neurological Pain [^1^](#_ENREF_1) |  |  |  |  | 8 | NS |
| **TCF15** Transcription Factor 15 (Basic Helix-Loop-Helix) | 207306_at | (D) DE/6  94% |  |  |  |  | (I)  PFC  Chronic Neuropathic Pain [^13^](#_ENREF_13) |  | 8 | NS |
| **TOP3A**  Topoisomerase (DNA) III Alpha | 214300_s_at | (D)  DE/4  51% |  | (D) Neurological Pain [^1^](#_ENREF_1) |  |  |  |  | 8 | NS |
| **TSPO**  Translocator Protein | 202096_s_at | (I)  DE/2  38% | Neuraxial Pain[^58^](#_ENREF_58) |  | (I) vertebral disc Neurological Pain [^6^](#_ENREF_6) |  | (I)  PAG Neuropathic Pain [^13^](#_ENREF_13)  (I)  DRG) Neurological Pain [^10^](#_ENREF_10) |  | 8 | NS |
| **VEGFA**  Vascular Endothelial Growth Factor A | 212171_x_at | (I)  AP/4  65% | Neuraxial Pain[^59^](#_ENREF_59) |  | (I)  Blood Steroid [^60^](#_ENREF_60)  (I)  Chronic Pain  [^61^](#_ENREF_61)  (I)  serum Acute Pain MSK [^62^](#_ENREF_62) |  |  |  | 8 | NS |
| **WNK1** WNK Lysine Deficient Protein Kinase 1 | 1555068_at | (D) DE/6  92% | Chronic Neuropathic Pain [^63^](#_ENREF_63)  Pain  [^40^](#_ENREF_40) |  |  |  |  |  | 8 | NS |
| **ZNF429**  Zinc Finger Protein 429 | 1561270_at | (D)  DE/2  37% | Pain MSK [^64^](#_ENREF_64)  Analgesia [^65^](#_ENREF_65) | (I) Neurological Pain [^1^](#_ENREF_1) |  |  |  |  | 8 | NS |
| **ZYX**  Zyxin | 238016_s_at | (D) DE/4  57% |  |  | (I) Whole blood Neurological Pain [^2^](#_ENREF_2) |  | (I)  PAG  Chronic Neuropathic Pain [^13^](#_ENREF_13) |  | 8 | NS |
| **(AF087971)**  **PBRM1**  Polybromo 1 | 1561067_at | (I) AP/6  90% |  |  |  |  |  |  | 6 | NS |
| **(AF090920)**  **PPFIBP2**  PPFIA Binding Protein 2 | 234739_at | (I) AP/6  94% |  |  |  |  |  |  | 6 | NS |
| **(H05785)**  **LRRC75A**  Leucine Rich Repeat Containing 75A | 236913_at | (D) AP/6  97% |  |  |  |  |  |  | 6 | NS |
| **(Hs.596713)**  **PPP1R14B**  Protein Phosphatase 1 Regulatory Inhibitor Subunit 14B | 226138_s_at | (D) DE/6  90% |  |  |  |  |  |  | 6 | 6.28E-02  Stepwise |
| **(Hs.609761)**  **SFPQ**  Splicing Factor Proline And Glutamine Rich | 244331_at | (D) DE/6  98% |  |  |  |  |  |  | 6 | NS |
| **(Hs.659426)**  **PHC3**  Polyhomeotic Homolog 3 | 240599_x_at | (D) DE/6  92% |  |  |  |  |  |  | 6 | NS |
| **(Hs.666804)**  **MFAP3 Microfibril Associated Protein 3** | 240949_x_at | (D) DE/6  81% |  |  |  |  |  |  | 6 | ***6.03E-04***  ***Nominal*** |
| **(Hs.677263)**  **SMURF2** (SMAD Specific E3 Ubiquitin Protein Ligase 2) | 216444_at | (D) AP/6  100%  (D)  DE/4  71% |  |  |  |  |  |  | 6 | NS |
| **(Hs.696420)**  **MTERF1**  Mitochondrial Transcription Termination Factor 1 | 243125_x_at | (D) DE/6  100% |  |  |  |  |  |  | 6 | NS |
| **CLSPN** Claspin | 242150_at | (I) AP/6  95% |  |  |  |  |  |  | 6 | NS |
| **DENND1B** DENN Domain Containing 1B | 1557309_at | (I) DE/6  90%; (I)  AP/2  40% |  |  |  |  |  |  | 6 | NS |
| **DNAJC18**  DnaJ Heat Shock Protein Family (Hsp40) Member C18 | 227166_at | (I) DE/6  94% |  |  |  |  |  |  | 6 | NS |
| **ELAC2**  **ElaC Ribonuclease Z 2** | 201766_at | (D)  DE/4  52% | Fibromyalgia[^66^](#_ENREF_66) |  |  |  |  |  | 6 | **4.11E-02**  ***Nominal*** |
| **GSPT1** G1 To S Phase Transition 1 | 215438_x_at | (D) DE/6  94% |  |  |  |  |  |  | 6 | NS |
| **HRAS** HRas Proto-Oncogene, GTPase | 212983_at | (I) DE/6  97% |  |  |  |  |  |  | 6 | NS |
| **Hs.554262** | 210703_at | (I) AP/6  100% |  |  |  |  |  |  | 6 | NS |
| **MBNL3** Muscleblind Like Splicing Regulator 3 | 219814_at | (D) DE/6  92% |  |  |  |  |  |  | 6 | NS |
| **MCRS1** Microspherule Protein 1 | 202556_s_at | (I) DE/6  90% |  |  |  |  |  |  | 6 | NS |
| **OSBP2** Oxysterol Binding Protein 2 | 1569617_at | (D) DE/6  94% |  |  |  |  |  |  | 6 | NS |
| **PIK3CD** **Phosphatidylinositol-4,5-Bisphosphate 3-Kinase Catalytic Subunit Delta** | 211230_s_at | (D) DE/6  83% |  |  |  |  |  |  | 6 | ***1.59E-02***  ***Nominal*** |
| **PTN** Pleiotrophin | 211737_x_at | (D) DE/6  92% |  |  |  |  |  |  | 6 | NS |
| **RAB33A** RAB33A, Member RAS Oncogene Family | 206039_at | (I) DE/6  90% |  |  |  |  |  |  | 6 | NS |
| **RALGAPA2** Ral GTPase Activating Protein Catalytic Alpha Subunit 2 | 231826_at | (D) DE/6  97% |  |  |  |  |  |  | 6 | NS |
| **SEPT7P2** Septin 7 Pseudogene 2 | 1569973_at | **(I) DE/6**  **100%**  (I)  AP/2  39% |  |  |  |  |  |  | 6 | NS |
| **SVEP1**  **Sushi, Von Willebrand Factor Type A, EGF And Pentraxin Domain Containing 1** | 236927_at | (I)  DE/2  49% | Migraine [^56^](#_ENREF_56) |  |  |  | (D)  NAC Neuropathic Pain [^13^](#_ENREF_13) |  | 6 | ***2.17E-02***  ***Nominal*** |
| **TNFRSF11B**  **TNF Receptor Superfamily Member 11b** | 204932_at | (D)  DE/2  37% | Cancer Pain [^67^](#_ENREF_67) |  | (I) vertebral disc Neurological Pain [^6^](#_ENREF_6)  (I) Serum Chronic Pain  [^68^](#_ENREF_68) |  |  |  | 6 | ***2.67E-02***  ***Nominal*** |
| **YBX3** Y-Box Binding Protein 3 | 201160_s_at | (D) DE/6  94% |  |  |  |  |  |  | 6 | NS |
| **ZNF441** Zinc Finger Protein 441 | 1553193_at | (I) AP/6  95%  (I)  DE/2  35% |  |  |  |  |  |  | 6 | NS |
| **ZNF91**  Zinc Finger Protein 91 | 244259_s_at | (I) AP/6  95% |  |  |  |  |  |  | 6 | 6.37E-01/2  Stepwise |

**Table S3. Top biomarkers for pain - Evidence for involvement in other psychiatric and related disorders.** In the same direction of expression as pain**.** Red- increased in expression (I) in Pain, Blue- decreased in expression (D). DE- differential expression, AP-Absent/Present.

| **Gene Symbol/Gene Name Name** | **Probeset** | **Discovery (Change) Method/Score** | **Prioritization Total CFG Score For Pain** | **Validation Anova p-value** | **Prior human genetic evidence for other disorders**  **2pts.** | **Prior human Brain expression evidence**  **for other disorders**  **4 pts** | **Prior human peripheral evidence for other disorders**  **2pts** | **Prior Non-human genetic evidence for other disorders**  **1pt.** | **Prior Non-human Brain expression evidence**  **for other disorders**  **2pts.** | **Prior Non-human peripheral evidence for other disorders**  **1pt.** | **External CFG for Other Dx** |
| --- | --- | --- | --- | --- | --- | --- | --- | --- | --- | --- | --- |
| **HTR2A** 5-Hydroxytryptamine Receptor 2A | 211616_s_at | (D) DE/4  52% | 8 | NS | Alcoholism [^69^](#_ENREF_69)  BP [^70^](#_ENREF_70) [^71^](#_ENREF_71) [^72^](#_ENREF_72) [^70^](#_ENREF_70)^,^ [^73^](#_ENREF_73)^,^ [^74^](#_ENREF_74)  Depression [^75-77^](#_ENREF_75) [^78^](#_ENREF_78)  Mood [^79^](#_ENREF_79)  OCD [^80^](#_ENREF_80)  Addictions [^81^](#_ENREF_81)^,^ [^82^](#_ENREF_82) [^83^](#_ENREF_83) [^84^](#_ENREF_84) [^85^](#_ENREF_85)    Suicide [^79^](#_ENREF_79)^,^ [^86^](#_ENREF_86) [^87-90^](#_ENREF_87) | (D) HIP BP [^91^](#_ENREF_91)  (D) HIP SZ, Depression[^92^](#_ENREF_92)  (D) DLPFC BP [^92^](#_ENREF_92)  (D) Temporal Cortex SZ [^93^](#_ENREF_93)  (D) HIP BP, SZ[^94^](#_ENREF_94)Suicide [^95^](#_ENREF_95)  (D) PFC Aging [^96^](#_ENREF_96)  (D) frontal cortex Suicide [^97^](#_ENREF_97)  (D) BA46 Suicide [^98^](#_ENREF_98)  (D) Brain BP [^99^](#_ENREF_99)  (I) AMY,Frontopolar cortex Suicide [^100^](#_ENREF_100)  (D) PFC SZ[^101^](#_ENREF_101)  (D) DLFPC Suicide [^102^](#_ENREF_102) | (D) Lymphocyte SZ [^103^](#_ENREF_103)  (D) PBMC SZ [^104^](#_ENREF_104)  (D) Platelets Suicide [^105^](#_ENREF_105) | Anxiety  [^106^](#_ENREF_106) | (D) PFC SZ [^107^](#_ENREF_107)  (D) Frontal cortex Depression, SZ [^108^](#_ENREF_108)  (D) PFC Hallucinogens[^109^](#_ENREF_109)  (D) AMY PTSD [^110^](#_ENREF_110)  (I) AMY Depression[^111^](#_ENREF_111) |  | 13 |
| **CDK6**  Cyclin Dependent Kinase 6 | 224847_at | (I)  DE/4  63% | 8 | NS | Circadian abnormalities [^112^](#_ENREF_112)  Longevity [^113^](#_ENREF_113),[^114^](#_ENREF_114)  Alcohol [^115^](#_ENREF_115) | (I) PFC SZ [^116^](#_ENREF_116)  (I) Brain SZ [^117^](#_ENREF_117) | (I) lymphoblastoid ASD [^118^](#_ENREF_118)  (I)Blood Female Suicide [^119^](#_ENREF_119)  (I) Blood M-BP Suicide [^120^](#_ENREF_120) |  | (I) AMY MDD [^121^](#_ENREF_121) |  | 10 |
| **CDK6**  Cyclin Dependent Kinase 6 | 224851_at | (I)  DE/4  56%  (I)  AP/2  42% | 8 | NS | Circadian abnormalities [^112^](#_ENREF_112)  Longevity [^113^](#_ENREF_113),  [^114^](#_ENREF_114)  Alcohol [^115^](#_ENREF_115) | (I) PFC SZ [^116^](#_ENREF_116)  (I) Brain SZ [^117^](#_ENREF_117) | (I) lymphoblastoid ASD [^118^](#_ENREF_118)  (I)Blood Female Suicide [^119^](#_ENREF_119)  (I) Blood M-BP Suicide [^120^](#_ENREF_120) |  | (I) AMY MDD [^121^](#_ENREF_121) |  | 10 |
| **HLA-DQB1** Major Histocompatibility Complex, Class II, DQ Beta 1 | 212998_x_at  211656_x_at | (I) DE/4  (I) DE/4  59% | 8 | NS  NS | Longevity [^122^](#_ENREF_122) ,[^123^](#_ENREF_123)  SZ [^124^](#_ENREF_124)^,^ [^125^](#_ENREF_125) | (I) Superior temporal cortex (BA 22) SZ [^126^](#_ENREF_126) | (I) Blood SZ [^127^](#_ENREF_127)  (I) Blood Suicide[^128^](#_ENREF_128) [^129^](#_ENREF_129)  (I) PBMC Stress [^130^](#_ENREF_130) PTSD [^131^](#_ENREF_131)  (I) Leukocytes Depression[^132^](#_ENREF_132) |  | (I) CP, NAC (D) AMY Alcoholism [^133^](#_ENREF_133) |  | 10 |
| **WNK1** WNK Lysine Deficient Protein Kinase 1 | 1555068_at | (D) DE/6  92% | 8 | NS | Depression [^134^](#_ENREF_134) | (D) NAC Alcohol [^135^](#_ENREF_135) | (D) Blood Suicide [^129^](#_ENREF_129), [^120^](#_ENREF_120) |  | (D) PFC (male) BP, Stress [^136^](#_ENREF_136) |  | 10 |
| **(AF087971)**  **PBRM1**  Polybromo 1 | 1561067_at | (I) AP/6  90% | 6 | NS | CNV,MDD [^137^](#_ENREF_137)  BP [^138-140^](#_ENREF_138) [^141-143^](#_ENREF_141)  Mood, Psychosis [^144^](#_ENREF_144)  Depression [^139^](#_ENREF_139)  MDD [^139^](#_ENREF_139) [^140^](#_ENREF_140) SZ [^141^](#_ENREF_141)^,^ [^145^](#_ENREF_145)  Longevity[^146^](#_ENREF_146) | (I) DLPFC BP [^139^](#_ENREF_139) | (I) Blood Hallucinations[^147^](#_ENREF_147)  (I) Blood Mood [^148^](#_ENREF_148)  (I) Blood Male Suicide [^129^](#_ENREF_129)  (I) Blood Female Suicide [^119^](#_ENREF_119) |  | (I) AMY MDD [^121^](#_ENREF_121)  (I) AMY(male) BP, Stress [^136^](#_ENREF_136)  (I) Brain Stimulants [^149^](#_ENREF_149) |  | 10 |
| **(Hs.666804) MFAP3 Microfibril Associated Protein 3** | 240949_x_at | (D) DE/6  81% | 6 | ***6.03E-04/4***  ***Nominal*** | SZ  [^124^](#_ENREF_124) | (D)Superior frontal cortex  Alcohol  [^150^](#_ENREF_150) | (D)Blood  Suicide  [^129^](#_ENREF_129),[^120^](#_ENREF_120) |  | (D)  AMY  Stress  [^121^](#_ENREF_121) |  | 10 |
| **CCND1**  Cyclin D1 | 208712_at | (D)  DE/4  57% | 8 | NS |  | (D) Frontal motor cortex Alcohol [^151^](#_ENREF_151)  (D) hippocampus Alcohol [^152^](#_ENREF_152)  (D) ACC MDD [^153^](#_ENREF_153) | (D) Peripheral blood Stress [^154^](#_ENREF_154) | Addiction Alcohol [^155^](#_ENREF_155) | (D) Amygdala) Hallucinogens [^156^](#_ENREF_156)  (D) Amygdala Addiction Alcohol [^133^](#_ENREF_133) |  | 9 |
| **CNTN1**  Contactin 1 | 1554784_at | (D) DE/4  52% | 10 | NS | BP,SZ [^157^](#_ENREF_157); [^158^](#_ENREF_158)  MDD [^134^](#_ENREF_134)  Suicide [^159^](#_ENREF_159) | (D) Brain BP [^99^](#_ENREF_99)  (D) HIP BP [^160^](#_ENREF_160)  (D) Forebrain neural progenitor cells SZ [^161^](#_ENREF_161)  (D) supragenual (BA24) anterior cingulated cortex SZ [^162^](#_ENREF_162)  (D) anterior PFC SZA [^163^](#_ENREF_163) | (D) lymphocyte SZ [^164^](#_ENREF_164)  (D) Blood Female Suicide [^119^](#_ENREF_119) |  |  |  | 8 |
| **GBP 1**  Guanylate Binding Protein 1 | 231578_at | (I)  DE/2  37% | 8 | 3.26E-01/2  Stepwise |  | (I) Hippocampus, amygdala, gyrus cinguli ,pons MDD [^165^](#_ENREF_165)  (I) amygdala SZ [^166^](#_ENREF_166)  (I) left side superior frontal gyrus SZ [^167^](#_ENREF_167)  (I) Brain Suicide [^165^](#_ENREF_165) | (I) leukocytes PTSD [^168^](#_ENREF_168) |  | (I) hippocampal and prefrontal cortex MDD [^165^](#_ENREF_165) |  | 8 |
| **HLA-DQB1** Major Histocompatibility Complex, Class II, DQ Beta 1 | 211654_x_at | (I)  DE/2  40% | 8 | NS |  | (I) superior temporal cortex SZ [^126^](#_ENREF_126) | (I) monocytes Stress [^130^](#_ENREF_130)  (I) PBMC PTSD [^131^](#_ENREF_131) |  | (I) Caudate putamen Addiction Alcohol[^133^](#_ENREF_133) |  | 8 |
| **PNOC**  Prepronociceptin | 205901_at | (I) DE/4  62% | 7 | NS | Addictions  [^169^](#_ENREF_169) | (I) DLPFC BP,SZ  [^170^](#_ENREF_170)  (I) AMY, cingulate cortex MDD [^171^](#_ENREF_171)  (I) Forebrain neural cells  SZ  [^161^](#_ENREF_161) | (I) Fibroblasts  SZ [^172^](#_ENREF_172) |  | (I) NAC Stress [^173^](#_ENREF_173)  (I) Amygdala MDD [^111^](#_ENREF_111) |  | 8 |
| **GSPT1** G1 To S Phase Transition 1 | 215438_x_at | (D) DE/6  94% | 6 | NS |  | (D) Brain BP [^99^](#_ENREF_99) | (D) Blood Suicide [^129^](#_ENREF_129)  (D) Leukocytes Depression[^132^](#_ENREF_132) |  | (D) AMY Depression[^121^](#_ENREF_121) |  | 8 |
| **(Hs.609761)**  **SFPQ**  Splicing Factor Proline And Glutamine Rich | 244331_at | (D) DE/6  98% | 6 | NS |  | NAC altered MDD [^174^](#_ENREF_174)  (D) superior frontal cortex Alcohol [^150^](#_ENREF_150)  (D) PFC MDD [^175^](#_ENREF_175) | (D) Blood Female Suicide [^119^](#_ENREF_119) |  | (D) VT Hallucinogens[^156^](#_ENREF_156)  (D) PFC (male) Stress, BP [^136^](#_ENREF_136)  (D) Brain Alcohol Addiction[^176^](#_ENREF_176) |  | 8 |
| **ZNF91** Zinc Finger Protein 91 | 244259_s_at | (I) AP/6  95% | 6 | 6.37E-01/2  Stepwise | Circadian abnormalities [^112^](#_ENREF_112) | (I) Temporal cortex Alcoholism [^177^](#_ENREF_177) (I) DLPFC PTSD [^178^](#_ENREF_178) | (I) Blood PTSD [^179^](#_ENREF_179) |  |  |  | 8 |
| **COMT** Catechol-O-Methyltransferase | 216204_at  213981_at | (D) DE/4  54%  (D) DE/4 | 8 | NS  NS | OCD [^180^](#_ENREF_180) [^181^](#_ENREF_181) [^182^](#_ENREF_182) [^183-185^](#_ENREF_183) [^186^](#_ENREF_186)    BP [^187^](#_ENREF_187) [^182^](#_ENREF_182) [^188^](#_ENREF_188) [^189^](#_ENREF_189)^,^ [^190^](#_ENREF_190) [^191^](#_ENREF_191)^,^ [^192^](#_ENREF_192)  Anxiety[^193^](#_ENREF_193) [^194^](#_ENREF_194) [^195^](#_ENREF_195) [^196^](#_ENREF_196) [^197^](#_ENREF_197) [^198^](#_ENREF_198) [^199^](#_ENREF_199) [^200^](#_ENREF_200) [^201^](#_ENREF_201)  SZ [^202^](#_ENREF_202) [^203^](#_ENREF_203) [^204^](#_ENREF_204) [^205^](#_ENREF_205) [^206^](#_ENREF_206) [^207^](#_ENREF_207) [^192^](#_ENREF_192)^,^ [^208^](#_ENREF_208) [^209^](#_ENREF_209)  Aggression [^210^](#_ENREF_210) [^211^](#_ENREF_211) [^212^](#_ENREF_212)  Suicide [^213^](#_ENREF_213) [^214^](#_ENREF_214) [^215^](#_ENREF_215) [^216^](#_ENREF_216)  [^217^](#_ENREF_217)  Thermal [^218^](#_ENREF_218) Stimulants[^219^](#_ENREF_219)  Intellect[^220^](#_ENREF_220)  Mood [^209^](#_ENREF_209)  ADHD [^186^](#_ENREF_186)  Depression[^78^](#_ENREF_78) [^221-223^](#_ENREF_221)   PTSD [^224^](#_ENREF_224) [^225^](#_ENREF_225)  Alcohol[^226^](#_ENREF_226) |  | (D) Blood Alcoholism [^227^](#_ENREF_227)  (D) Blood SZ [^228^](#_ENREF_228)  (D) Leukocytes SZ [^229^](#_ENREF_229)  (D) PBMC Stress [^130^](#_ENREF_130)  (D) Blood Suicide[^119^](#_ENREF_119) | SZ[^230^](#_ENREF_230)^,^ [^231^](#_ENREF_231)  Alcoholism [^155^](#_ENREF_155) | (D) PFC Anxiety, OCD, SZ[^232^](#_ENREF_232)  (D) Brain Anxiety [^233^](#_ENREF_233)  (D) Male HIP, AMY Anxiety [^234^](#_ENREF_234) |  | 7 |
| **VEGFA**  Vascular Endothelial Growth Factor A | 212171_x_at | (I)  AP/4  65% | 8 | NS |  | (I) CA3/2 Stratum oriens SZ [^235^](#_ENREF_235)  (I) Prefrontal cortex SZ [^236^](#_ENREF_236)  (I) hippocampus SZ [^237^](#_ENREF_237) | (I) monocytes Stress [^130^](#_ENREF_130)  (I) plasma MDD [^238^](#_ENREF_238)  (I) plasma BP [^239^](#_ENREF_239) | MDD [^240^](#_ENREF_240) |  |  | 7 |
| **(H05785)**  **LRRC75A**  Leucine Rich Repeat Containing 75A | 236913_at | (D) AP/6  97% | 6 | NS |  | (D) Brain BP [^99^](#_ENREF_99)  (D) DLPFC SZ [^241^](#_ENREF_241) | (D) Blood  Male BP Suicide [^120^](#_ENREF_120) | Alcohol Addiction[^155^](#_ENREF_155) |  |  | 7 |
| **CALCA** Calcitonin Related Polypeptide Alpha | 210727_at | (D) DE/4  54% | 7 | NS |  | (D) Frontal, motor cortex Alcohol[^151^](#_ENREF_151) |  |  | (D) Medullae Oblongata Anxiety [^242^](#_ENREF_242) |  | 6 |
| **LOXL2**  Lysyl Oxidase Like 2 | 228808_s_at | (D) DE/4  59% | 7 | NS |  | (D) anterior PFC BP [^163^](#_ENREF_163) | (D) Male-BP Suicide[^120^](#_ENREF_120) |  |  |  | 6 |
| **HRAS** HRas Proto-Oncogene, GTPase | 212983_at | (I) DE/6  97% | 6 | NS | BP, SZ [^157^](#_ENREF_157)  Longevity [^243^](#_ENREF_243) |  | mRNA Suicide [^244^](#_ENREF_244) |  | (I) NAC Alcohol [^245^](#_ENREF_245) |  | 6 |
| **(Hs.696420)**  **MTERF1**  Mitochondrial Transcription Termination Factor 1 | 243125_x_at | (D) DE/6 | 6 | NS |  | (D) DPFC BA 46 PTSD [^246^](#_ENREF_246) | (D) Blood Universal Suicide  [^120^](#_ENREF_120) |  |  |  | 6 |
| **PIK3CD** **Phosphatidylinositol-4,5-Bisphosphate 3-Kinase Catalytic Subunit Delta** | 211230_s_at | (D) DE/6  83% | 6 | ***1.59E-02/4***  ***Nominal*** | Longevity [^247^](#_ENREF_247)  SZ [^248^](#_ENREF_248) |  | (D) PBMC Stress [^130^](#_ENREF_130)  (D) Blood Suicide [^120^](#_ENREF_120)  mRNA Suicide [^244^](#_ENREF_244) |  | (D) NAC Alcohol [^133^](#_ENREF_133) |  | 6 |
| **PTN** Pleiotrophin | 211737_x_at | (D) DE/6  92% | 6 | NS | SZ [^145^](#_ENREF_145) [^249^](#_ENREF_249) |  | mRNA Suicide [^244^](#_ENREF_244) |  | (D) HIP Stress[^250^](#_ENREF_250) |  | 6 |
| **YBX3** Y-Box Binding Protein 3 | 201160_s_at | (D) DE/6  94% | 6 | NS |  | (D) DLPFC BP,SZ  [^170^](#_ENREF_170) | (D) Blood Male Suicide  [^129^](#_ENREF_129) |  |  |  | 6 |
| **NF1**  Neurofibromin 1 | 212676_at | (I)  DE/4  59% | 8 | NS |  | Differentially expressed ACC (BA 24) BP [^251^](#_ENREF_251) |  | Addiction Alcohol [^155^](#_ENREF_155) | (I) VS PTSD [^110^](#_ENREF_110) |  | 5 |
| **SVEP1**  **Sushi, Von Willebrand Factor Type A, EGF And Pentraxin Domain Containing 1** | 236927_at | (I)  DE/2  49% | 6 | ***2.17E-02/4***  ***Nominal*** |  | (I) Hippocampus SZ [^252^](#_ENREF_252) |  | Alcohol [^155^](#_ENREF_155) |  |  | 5 |
| **(Hs.677263)Smurf2**  SMAD Specific E3 Ubiquitin Protein Ligase 2 | 216444_at | (D) AP/6  100%  (D)  DE/4  71% | 6 | NS |  |  | (D) Blood Suicide  [^129^](#_ENREF_129),[^120^](#_ENREF_120) |  | (D) VM PFC Stress [^253^](#_ENREF_253) | Intervertebral disc Aging [^254^](#_ENREF_254) | 5 |
| **ASTN2** Astrotactin 2 | 1554816_at | (I) DE/6  83% | 8 | 1.71E-01  Stepwise | Stimulants[^255^](#_ENREF_255)  SZ[^256^](#_ENREF_256) [^257^](#_ENREF_257) [^206^](#_ENREF_206)  Autism[^258^](#_ENREF_258)  Autism  CNV[^259^](#_ENREF_259)  BP[^257^](#_ENREF_257) |  | (I) Female Blood Suicide[^119^](#_ENREF_119) |  |  |  | 4 |
| **CASP6**  Caspase 6 | 209790_s_at | (I)  DE/4  51% | 8 | NS |  | (I) Dorsolateral prefrontal cortex BP [^260^](#_ENREF_260) |  |  |  |  | 4 |
| **FAM134B** Family With Sequence Similarity 134 Member B | 218510_x_at | (I) DE/4  51%; (I) AP/2  34% | 8 | NS | Antisocial Personality [^261^](#_ENREF_261) |  | (I) Male BP SI, Universal SI[^120^](#_ENREF_120) |  | (I) VT Hallucinogens [^156^](#_ENREF_156) |  | 4 |
| **HLA-DQB1**  Major Histocompatibility Complex, Class II, DQ Beta 1 | 210747_at | (D)  DE/2  44% | 8 | NS |  |  | (D) leukocytes Stress, [^262^](#_ENREF_262) |  | (D) Amygdala Addictions, Alcohol [^133^](#_ENREF_133) |  | 4 |
| **ZYX**  Zyxin | 238016_s_at | (D) DE/4  57% | 7 | NS |  |  | (D) Blood MDD  [^263^](#_ENREF_263) |  | (D)  AMY  MDD  [^264^](#_ENREF_264) |  | 4 |
| **DNAJC18**  DnaJ Heat Shock Protein Family (Hsp40) Member C18 | 227166_at | (I) DE/6 | 6 | NS |  | ACC (BA 24) BP [^265^](#_ENREF_265) |  |  |  |  | 4 |
| **MCRS1** Microspherule Protein 1 | 202556_s_at | (I) DE/6 | 6 | NS |  | (I) Pituitary Depression[^266^](#_ENREF_266) |  |  |  |  | 4 |
| **OSBP2** Oxysterol Binding Protein 2 | 1569617_at | (D) DE/6 | 6 | NS | SZ [^267^](#_ENREF_267) |  | (D) Blood Suicide [^128^](#_ENREF_128)^,^ [^129^](#_ENREF_129)  (D) SH-SY5Y cells Cocaine [^268^](#_ENREF_268) |  |  |  | 4 |
| **RAB33A** RAB33A, Member RAS Oncogene Family | 206039_at | (I) DE/6 | 6 | NS |  | (I) Frontal Cortex Alcohol [^269^](#_ENREF_269)  (I) Stress[^270^](#_ENREF_270)  (I)PFC,ACC,MDD[^153^](#_ENREF_153) |  |  |  |  | 4 |
| **TSPO**  Translocator Protein | 202096_s_at | (I)  DE/2  38% | 6 | NS |  | (I) Forebrain neural progenitor cells SZ [^161^](#_ENREF_161) |  |  |  |  | 4 |
| **GNG7**  G Protein Subunit Gamma 7 | 1566643_a_at | (D)  DE/4  59% | 10 | 6.81E-02/2  Stepwise |  |  |  |  | (D)  NAC  Alcohol [^271^](#_ENREF_271)  (D)  PFC  Hallucinogens [^156^](#_ENREF_156)  (D)  PFC (male)  BP/Stress [^136^](#_ENREF_136)  (D)  AMY  MDD [^111^](#_ENREF_111) |  | 2 |
| **COL27A1**  Collagen Type XXVII Alpha 1 Chain | 225293_at | (D)  DE/4  79% | 8 | 7.47E-01/2  Stepwise | Tourette syndrome [^272^](#_ENREF_272) |  |  |  |  |  | 2 |
| **DCAF12** DDB1 And CUL4 Associated Factor 12 | 224789_at | (D) DE/6  86% | 8 | NS 0.2 Stepwise |  |  | (D) SH-SY5Y cells Cocaine [^268^](#_ENREF_268)  (D) Blood Universal Suicide[^120^](#_ENREF_120) |  |  |  | 2 |
| **SHMT1**  Serine Hydroxymethyltransferase 1 | 217304_at | (D)  DE/2  43% | 8 | NS |  |  | (D) Blood Suicide  [^129^](#_ENREF_129),[^120^](#_ENREF_120) |  |  |  | 2 |
| **(Hs.596713)**  **PPP1R14B**  Protein Phosphatase 1 Regulatory Inhibitor Subunit 14B | 226138_s_at | (D) DE/6  90% | 6 | 6.28E-02  Stepwise |  |  |  |  | (D) parietal cortex SZ[^273^](#_ENREF_273) |  | 2 |
| **CCDC85C** Coiled-Coil Domain Containing 85C | 219018_s_at | (D) DE/6  94% | 6 | NS |  |  | (D) Male Blood Suicide [^129^](#_ENREF_129) |  |  |  | 2 |
| **CLSPN** Claspin | 242150_at | (I) AP/6  95% | 6 | NS |  |  | (I) Blood Suicide [^119^](#_ENREF_119)^,^ [^129^](#_ENREF_129) |  |  |  | 2 |
| **ELAC2**  **ElaC Ribonuclease Z 2** | 201766_at | (D)  DE/4  52% | 6 | ***4.11E-02/4***  ***Nominal*** | Autism[^274^](#_ENREF_274) |  |  |  |  |  | 2 |
| **Hs.554262** | 210703_at | (I) AP/6 | 6 | NS |  |  | (I) Blood Universal Suicide[^120^](#_ENREF_120) |  |  |  | 2 |
| **(Hs.659426)PHC3**  Polyhomeotic Homolog 3 | 240599_x_at | (D) DE/6 | 6 | NS |  |  | (D) Blood Female Suicide [^119^](#_ENREF_119) |  |  |  | 2 |
| **LY9** Lymphocyte Antigen 9 | 231124_x_at | (I) DE/6  90% | 6 | NS |  |  | (D) Blood Stress[^275^](#_ENREF_275) |  |  |  | 2 |
| **MBNL3** Muscleblind Like Splicing Regulator 3 | 219814_at | (D) DE/6  92% | 6 | NS |  |  | (D) Blood Hallucinations [^147^](#_ENREF_147) |  |  |  | 2 |
| **RALGAPA2**  Ral GTPase Activating Protein Catalytic Alpha Subunit 2 | 231826_at | (D) DE/6  97% | 6 | NS | BP [^70^](#_ENREF_70) |  |  |  |  |  | 2 |
| **SEPT7P2** Septin 7 Pseudogene 2 | 1569973_at | **(I) DE/6**  **100%**  (I)  AP/2  39% | 6 | NS |  |  | (I) Blood Suicide[^119^](#_ENREF_119) |  |  |  | 2 |
| **TCF15** Transcription Factor 15 (Basic Helix-Loop-Helix) | 207306_at | (D) DE/6  94% | 6 | NS |  |  | (D) Blood Suicide [^129^](#_ENREF_129), [^120^](#_ENREF_120) |  |  |  | 2 |
| **TNFRSF11B**  **TNF Receptor Superfamily Member 11b** | 204932_at | (D)  DE/2  37% | 4 | ***2.67E-02/4***  ***Nominal*** |  |  |  |  | (D) Hippocampus Stress[^121^](#_ENREF_121)  (D) PFC Stress [^253^](#_ENREF_253)  (D) HC PTSD [^110^](#_ENREF_110) |  | 2 |
| **HLA-DRB1**  Major Histocompatibility Complex, Class II, DR Beta 1 | 208306_x_at | (I)  AP/4  52% |  | NS |  |  | (I) leukocytes Stress [^262^](#_ENREF_262)  (I) Blood PTSD [^276^](#_ENREF_276) |  |  |  | 2 |
| **CCDC144B**  Coiled-Coil Domain Containing 144B (Pseudogene) | 1557366_at | (D)  DE/4  56% | 10 | NS |  |  |  |  |  |  | 0 |
| **COL2A1**  Collagen Type II Alpha 1 Chain | 217404_s_at | (D) DE/4  54% | 7 | NS |  |  |  |  |  |  | 0 |
| **(AF090920)**  **PPFIBP2**  PPFIA Binding Protein 2 | 234739_at | (I) AP/6  94% | 6 | NS |  |  |  |  |  |  | 0 |
| **DENND1B** DENN Domain Containing 1B | 1557309_at | (I) DE/6  90% | 6 | NS |  |  |  |  |  |  | 0 |
| **ZNF441** Zinc Finger Protein 441 | 1553193_at | (I) AP/6  95%  (I)  DE/2  35% | 6 | NS |  |  |  |  |  |  | 0 |
| **TOP3A**  Topoisomerase (DNA) III Alpha | 214300_s_at | (D)  DE/4  51% | 4 | NS |  |  |  |  |  |  | 0 |
| **ZNF429**  Zinc Finger Protein 429 | 1561270_at | (D)  DE/2  37% | 2 | NS |  |  |  |  |  |  | 0 |

|  | **DAVID GO Functional Annotation Biological Processes** | | | | | **KEGG Pathways** | | | | **Ingenuity Pathways (Fold change)** | | |
| --- | --- | --- | --- | --- | --- | --- | --- | --- | --- | --- | --- | --- |
| A. | # | **Term** | **Count** | **%** | **P-Value** | **Term** | **Count** | **%** | **P-Value** | **Top Canonical Pathways** | **P-Value** | **Overlap** |
| **60 Pain Genes**  **(n= 60**  **Genes,65 probesets)** | 1 | regulation of homeostatic process | 11 | 18.6 | 1.10E-06 | Focal adhesion | 7 | 11.9 | 7.20E-05 | Hereditary Breast Cancer Signaling | 3.36E-05 | 3.5 % 5/144 |
|  | 2 | epithelial cell proliferation | 8 | 13.6 | 9.60E-05 | PI3K-Akt signaling pathway | 8 | 13.6 | 1.60E-04 | Ovarian Cancer Signaling | 3.36E-05 | 3.5 % 5/144 |
|  | 3 | T cell receptor signaling pathway | 6 | 10.2 | 1.70E-04 | Non-small cell lung cancer | 4 | 6.8 | 1.00E-03 | Non-Small Cell Lung Cancer Signaling | 4.53E-05 | 5.2 % 4/77 |
|  | 4 | aging | 7 | 11.9 | 2.30E-04 | Pancreatic cancer | 4 | 6.8 | 1.60E-03 | Glioblastoma Multiform Signaling | 5.89E-05 | 3.1 % 5/162 |
|  | 5 | negative regulation of multicellular organismal process | 12 | 20.3 | 2.50E-04 | Glioma | 4 | 6.8 | 1.60E-03 | HER-2 Signaling in Breast Cancer | 7.65E-05 | 4.5 % 4/88 |

**Table S4 Biological Pathway Analysis:**

| B. | **David** | | | | | **Ingenuity Pathways Disease** | | |
| --- | --- | --- | --- | --- | --- | --- | --- | --- |
| **60 Pain Genes**  **(n= 60**  **Genes,65 probesets)** | # | **Term** | **Count** | **%** | **P-Value** | **Diseases and Disorders** | **P-Value** | **# Molecules** |
|  | 1 | mood disorders | 5 | 8.5 | 2.00E-05 | Neurological Disease | 2.50E-03 - 3.26E-08 | 30 |
|  | 2 | head and neck cancer | 6 | 10.2 | 2.10E-05 | Cancer | 2.50E-03 - 9.87E-08 | 54 |
|  | 3 | Arthritis, Rheumatoid\|Rheumatoid Arthritis | 7 | 11.9 | 4.40E-05 | Organismal Injury and Abnormalities | 2.56E-03 - 9.87E-08 | 55 |
|  | 4 | autism | 9 | 15.3 | 4.40E-05 | Reproductive System Disease | 1.86E-03 - 1.79E-07 | 37 |
|  | 5 | Glomerulonephritis, IGA | 6 | 10.2 | 6.30E-05 | Renal and Urological Disease | 1.44E-03 - 1.11E-06 | 16 |

**Table S5. Pharmacogenomics**. Top list biomarkers in our datasets that are targets of existing drugs and are modulated by them in opposite direction.

| **Gene Symbol/Gene Name Name** | **Probeset** | **Discovery (Change) Method/Score** | **Prioritization Total CFG Score For Pain** | **Validation Anova p-value** | **Pain**  **Medications** | **Omega-3** | **Antidepressants** | **Mood Stabilizers** | **Antipsychotics** | **Others** |
| --- | --- | --- | --- | --- | --- | --- | --- | --- | --- | --- |
| **CNTN1**  Contactin 1 | 1554784_at | (D) DE/4  52% | 10 | NS |  |  |  |  | (I) VT Clozapine  [^156^](#_ENREF_156) |  |
| **GNG7**  G Protein Subunit Gamma 7 | 1566643_a_at | (D)  DE/4  59% | 10 | 6.81E-02/2  Stepwise |  | (I)Brain Omega-3 fatty acids[^277^](#_ENREF_277)  (I)AMY(females) Omega-3 fatty[^278^](#_ENREF_278) |  |  |  |  |
| **ASTN2** Astrotactin 2 | 1554816_at | (I) DE/6  83% | 8 | 1.71E-01  Stepwise |  |  |  |  | Antipsychotics [^279^](#_ENREF_279) |  |
| **CDK6**  Cyclin Dependent Kinase 6 | 224851_at | (I)  DE/4  56%  (I)  AP/2  42% | 8 | NS |  |  |  |  |  | palbociclib, ribociclib, abemaciclib, letrozole/palbociclib, FLX925, fulvestrant/palbociclib, trilaciclib, G1T38, letrozole/ribociclib, abemaciclib/fulvestrant, alvocidib |
| **CDK6**  Cyclin Dependent Kinase 6 | 224847_at | (I)  DE/4  63% | 8 | NS |  |  |  |  |  |  |
| **COL27A1**  Collagen Type XXVII Alpha 1 Chain | 225293_at | (D)  DE/4  79% | 8 | 7.47E-01/2  Stepwise |  |  |  | (I)  AMY Lithium [^280^](#_ENREF_280) |  |  |
| **COMT** Catechol-O-Methyltransferase | 213981_at; 216204_at | (D) DE/4  54% | 8 | NS | Morphine [^41^](#_ENREF_41)  Thermal [^218^](#_ENREF_218) |  |  | Mood Stabilizers[^281^](#_ENREF_281) | (I) VT Clozapine[^156^](#_ENREF_156) |  |
| **DCAF12** DDB1 And CUL4 Associated Factor 12 | 224789_at | (D) DE/6  86% | 8 | NS wise |  | (I) Lymphocytes (females) Omega-3 fatty acids[^278^](#_ENREF_278) |  |  | (I) Lymphocytes Clozapine[^156^](#_ENREF_156) |  |
| **FAM134B** Family With Sequence Similarity 134 Member B | 218510_x_at | (I) DE/4  51%; (I) AP/2  34% | 8 | NS |  | (D) Lymphocytes (females) Omega-3 fatty acids[^278^](#_ENREF_278) |  |  |  |  |
| **GBP 1**  Guanylate Binding Protein 1 | 231578_at | (I)  DE/2  37% | 8 | 3.26E-01/2  Stepwise |  | (D) Blood  Omega-3 fatty acids  [^278^](#_ENREF_278) |  |  |  |  |
| **HLA-DQB1**  Major Histocompatibility Complex, Class II, DQ Beta 1 | 210747_at | (D)  DE/2  44% | 8 | NS |  |  |  |  | (I)BloodBenzodiazepines  [^282^](#_ENREF_282) |  |
| **HLA-DQB1** Major Histocompatibility Complex, Class II, DQ Beta 1 | 211654_x_at | (I)  DE/2  40% | 8 | NS |  |  |  |  | (D)PFC Antipsychotics [^283^](#_ENREF_283) |  |
| **HLA-DQB1** Major Histocompatibility Complex, Class II, DQ Beta 1 | 211656_x_at; 212998_x_at | (I) DE/4  59% | 8 | NS |  |  |  |  | (D) PFC Antipsychotics [^283^](#_ENREF_283) |  |
| **HLA-DRB1**  Major Histocompatibility Complex, Class II, DR Beta 1 | 208306_x_at | (I)  AP/4  52% | 8 | NS |  |  |  |  | (D)PFC Antipsychotics  [^283^](#_ENREF_283) | apolizumab |
| **HTR2A** 5-Hydroxytryptamine Receptor 2A | 211616_s_at | (D) DE/4  52% | 8 | NS |  |  |  |  |  | Hallucinogens |
| **NF1**  Neurofibromin 1 | 212676_at | (I)  DE/4  59% | 8 | NS |  |  | (D) cerebral cortex Fluoxetine SSRI [^284^](#_ENREF_284) |  |  |  |
| **SHMT1**  Serine Hydroxymethyltransferase 1 | 217304_at | (D)  DE/2  43% | 8 | NS |  |  |  |  | (I)VT  Clozapine [^156^](#_ENREF_156) |  |
| **TOP3A**  Topoisomerase (DNA) III Alpha | 214300_s_at | (D)  DE/4  51% | 8 | NS |  | (I)Brain Omega-3 fatty acids[^277^](#_ENREF_277) |  |  |  |  |
| **VEGFA**  Vascular Endothelial Growth Factor A | 212171_x_at | (I)  AP/4  65% | 8 | NS |  |  |  | (D) lymphoblastoid cell cultures Lithium, Valproate[^285^](#_ENREF_285) | (D) HIP and cerebellum  Olanzapine  [^286^](#_ENREF_286) | Anti-cancer mABs |
| **WNK1** WNK Lysine Deficient Protein Kinase 1 | 1555068_at | (D) DE/6  92% | 8 | NS |  | (I) Lymphocytes (females) Omega-3 fatty acids[^278^](#_ENREF_278) | (I) cingulate cortex SSRI (Fluoxetine)[^264^](#_ENREF_264) |  |  |  |
| **CALCA** Calcitonin Related Polypeptide Alpha | 210727_at | (D) DE/4  54% | 7 | NS |  | (I) HIP (males) Omega-3 fatty acids[^278^](#_ENREF_278) |  | (I) Schneider 2 cells Lithium[^287^](#_ENREF_287) |  |  |
| **ZYX**  Zyxin | 238016_s_at | (D) DE/4  57% | 7 | NS |  |  |  |  | (I) Lymphocytes  Clozapine [^156^](#_ENREF_156) |  |
| **(H05785)**  **LRRC75A**  Leucine Rich Repeat Containing 75A | 236913_at | (D) AP/6  97% | 6 | NS |  |  |  |  | (I) HIP Clozapine[^156^](#_ENREF_156) |  |
| **(Hs.596713)**  **PPP1R14B**  Protein Phosphatase 1 Regulatory Inhibitor Subunit 14B | 226138_s_at | (D) DE/6  90% | 6 | 6.28E-02 Stepwise |  |  |  | (I) Schneider 2 (S2) cells, Lithium [^287^](#_ENREF_287) |  |  |
| **(Hs.609761)**  **SFPQ**  Splicing Factor Proline And Glutamine Rich | 244331_at | (D) DE/6  98% | 6 | NS |  | (I) HIP (males) Mood, Omega-3 fatty acids[^278^](#_ENREF_278) | (I) basal forebrain TCA[^288^](#_ENREF_288) |  | (I) PFC Clozapine[^156^](#_ENREF_156) |  |
| **DENND1B** DENN Domain Containing 1B | 1557309_at | (I) DE/6  90%; (I)  AP/2  40% | 6 | NS |  | (D) Brain Omega-3 fatty acids[^277^](#_ENREF_277) |  |  |  |  |
| **GSPT1** G1 To S Phase Transition 1 | 215438_x_at | (D) DE/6  94% | 6 | NS |  |  |  | (I) CP Valproate[^289^](#_ENREF_289) |  |  |
| **HRAS** HRas Proto-Oncogene, GTPase | 212983_at | (I) DE/6  97% | 6 | NS |  |  |  |  |  | ISIS 2503 |
| **LY9** Lymphocyte Antigen 9 | 231124_x_at | (I) DE/6  90% | 6 | NS |  | (D) Brain Omega-3 fatty acids [^277^](#_ENREF_277) |  |  |  |  |
| **PIK3CD** **Phosphatidylinositol-4,5-Bisphosphate 3-Kinase Catalytic Subunit Delta** | 211230_s_at | (D) DE/6  83% | 6 | ***1.59E-02/4***  ***Nominal*** |  |  |  | (I) Lymphoblastoid cells Lithium, Valproate[^285^](#_ENREF_285) | (I) VT Clozapine[^156^](#_ENREF_156) |  |
| **PTN** Pleiotrophin | 211737_x_at | (D) DE/6  92% | 6 | NS |  | (I) HIP (males) Omega-3 fatty acids [^278^](#_ENREF_278)  (I) fronto-temporo-parietal cortex Antipsychotics(risperidone) [^290^](#_ENREF_290) |  |  |  |  |
| **SVEP1**  **Sushi, Von Willebrand Factor Type A, EGF And Pentraxin Domain Containing 1** | 236927_at | (I)  DE/2  49% | 6 | ***2.17E-02/4***  ***Nominal*** |  | (D)Brain Omega-3 fatty acids[^277^](#_ENREF_277) |  |  |  |  |
| **TSPO**  Translocator Protein | 202096_s_at | (I)  DE/2  38% | 6 | NS |  |  |  |  |  | CGS-8216, dexamethasone/olanzapine, fluoxetine/olanzapine, estazolam, clorazepate, eszopiclone, temazepam, zolpidem, chlordiazepoxide, lorazepam, olanzapine, triazolam, flumazenil, clonazepam, flurazepam, midazolam, flunitrazepam, alprazolam, zaleplon, SSR180575, PK 11195 |
| **YBX3** Y-Box Binding Protein 3 | 201160_s_at | (D) DE/6  94% | 6 | NS |  |  | (I) c.elegans mianserin [^291^](#_ENREF_291) |  |  |  |

**Literature Cited:**

1. Parisien M, Khoury S, Chabot-Dore AJ, Sotocinal SG, Slade GD, Smith SB *et al.* Effect of Human Genetic Variability on Gene Expression in Dorsal Root Ganglia and Association with Pain Phenotypes. *Cell reports* 2017; **19**(9)**:** 1940-1952.

2. Jin EH, Zhang E, Ko Y, Sim WS, Moon DE, Yoon KJ *et al.* Genome-wide expression profiling of complex regional pain syndrome. *PloS one* 2013; **8**(11)**:** e79435.

3. Guo Y, Yao F, Lu S, Cao DY, Reed WR, Zhao Y. The major histocompatibility complex genes are associated with basal pain sensitivity differences between Dark-Agouti and novel congenic DA.1U rats. *Life Sci* 2010; **86**(25-26)**:** 972-978.

4. Yi Y, Zhao M, Xu F, Liu C, Yin Y, Yu J. CGRP 4218T/C polymorphism correlated with postoperative analgesic effect of fentanyl. *International journal of clinical and experimental pathology* 2015; **8**(5)**:** 5761-5767.

5. Sutherland HG, Buteri J, Menon S, Haupt LM, Macgregor EA, Lea RA *et al.* Association study of the calcitonin gene-related polypeptide-alpha (CALCA) and the receptor activity modifying 1 (RAMP1) genes with migraine. *Gene* 2013; **515**(1)**:** 187-192.

6. Gruber HE, Hoelscher GL, Ingram JA, Hanley EN, Jr. Genome-wide analysis of pain-, nerve- and neurotrophin -related gene expression in the degenerating human annulus. *Molecular pain* 2012; **8:** 63.

7. Starkweather AR, Ramesh D, Lyon DE, Siangphoe U, Deng X, Sturgill J *et al.* Acute Low Back Pain: Differential Somatosensory Function and Gene Expression Compared With Healthy No-Pain Controls. *Clin J Pain* 2016; **32**(11)**:** 933-939.

8. Guo S. The role of genetics on migraine induction triggered by CGRP and PACAP38. *Dan Med J* 2017; **64**(3).

9. Lu CL, Pasricha PJ, Hsieh JC, Lu RH, Lai CR, Wu LL *et al.* Changes of the neuropeptides content and gene expression in spinal cord and dorsal root ganglion after noxious colorectal distension. *Regulatory peptides* 2005; **131**(1-3)**:** 66-73.

10. LaCroix-Fralish ML, Austin JS, Zheng FY, Levitin DJ, Mogil JS. Patterns of pain: meta-analysis of microarray studies of pain. *Pain* 2011; **152**(8)**:** 1888-1898.

11. Kimura S, Sakuma Y, Suzuki M, Orita S, Yamauchi K, Inoue G *et al.* Evaluation of pain behavior and calcitonin gene-related peptide immunoreactive sensory nerve fibers in the spinal dorsal horn after sciatic nerve compression and application of nucleus pulposus in rats. *Spine (Phila Pa 1976)* 2014; **39**(6)**:** 455-462.

12. Sugawara S, Okada S, Katagiri A, Saito H, Suzuki T, Komiya H *et al.* Interaction between calcitonin gene-related peptide-immunoreactive neurons and satellite cells via P2Y12 R in the trigeminal ganglion is involved in neuropathic tongue pain in rats. *European journal of oral sciences* 2017; **125**(6)**:** 444-452.

13. Descalzi G, Mitsi V, Purushothaman I, Gaspari S, Avrampou K, Loh YE *et al.* Neuropathic pain promotes adaptive changes in gene expression in brain networks involved in stress and depression. *Science signaling* 2017; **10**(471).

14. Miura Y, Devaux JJ, Fukami Y, Manso C, Belghazi M, Wong AH *et al.* Contactin 1 IgG4 associates to chronic inflammatory demyelinating polyneuropathy with sensory ataxia. *Brain : a journal of neurology* 2015; **138**(Pt 6)**:** 1484-1491.

15. Olausson P, Ghafouri B, Backryd E, Gerdle B. Clear differences in cerebrospinal fluid proteome between women with chronic widespread pain and healthy women - a multivariate explorative cross-sectional study. *Journal of pain research* 2017; **10:** 575-590.

16. Hur J, Sullivan KA, Pande M, Hong Y, Sima AA, Jagadish HV *et al.* The identification of gene expression profiles associated with progression of human diabetic neuropathy. *Brain : a journal of neurology* 2011; **134**(Pt 11)**:** 3222-3235.

17. Freilinger T, Anttila V, de Vries B, Malik R, Kallela M, Terwindt GM *et al.* Genome-wide association analysis identifies susceptibility loci for migraine without aura. *Nature genetics* 2012; **44**(7)**:** 777-782.

18. Anttila V, Winsvold BS, Gormley P, Kurth T, Bettella F, McMahon G *et al.* Genome-wide meta-analysis identifies new susceptibility loci for migraine. *Nature genetics* 2013; **45**(8)**:** 912-917.

19. Gormley P, Anttila V, Winsvold BS, Palta P, Esko T, Pers TH *et al.* Meta-analysis of 375,000 individuals identifies 38 susceptibility loci for migraine. *Nature genetics* 2016; **48**(8)**:** 856-866.

20. Esserlind AL, Christensen AF, Steinberg S, Grarup N, Pedersen O, Hansen T *et al.* The association between candidate migraine susceptibility loci and severe migraine phenotype in a clinical sample. *Cephalalgia : an international journal of headache* 2016; **36**(7)**:** 615-623.

21. Berta T, Perrin FE, Pertin M, Tonello R, Liu YC, Chamessian A *et al.* Gene Expression Profiling of Cutaneous Injured and Non-Injured Nociceptors in SNI Animal Model of Neuropathic Pain. *Scientific reports* 2017; **7**(1)**:** 9367.

22. McDonald MK, Tian Y, Qureshi RA, Gormley M, Ertel A, Gao R *et al.* Functional significance of macrophage-derived exosomes in inflammation and pain. *Pain* 2014; **155**(8)**:** 1527-1539.

23. Chen CJ, Liu DZ, Yao WF, Gu Y, Huang F, Hei ZQ *et al.* Identification of key genes and pathways associated with neuropathic pain in uninjured dorsal root ganglion by using bioinformatic analysis. *Journal of pain research* 2017; **10:** 2665-2674.

24. Nagata E, Hattori H, Kato M, Ogasawara S, Suzuki S, Shibata M *et al.* Identification of biomarkers associated with migraine with aura. *Neuroscience research* 2009; **64**(1)**:** 104-110.

25. Zubieta JK, Heitzeg MM, Smith YR, Bueller JA, Xu K, Xu Y *et al.* COMT val158met genotype affects mu-opioid neurotransmitter responses to a pain stressor. *Science* 2003; **299**(5610)**:** 1240-1243.

26. Sadhasivam S, Chidambaran V, Olbrecht VA, Esslinger HR, Zhang K, Zhang X *et al.* Genetics of pain perception, COMT and postoperative pain management in children. *Pharmacogenomics* 2014; **15**(3)**:** 277-284.

27. Jacobsen LM, Schistad EI, Storesund A, Pedersen LM, Rygh LJ, Roe C *et al.* The COMT rs4680 Met allele contributes to long-lasting low back pain, sciatica and disability after lumbar disc herniation. *European journal of pain* 2012; **16**(7)**:** 1064-1069.

28. Omair A, Lie BA, Reikeras O, Holden M, Brox JI. Genetic contribution of catechol-O-methyltransferase variants in treatment outcome of low back pain: a prospective genetic association study. *BMC musculoskeletal disorders* 2012; **13:** 76.

29. Omair A, Mannion AF, Holden M, Fairbank J, Lie BA, Hagg O *et al.* Catechol-O-methyltransferase (COMT) gene polymorphisms are associated with baseline disability but not long-term treatment outcome in patients with chronic low back pain. *European spine journal : official publication of the European Spine Society, the European Spinal Deformity Society, and the European Section of the Cervical Spine Research Society* 2015; **24**(11)**:** 2425-2431.

30. Smith SB, Reenila I, Mannisto PT, Slade GD, Maixner W, Diatchenko L *et al.* Epistasis between polymorphisms in COMT, ESR1, and GCH1 influences COMT enzyme activity and pain. *Pain* 2014; **155**(11)**:** 2390-2399.

31. Smith SB, Maixner DW, Greenspan JD, Dubner R, Fillingim RB, Ohrbach R *et al.* Potential genetic risk factors for chronic TMD: genetic associations from the OPPERA case control study. *J Pain* 2011; **12**(11 Suppl)**:** T92-101.

32. Vargas-Alarcon G, Fragoso JM, Cruz-Robles D, Vargas A, Vargas A, Lao-Villadoniga JI *et al.* Catechol-O-methyltransferase gene haplotypes in Mexican and Spanish patients with fibromyalgia. *Arthritis Res Ther* 2007; **9**(5)**:** R110.

33. Fernandez-de-Las-Penas C, Ambite-Quesada S, Gil-Crujera A, Cigaran-Mendez M, Penacoba-Puente C. Catechol-O-methyltransferase Val158Met polymorphism influences anxiety, depression, and disability, but not pressure pain sensitivity, in women with fibromyalgia syndrome. *J Pain* 2012; **13**(11)**:** 1068-1074.

34. Martinez-Jauand M, Sitges C, Rodriguez V, Picornell A, Ramon M, Buskila D *et al.* Pain sensitivity in fibromyalgia is associated with catechol-O-methyltransferase (COMT) gene. *Eur J Pain* 2013; **17**(1)**:** 16-27.

35. Barbosa FR, Matsuda JB, Mazucato M, de Castro Franca S, Zingaretti SM, da Silva LM *et al.* Influence of catechol-O-methyltransferase (COMT) gene polymorphisms in pain sensibility of Brazilian fibromialgia patients. *Rheumatol Int* 2012; **32**(2)**:** 427-430.

36. Cohen H, Neumann L, Glazer Y, Ebstein RP, Buskila D. The relationship between a common catechol-O-methyltransferase (COMT) polymorphism val(158) met and fibromyalgia. *Clin Exp Rheumatol* 2009; **27**(5 Suppl 56)**:** S51-56.

37. Finan PH, Zautra AJ, Davis MC, Lemery-Chalfant K, Covault J, Tennen H. COMT moderates the relation of daily maladaptive coping and pain in fibromyalgia. *Pain* 2011; **152**(2)**:** 300-307.

38. Kim H, Mittal DP, Iadarola MJ, Dionne RA. Genetic predictors for acute experimental cold and heat pain sensitivity in humans. *J Med Genet* 2006; **43**(8)**:** e40.

39. Nielsen LM, Christrup LL, Sato H, Drewes AM, Olesen AE. Genetic Influences of OPRM1, OPRD1 and COMT on Morphine Analgesia in a Multi-Modal, Multi-Tissue Human Experimental Pain Model. *Basic & clinical pharmacology & toxicology* 2017; **121**(1)**:** 6-12.

40. James S. Human pain and genetics: some basics. *British journal of pain* 2013; **7**(4)**:** 171-178.

41. Rakvag TT, Ross JR, Sato H, Skorpen F, Kaasa S, Klepstad P. Genetic variation in the catechol-O-methyltransferase (COMT) gene and morphine requirements in cancer patients with pain. *Molecular pain* 2008; **4:** 64.

42. Rut M, Machoy-Mokrzynska A, Reclawowicz D, Sloniewski P, Kurzawski M, Drozdzik M *et al.* Influence of variation in the catechol-O-methyltransferase gene on the clinical outcome after lumbar spine surgery for one-level symptomatic disc disease: a report on 176 cases. *Acta Neurochir (Wien)* 2014; **156**(2)**:** 245-252.

43. Nah SS, Lee H, Hong Y, Im J, Won H, Chang SH *et al.* Association between endothelin1 and fibromyalgia syndrome. *Molecular medicine reports* 2017; **16**(5)**:** 6234-6239.

44. Fisher JA, Favreau MB. Plasmid purification by phenol extraction from guanidinium thiocyanate solution: development of an automated protocol. *Analytical biochemistry* 1991; **194**(2)**:** 309-315.

45. Murphy SM, Davidson GL, Brandner S, Houlden H, Reilly MM. Mutation in FAM134B causing severe hereditary sensory neuropathy. *J Neurol Neurosurg Psychiatry* 2012; **83**(1)**:** 119-120.

46. Smith SB, Maixner DW, Fillingim RB, Slade G, Gracely RH, Ambrose K *et al.* Large candidate gene association study reveals genetic risk factors and therapeutic targets for fibromyalgia. *Arthritis and rheumatism* 2012; **64**(2)**:** 584-593.

47. Rainero I, Fasano E, Rubino E, Rivoiro C, Valfre W, Gallone S *et al.* Association between migraine and HLA-DRB1 gene polymorphisms. *The journal of headache and pain* 2005; **6**(4)**:** 185-187.

48. Lebe M, Hasenbring MI, Schmieder K, Jetschke K, Harders A, Epplen JT *et al.* Association of serotonin-1A and -2A receptor promoter polymorphisms with depressive symptoms, functional recovery, and pain in patients 6 months after lumbar disc surgery. *Pain* 2013; **154**(3)**:** 377-384.

49. Nicholl BI, Holliday KL, Macfarlane GJ, Thomson W, Davies KA, O'Neill TW *et al.* Association of HTR2A polymorphisms with chronic widespread pain and the extent of musculoskeletal pain: results from two population-based cohorts. *Arthritis and rheumatism* 2011; **63**(3)**:** 810-818.

50. Slade GD, Smith SB, Zaykin DV, Tchivileva IE, Gibson DG, Yuryev A *et al.* Facial pain with localized and widespread manifestations: separate pathways of vulnerability. *Pain* 2013; **154**(11)**:** 2335-2343.

51. Gursoy S, Erdal E, Herken H, Madenci E, Alasehirli B. Association of T102C polymorphism of the 5-HT2A receptor gene with psychiatric status in fibromyalgia syndrome. *Rheumatology international* 2001; **21**(2)**:** 58-61.

52. Holliday KL, Nicholl BI, Macfarlane GJ, Thomson W, Davies KA, McBeth J. Genetic variation in the hypothalamic-pituitary-adrenal stress axis influences susceptibility to musculoskeletal pain: results from the EPIFUND study. *Annals of the rheumatic diseases* 2010; **69**(3)**:** 556-560.

53. Mergener M, Becker RM, dos Santos AF, dos Santos GA, de Andrade FM. Influence of the interaction between environmental quality and T102C SNP in the HTR2A gene on fibromyalgia susceptibility. *Rev Bras Reumatol* 2011; **51**(6)**:** 594-602.

54. Aoki J, Hayashida M, Tagami M, Nagashima M, Fukuda K, Nishizawa D *et al.* Association between 5-hydroxytryptamine 2A receptor gene polymorphism and postoperative analgesic requirements after major abdominal surgery. *Neuroscience letters* 2010; **479**(1)**:** 40-43.

55. Heddini U, Bohm-Starke N, Gronbladh A, Nyberg F, Nilsson KW, Johannesson U. Serotonin receptor gene (5HT-2A) polymorphism is associated with provoked vestibulodynia and comorbid symptoms of pain. *The journal of sexual medicine* 2014; **11**(12)**:** 3064-3071.

56. Cox HC, Lea RA, Bellis C, Carless M, Dyer TD, Curran J *et al.* A genome-wide analysis of 'Bounty' descendants implicates several novel variants in migraine susceptibility. *Neurogenetics* 2012; **13**(3)**:** 261-266.

57. Aneiros-Guerrero A, Lendinez AM, Palomares AR, Perez-Nevot B, Aguado L, Mayor-Olea A *et al.* Genetic polymorphisms in folate pathway enzymes, DRD4 and GSTM1 are related to temporomandibular disorder. *BMC medical genetics* 2011; **12:** 75.

58. Loggia ML, Chonde DB, Akeju O, Arabasz G, Catana C, Edwards RR *et al.* Evidence for brain glial activation in chronic pain patients. *Brain : a journal of neurology* 2015; **138**(Pt 3)**:** 604-615.

59. Han IB, Ropper AE, Teng YD, Shin DA, Jeon YJ, Park HM *et al.* Association between VEGF and eNOS gene polymorphisms and lumbar disc degeneration in a young Korean population. *Genetics and molecular research : GMR* 2013; **12**(3)**:** 2294-2305.

60. Rosenbaum RS, Kwan D, Floden D, Levine B, Stuss DT, Craver CF. No evidence of risk-taking or impulsive behaviour in a person with episodic amnesia: Implications for the role of the hippocampus in future-regarding decision-making. *Quarterly journal of experimental psychology* 2016; **69**(8)**:** 1606-1618.

61. Dagher A, Curatolo A, Sachdev M, Stephens AJ, Mullins C, Landis JR *et al.* Identification of novel non-invasive biomarkers of urinary chronic pelvic pain syndrome: findings from the Multidisciplinary Approach to the Study of Chronic Pelvic Pain (MAPP) Research Network. *BJU international* 2017; **120**(1)**:** 130-142.

62. Grosman-Rimon L, Parkinson W, Upadhye S, Clarke H, Katz J, Flannery J *et al.* Circulating biomarkers in acute myofascial pain: A case-control study. *Medicine* 2016; **95**(37)**:** e4650.

63. Rotthier A, Baets J, De Vriendt E, Jacobs A, Auer-Grumbach M, Levy N *et al.* Genes for hereditary sensory and autonomic neuropathies: a genotype-phenotype correlation. *Brain : a journal of neurology* 2009; **132**(Pt 10)**:** 2699-2711.

64. Kette F, Weil MH, Gazmuri RJ. Buffer solutions may compromise cardiac resuscitation by reducing coronary perfusion presssure. *Jama* 1991; **266**(15)**:** 2121-2126.

65. Kim H, Ramsay E, Lee H, Wahl S, Dionne RA. Genome-wide association study of acute post-surgical pain in humans. *Pharmacogenomics* 2009; **10**(2)**:** 171-179.

66. Reeser JC, Payne E, Kitchner T, McCarty CA. Apolipoprotein e4 genotype increases the risk of being diagnosed with posttraumatic fibromyalgia. *PM & R : the journal of injury, function, and rehabilitation* 2011; **3**(3)**:** 193-197.

67. Lintermans A, Van Asten K, Jongen L, Van Brussel T, Laenen A, Verhaeghe J *et al.* Genetic variant in the osteoprotegerin gene is associated with aromatase inhibitor-related musculoskeletal toxicity in breast cancer patients. *European journal of cancer* 2016; **56:** 31-36.

68. Kramer HH, Hofbauer LC, Szalay G, Breimhorst M, Eberle T, Zieschang K *et al.* Osteoprotegerin: a new biomarker for impaired bone metabolism in complex regional pain syndrome? *Pain* 2014; **155**(5)**:** 889-895.

69. Wrzosek M, Lukaszkiewicz J, Wrzosek M, Serafin P, Jakubczyk A, Klimkiewicz A *et al.* Association of polymorphisms in HTR2A, HTR1A and TPH2 genes with suicide attempts in alcohol dependence: a preliminary report. *Psychiatry research* 2011; **190**(1)**:** 149-151.

70. Nurnberger JI, Jr., Koller DL, Jung J, Edenberg HJ, Foroud T, Guella I *et al.* Identification of pathways for bipolar disorder: a meta-analysis. *JAMA Psychiatry* 2014; **71**(6)**:** 657-664.

71. McAuley EZ, Fullerton JM, Blair IP, Donald JA, Mitchell PB, Schofield PR. Association between the serotonin 2A receptor gene and bipolar affective disorder in an Australian cohort. *Psychiatric genetics* 2009; **19**(5)**:** 244-252.

72. Chee IS, Lee SW, Kim JL, Wang SK, Shin YO, Shin SC *et al.* 5-HT2A receptor gene promoter polymorphism -1438A/G and bipolar disorder. *Psychiatr Genet* 2001; **11**(3)**:** 111-114.

73. Bonnier B, Gorwood P, Hamon M, Sarfati Y, Boni C, Hardy-Bayle MC. Association of 5-HT(2A) receptor gene polymorphism with major affective disorders: the case of a subgroup of bipolar disorder with low suicide risk. *Biol Psychiatry* 2002; **51**(9)**:** 762-765.

74. Lin YM, Yang HC, Lai TJ, Fann CS, Sun HS. Receptor mediated effect of serotonergic transmission in patients with bipolar affective disorder. *Journal of medical genetics* 2003; **40**(10)**:** 781-786.

75. Jansson M, Gatz M, Berg S, Johansson B, Malmberg B, McClearn GE *et al.* Association between depressed mood in the elderly and a 5-HTR2A gene variant. *American journal of medical genetics Part B, Neuropsychiatric genetics : the official publication of the International Society of Psychiatric Genetics* 2003; **120B**(1)**:** 79-84.

76. Luciano M, Houlihan LM, Harris SE, Gow AJ, Hayward C, Starr JM *et al.* Association of existing and new candidate genes for anxiety, depression and personality traits in older people. *Behav Genet* 2010; **40**(4)**:** 518-532.

77. Lewis CM, Ng MY, Butler AW, Cohen-Woods S, Uher R, Pirlo K *et al.* Genome-wide association study of major recurrent depression in the U.K. population. *Am J Psychiatry* 2010; **167**(8)**:** 949-957.

78. Muglia P, Tozzi F, Galwey NW, Francks C, Upmanyu R, Kong XQ *et al.* Genome-wide association study of recurrent major depressive disorder in two European case-control cohorts. *Mol Psychiatry* 2010; **15**(6)**:** 589-601.

79. Brezo J, Bureau A, Merette C, Jomphe V, Barker ED, Vitaro F *et al.* Differences and similarities in the serotonergic diathesis for suicide attempts and mood disorders: a 22-year longitudinal gene-environment study. *Mol Psychiatry* 2010; **15**(8)**:** 831-843.

80. Denys D, Van Nieuwerburgh F, Deforce D, Westenberg HG. Association between serotonergic candidate genes and specific phenotypes of obsessive compulsive disorder. *Journal of affective disorders* 2006; **91**(1)**:** 39-44.

81. Maher BS, Vladimirov VI, Latendresse SJ, Thiselton DL, McNamee R, Kang M *et al.* The AVPR1A gene and substance use disorders: association, replication, and functional evidence. *Biological psychiatry* 2011; **70**(6)**:** 519-527.

82. Maron E, Nikopensius T, Koks S, Altmae S, Heinaste E, Vabrit K *et al.* Association study of 90 candidate gene polymorphisms in panic disorder. *Psychiatric genetics* 2005; **15**(1)**:** 17-24.

83. Unschuld PG, Ising M, Erhardt A, Lucae S, Kloiber S, Kohli M *et al.* Polymorphisms in the serotonin receptor gene HTR2A are associated with quantitative traits in panic disorder. *American journal of medical genetics Part B, Neuropsychiatric genetics : the official publication of the International Society of Psychiatric Genetics* 2007; **144B**(4)**:** 424-429.

84. McMahon FJ, Buervenich S, Charney D, Lipsky R, Rush AJ, Wilson AF *et al.* Variation in the gene encoding the serotonin 2A receptor is associated with outcome of antidepressant treatment. *Am J Hum Genet* 2006; **78**(5)**:** 804-814.

85. Rubin DH, Althoff RR, Ehli EA, Davies GE, Rettew DC, Crehan ET *et al.* Candidate gene associations with withdrawn behavior. *Journal of child psychology and psychiatry, and allied disciplines* 2013; **54**(12)**:** 1337-1345.

86. Ben-Efraim YJ, Wasserman D, Wasserman J, Sokolowski M. Family-based study of HTR2A in suicide attempts: observed gene, gene x environment and parent-of-origin associations. *Molecular psychiatry* 2013; **18**(7)**:** 758-766.

87. Giegling I, Hartmann AM, Moller HJ, Rujescu D. Anger- and aggression-related traits are associated with polymorphisms in the 5-HT-2A gene. *Journal of affective disorders* 2006; **96**(1-2)**:** 75-81.

88. Gonzalez-Castro TB, Tovilla-Zarate C, Juarez-Rojop I, Pool Garcia S, Velazquez-Sanchez MP, Genis A *et al.* Association of the 5HTR2A gene with suicidal behavior: case-control study and updated meta-analysis. *BMC psychiatry* 2013; **13:** 25.

89. Shinozaki G, Romanowicz M, Mrazek DA, Kung S. HTR2A gene-child abuse interaction and association with a history of suicide attempt among Caucasian depressed psychiatric inpatients. *Journal of affective disorders* 2013; **150**(3)**:** 1200-1203.

90. Vaquero-Lorenzo C, Baca-Garcia E, Diaz-Hernandez M, Perez-Rodriguez MM, Fernandez-Navarro P, Giner L *et al.* Association study of two polymorphisms of the serotonin-2A receptor gene and suicide attempts. *Am J Med Genet B Neuropsychiatr Genet* 2008; **147B**(5)**:** 645-649.

91. Knable MB, Barci BM, Webster MJ, Meador-Woodruff J, Torrey EF, Stanley Neuropathology C. Molecular abnormalities of the hippocampus in severe psychiatric illness: postmortem findings from the Stanley Neuropathology Consortium. *Molecular psychiatry* 2004; **9**(6)**:** 609-620, 544.

92. Lopez-Figueroa AL, Norton CS, Lopez-Figueroa MO, Armellini-Dodel D, Burke S, Akil H *et al.* Serotonin 5-HT1A, 5-HT1B, and 5-HT2A receptor mRNA expression in subjects with major depression, bipolar disorder, and schizophrenia. *Biological psychiatry* 2004; **55**(3)**:** 225-233.

93. Polesskaya OO, Sokolov BP. Differential expression of the "C" and "T" alleles of the 5-HT2A receptor gene in the temporal cortex of normal individuals and schizophrenics. *Journal of neuroscience research* 2002; **67**(6)**:** 812-822.

94. Torrey EF, Barci BM, Webster MJ, Bartko JJ, Meador-Woodruff JH, Knable MB. Neurochemical markers for schizophrenia, bipolar disorder, and major depression in postmortem brains. *Biol Psychiatry* 2005; **57**(3)**:** 252-260.

95. Labonte B, Suderman M, Maussion G, Lopez JP, Navarro-Sanchez L, Yerko V *et al.* Genome-wide methylation changes in the brains of suicide completers. *Am J Psychiatry* 2013; **170**(5)**:** 511-520.

96. Erraji-Benchekroun L, Underwood MD, Arango V, Galfalvy H, Pavlidis P, Smyrniotopoulos P *et al.* Molecular aging in human prefrontal cortex is selective and continuous throughout adult life. *Biological psychiatry* 2005; **57**(5)**:** 549-558.

97. van Heeringen C, Audenaert K, Van Laere K, Dumont F, Slegers G, Mertens J *et al.* Prefrontal 5-HT2a receptor binding index, hopelessness and personality characteristics in attempted suicide. *Journal of affective disorders* 2003; **74**(2)**:** 149-158.

98. Klempan TA, Sequeira A, Canetti L, Lalovic A, Ernst C, ffrench-Mullen J *et al.* Altered expression of genes involved in ATP biosynthesis and GABAergic neurotransmission in the ventral prefrontal cortex of suicides with and without major depression. *Molecular psychiatry* 2009; **14**(2)**:** 175-189.

99. Chen H, Wang N, Zhao X, Ross CA, O'Shea KS, McInnis MG. Gene expression alterations in bipolar disorder postmortem brains. *Bipolar disorders* 2013; **15**(2)**:** 177-187.

100. Anisman H, Du L, Palkovits M, Faludi G, Kovacs GG, Szontagh-Kishazi P *et al.* Serotonin receptor subtype and p11 mRNA expression in stress-relevant brain regions of suicide and control subjects. *Journal of psychiatry & neuroscience : JPN* 2008; **33**(2)**:** 131-141.

101. Inada Y, Yoneda H, Koh J, Sakai J, Himei A, Kinoshita Y *et al.* Positive association between panic disorder and polymorphism of the serotonin 2A receptor gene. *Psychiatry research* 2003; **118**(1)**:** 25-31.

102. Sequeira A, Morgan L, Walsh DM, Cartagena PM, Choudary P, Li J *et al.* Gene expression changes in the prefrontal cortex, anterior cingulate cortex and nucleus accumbens of mood disorders subjects that committed suicide. *PloS one* 2012; **7**(4)**:** e35367.

103. Fukuda Y, Koga M, Arai M, Noguchi E, Ohtsuki T, Horiuchi Y *et al.* Monoallelic and unequal allelic expression of the HTR2A gene in human brain and peripheral lymphocytes. *Biological psychiatry* 2006; **60**(12)**:** 1331-1335.

104. Silver H, Susser E, Danovich L, Bilker W, Youdim M, Goldin V *et al.* SSRI augmentation of antipsychotic alters expression of GABA(A) receptor and related genes in PMC of schizophrenia patients. *The international journal of neuropsychopharmacology* 2011; **14**(5)**:** 573-584.

105. Malone KM, Ellis SP, Currier D, John Mann J. Platelet 5-HT2A receptor subresponsivity and lethality of attempted suicide in depressed in-patients. *The international journal of neuropsychopharmacology* 2007; **10**(3)**:** 335-343.

106. Weisstaub NV, Zhou M, Lira A, Lambe E, Gonzalez-Maeso J, Hornung JP *et al.* Cortical 5-HT2A receptor signaling modulates anxiety-like behaviors in mice. *Science* 2006; **313**(5786)**:** 536-540.

107. Ortega-Alvaro A, Aracil-Fernandez A, Garcia-Gutierrez MS, Navarrete F, Manzanares J. Deletion of CB2 cannabinoid receptor induces schizophrenia-related behaviors in mice. *Neuropsychopharmacology* 2011; **36**(7)**:** 1489-1504.

108. Sakata K, Duke SM. Lack of BDNF expression through promoter IV disturbs expression of monoamine genes in the frontal cortex and hippocampus. *Neuroscience* 2014; **260:** 265-275.

109. Steward LJ, Kennedy MD, Morris BJ, Pratt JA. The atypical antipsychotic drug clozapine enhances chronic PCP-induced regulation of prefrontal cortex 5-HT2A receptors. *Neuropharmacology* 2004; **47**(4)**:** 527-537.

110. Muhie S, Gautam A, Meyerhoff J, Chakraborty N, Hammamieh R, Jett M. Brain transcriptome profiles in mouse model simulating features of post-traumatic stress disorder. *Molecular brain* 2015; **8:** 14.

111. Bagot RC, Cates HM, Purushothaman I, Lorsch ZS, Walker DM, Wang J *et al.* Circuit-wide Transcriptional Profiling Reveals Brain Region-Specific Gene Networks Regulating Depression Susceptibility. *Neuron* 2016; **90**(5)**:** 969-983.

112. Zhang EE, Liu AC, Hirota T, Miraglia LJ, Welch G, Pongsawakul PY *et al.* A genome-wide RNAi screen for modifiers of the circadian clock in human cells. *Cell* 2009; **139**(1)**:** 199-210.

113. Sebastiani P, Bae H, Sun FX, Andersen SL, Daw EW, Malovini A *et al.* Meta-analysis of genetic variants associated with human exceptional longevity. *Aging* 2013; **5**(9)**:** 653-661.

114. Sebastiani P, Solovieff N, Dewan AT, Walsh KM, Puca A, Hartley SW *et al.* Genetic signatures of exceptional longevity in humans. *PloS one* 2012; **7**(1)**:** e29848.

115. Kalsi G, Kuo PH, Aliev F, Alexander J, McMichael O, Patterson DG *et al.* A systematic gene-based screen of chr4q22-q32 identifies association of a novel susceptibility gene, DKK2, with the quantitative trait of alcohol dependence symptom counts. *Human molecular genetics* 2010; **19**(12)**:** 2497-2506.

116. Harris LW, Lockstone HE, Khaitovich P, Weickert CS, Webster MJ, Bahn S. Gene expression in the prefrontal cortex during adolescence: implications for the onset of schizophrenia. *BMC Med Genomics* 2009; **2:** 28.

117. Katsel P, Davis KL, Li C, Tan W, Greenstein E, Kleiner Hoffman LB *et al.* Abnormal indices of cell cycle activity in schizophrenia and their potential association with oligodendrocytes. *Neuropsychopharmacology : official publication of the American College of Neuropsychopharmacology* 2008; **33**(12)**:** 2993-3009.

118. Hu VW, Frank BC, Heine S, Lee NH, Quackenbush J. Gene expression profiling of lymphoblastoid cell lines from monozygotic twins discordant in severity of autism reveals differential regulation of neurologically relevant genes. *BMC genomics* 2006; **7:** 118.

119. Levey DF, Niculescu EM, Le-Niculescu H, Dainton HL, Phalen PL, Ladd TB *et al.* Towards understanding and predicting suicidality in women: biomarkers and clinical risk assessment. *Molecular psychiatry* 2016; **21**(6)**:** 768-785.

120. Niculescu AB, Le-Niculescu H, Levey DF, Phalen PL, Dainton HL, Roseberry K *et al.* Precision medicine for suicidality: from universality to subtypes and personalization. *Mol Psychiatry* 2017; **22**(9)**:** 1250-1273.

121. Andrus BM, Blizinsky K, Vedell PT, Dennis K, Shukla PK, Schaffer DJ *et al.* Gene expression patterns in the hippocampus and amygdala of endogenous depression and chronic stress models. *Molecular psychiatry* 2012; **17**(1)**:** 49-61.

122. Akisaka M, Suzuki M, Inoko H. Molecular genetic studies on DNA polymorphism of the HLA class II genes associated with human longevity. *Tissue antigens* 1997; **50**(5)**:** 489-493.

123. Levine ME, Crimmins EM. A Genetic Network Associated With Stress Resistance, Longevity, and Cancer in Humans. *The journals of gerontology Series A, Biological sciences and medical sciences* 2016; **71**(6)**:** 703-712.

124. International Schizophrenia C, Purcell SM, Wray NR, Stone JL, Visscher PM, O'Donovan MC *et al.* Common polygenic variation contributes to risk of schizophrenia and bipolar disorder. *Nature* 2009; **460**(7256)**:** 748-752.

125. Aberg KA, Liu Y, Bukszar J, McClay JL, Khachane AN, Andreassen OA *et al.* A comprehensive family-based replication study of schizophrenia genes. *JAMA psychiatry* 2013; **70**(6)**:** 573-581.

126. Barnes MR, Huxley-Jones J, Maycox PR, Lennon M, Thornber A, Kelly F *et al.* Transcription and pathway analysis of the superior temporal cortex and anterior prefrontal cortex in schizophrenia. *Journal of neuroscience research* 2011; **89**(8)**:** 1218-1227.

127. Glatt SJ, Stone WS, Nossova N, Liew CC, Seidman LJ, Tsuang MT. Similarities and differences in peripheral blood gene-expression signatures of individuals with schizophrenia and their first-degree biological relatives. *Am J Med Genet B Neuropsychiatr Genet* 2011; **156B**(8)**:** 869-887.

128. Le-Niculescu H, Levey DF, Ayalew M, Palmer L, Gavrin LM, Jain N *et al.* Discovery and validation of blood biomarkers for suicidality. *Mol Psychiatry* 2013; **18**(12)**:** 1249-1264.

129. Niculescu AB, Levey DF, Phalen PL, Le-Niculescu H, Dainton HD, Jain N *et al.* Understanding and predicting suicidality using a combined genomic and clinical risk assessment approach. *Molecular psychiatry* 2015; **20**(11)**:** 1266-1285.

130. Miller GE, Chen E, Sze J, Marin T, Arevalo JM, Doll R *et al.* A functional genomic fingerprint of chronic stress in humans: blunted glucocorticoid and increased NF-kappaB signaling. *Biological psychiatry* 2008; **64**(4)**:** 266-272.

131. Segman RH, Shefi N, Goltser-Dubner T, Friedman N, Kaminski N, Shalev AY. Peripheral blood mononuclear cell gene expression profiles identify emergent post-traumatic stress disorder among trauma survivors. *Mol Psychiatry* 2005; **10**(5)**:** 500-513, 425.

132. Mellon SH, Wolkowitz OM, Schonemann MD, Epel ES, Rosser R, Burke HB *et al.* Alterations in leukocyte transcriptional control pathway activity associated with major depressive disorder and antidepressant treatment. *Translational psychiatry* 2016; **6:** e821.

133. Rodd ZA, Bertsch BA, Strother WN, Le-Niculescu H, Balaraman Y, Hayden E *et al.* Candidate genes, pathways and mechanisms for alcoholism: an expanded convergent functional genomics approach. *Pharmacogenomics J* 2007; **7**(4)**:** 222-256.

134. Chang LC, Jamain S, Lin CW, Rujescu D, Tseng GC, Sibille E. A conserved BDNF, glutamate- and GABA-enriched gene module related to human depression identified by coexpression meta-analysis and DNA variant genome-wide association studies. *PloS one* 2014; **9**(3)**:** e90980.

135. Flatscher-Bader T, van der Brug M, Hwang JW, Gochee PA, Matsumoto I, Niwa S *et al.* Alcohol-responsive genes in the frontal cortex and nucleus accumbens of human alcoholics. *Journal of neurochemistry* 2005; **93**(2)**:** 359-370.

136. Le-Niculescu H, McFarland MJ, Ogden CA, Balaraman Y, Patel S, Tan J *et al.* Phenomic, convergent functional genomic, and biomarker studies in a stress-reactive genetic animal model of bipolar disorder and co-morbid alcoholism. *Am J Med Genet B Neuropsychiatr Genet* 2008; **147B**(2)**:** 134-166.

137. Major Depressive Disorder Working Group of the Psychiatric GC, Ripke S, Wray NR, Lewis CM, Hamilton SP, Weissman MM *et al.* A mega-analysis of genome-wide association studies for major depressive disorder. *Molecular psychiatry* 2013; **18**(4)**:** 497-511.

138. Scott LJ, Muglia P, Kong XQ, Guan W, Flickinger M, Upmanyu R *et al.* Genome-wide association and meta-analysis of bipolar disorder in individuals of European ancestry. *Proc Natl Acad Sci U S A* 2009; **106**(18)**:** 7501-7506.

139. McMahon FJ, Akula N, Schulze TG, Muglia P, Tozzi F, Detera-Wadleigh SD *et al.* Meta-analysis of genome-wide association data identifies a risk locus for major mood disorders on 3p21.1. *Nat Genet* 2010; **42**(2)**:** 128-131.

140. Glatt SJ, Cohen OS, Faraone SV, Tsuang MT. Dysfunctional gene splicing as a potential contributor to neuropsychiatric disorders. *Am J Med Genet B Neuropsychiatr Genet* 2011; **156B**(4)**:** 382-392.

141. Kondo K, Ikeda M, Kajio Y, Saito T, Iwayama Y, Aleksic B *et al.* Genetic variants on 3q21 and in the Sp8 transcription factor gene (SP8) as susceptibility loci for psychotic disorders: a genetic association study. *PLoS One* 2013; **8**(8)**:** e70964.

142. Vassos E, Steinberg S, Cichon S, Breen G, Sigurdsson E, Andreassen OA *et al.* Replication study and meta-analysis in European samples supports association of the 3p21.1 locus with bipolar disorder. *Biol Psychiatry* 2012; **72**(8)**:** 645-650.

143. Chen DT, Jiang X, Akula N, Shugart YY, Wendland JR, Steele CJ *et al.* Genome-wide association study meta-analysis of European and Asian-ancestry samples identifies three novel loci associated with bipolar disorder. *Mol Psychiatry* 2013; **18**(2)**:** 195-205.

144. Goes FS, Hamshere ML, Seifuddin F, Pirooznia M, Belmonte-Mahon P, Breuer R *et al.* Genome-wide association of mood-incongruent psychotic bipolar disorder. *Transl Psychiatry* 2012; **2:** e180.

145. Schizophrenia Working Group of the Psychiatric Genomics C. Biological insights from 108 schizophrenia-associated genetic loci. *Nature* 2014; **511**(7510)**:** 421-427.

146. Erikson GA, Bodian DL, Rueda M, Molparia B, Scott ER, Scott-Van Zeeland AA *et al.* Whole-Genome Sequencing of a Healthy Aging Cohort. *Cell* 2016; **165**(4)**:** 1002-1011.

147. Kurian SM, Le-Niculescu H, Patel SD, Bertram D, Davis J, Dike C *et al.* Identification of blood biomarkers for psychosis using convergent functional genomics. *Molecular psychiatry* 2011; **16**(1)**:** 37-58.

148. Le-Niculescu H, Kurian SM, Yehyawi N, Dike C, Patel SD, Edenberg HJ *et al.* Identifying blood biomarkers for mood disorders using convergent functional genomics. *Molecular psychiatry* 2009; **14**(2)**:** 156-174.

149. Niculescu AB, 3rd, Segal DS, Kuczenski R, Barrett T, Hauger RL, Kelsoe JR. Identifying a series of candidate genes for mania and psychosis: a convergent functional genomics approach. *Physiol Genomics* 2000; **4**(1)**:** 83-91.

150. Liu J, Lewohl JM, Harris RA, Iyer VR, Dodd PR, Randall PK *et al.* Patterns of gene expression in the frontal cortex discriminate alcoholic from nonalcoholic individuals. *Neuropsychopharmacology* 2006; **31**(7)**:** 1574-1582.

151. Mayfield RD, Lewohl JM, Dodd PR, Herlihy A, Liu J, Harris RA. Patterns of gene expression are altered in the frontal and motor cortices of human alcoholics. *Journal of neurochemistry* 2002; **81**(4)**:** 802-813.

152. McClintick JN, Xuei X, Tischfield JA, Goate A, Foroud T, Wetherill L *et al.* Stress-response pathways are altered in the hippocampus of chronic alcoholics. *Alcohol* 2013; **47**(7)**:** 505-515.

153. Forero DA, Guio-Vega GP, Gonzalez-Giraldo Y. A comprehensive regional analysis of genome-wide expression profiles for major depressive disorder. *Journal of affective disorders* 2017; **218:** 86-92.

154. Kuo B, Bhasin M, Jacquart J, Scult MA, Slipp L, Riklin EI *et al.* Genomic and clinical effects associated with a relaxation response mind-body intervention in patients with irritable bowel syndrome and inflammatory bowel disease. *PloS one* 2015; **10**(4)**:** e0123861.

155. Lo CL, Lossie AC, Liang T, Liu Y, Xuei X, Lumeng L *et al.* High Resolution Genomic Scans Reveal Genetic Architecture Controlling Alcohol Preference in Bidirectionally Selected Rat Model. *PLoS genetics* 2016; **12**(8)**:** e1006178.

156. Le-Niculescu H, Balaraman Y, Patel S, Tan J, Sidhu K, Jerome RE *et al.* Towards understanding the schizophrenia code: an expanded convergent functional genomics approach. *Am J Med Genet B Neuropsychiatr Genet* 2007; **144B**(2)**:** 129-158.

157. O'Dushlaine C, Kenny E, Heron E, Donohoe G, Gill M, Morris D *et al.* Molecular pathways involved in neuronal cell adhesion and membrane scaffolding contribute to schizophrenia and bipolar disorder susceptibility. *Molecular psychiatry* 2011; **16**(3)**:** 286-292.

158. Yu H, Bi W, Liu C, Zhao Y, Zhang D, Yue W. A hypothesis-driven pathway analysis reveals myelin-related pathways that contribute to the risk of schizophrenia and bipolar disorder. *Progress in neuro-psychopharmacology & biological psychiatry* 2014; **51:** 140-145.

159. Sokolowski M, Wasserman J, Wasserman D. Polygenic associations of neurodevelopmental genes in suicide attempt. *Molecular psychiatry* 2016; **21**(10)**:** 1381-1390.

160. Konradi C, Eaton M, MacDonald ML, Walsh J, Benes FM, Heckers S. Molecular evidence for mitochondrial dysfunction in bipolar disorder. *Arch Gen Psychiatry* 2004; **61**(3)**:** 300-308.

161. Roussos P, Guennewig B, Kaczorowski DC, Barry G, Brennand KJ. Activity-Dependent Changes in Gene Expression in Schizophrenia Human-Induced Pluripotent Stem Cell Neurons. *JAMA psychiatry* 2016; **73**(11)**:** 1180-1188.

162. Focking M, Lopez LM, English JA, Dicker P, Wolff A, Brindley E *et al.* Proteomic and genomic evidence implicates the postsynaptic density in schizophrenia. *Molecular psychiatry* 2015; **20**(4)**:** 424-432.

163. Gottschalk MG, Wesseling H, Guest PC, Bahn S. Proteomic enrichment analysis of psychotic and affective disorders reveals common signatures in presynaptic glutamatergic signaling and energy metabolism. *Int J Neuropsychopharmacol* 2014; **18**(2).

164. Vawter MP, Ferran E, Galke B, Cooper K, Bunney WE, Byerley W. Microarray screening of lymphocyte gene expression differences in a multiplex schizophrenia pedigree. *Schizophr Res* 2004; **67**(1)**:** 41-52.

165. Hoyo-Becerra C, Huebener A, Trippler M, Lutterbeck M, Liu ZJ, Truebner K *et al.* Concomitant interferon alpha stimulation and TLR3 activation induces neuronal expression of depression-related genes that are elevated in the brain of suicidal persons. *PloS one* 2013; **8**(12)**:** e83149.

166. Chang X, Liu Y, Hahn CG, Gur RE, Sleiman PMA, Hakonarson H. RNA-seq analysis of amygdala tissue reveals characteristic expression profiles in schizophrenia. *Translational psychiatry* 2017; **7**(8)**:** e1203.

167. Saetre P, Emilsson L, Axelsson E, Kreuger J, Lindholm E, Jazin E. Inflammation-related genes up-regulated in schizophrenia brains. *BMC psychiatry* 2007; **7:** 46.

168. Breen MS, Maihofer AX, Glatt SJ, Tylee DS, Chandler SD, Tsuang MT *et al.* Gene networks specific for innate immunity define post-traumatic stress disorder. *Molecular psychiatry* 2015; **20**(12)**:** 1538-1545.

169. Xuei X, Flury-Wetherill L, Almasy L, Bierut L, Tischfield J, Schuckit M *et al.* Association analysis of genes encoding the nociceptin receptor (OPRL1) and its endogenous ligand (PNOC) with alcohol or illicit drug dependence. *Addiction biology* 2008; **13**(1)**:** 80-87.

170. Fillman SG, Sinclair D, Fung SJ, Webster MJ, Shannon Weickert C. Markers of inflammation and stress distinguish subsets of individuals with schizophrenia and bipolar disorder. *Translational psychiatry* 2014; **4:** e365.

171. Gaiteri C, Guilloux JP, Lewis DA, Sibille E. Altered gene synchrony suggests a combined hormone-mediated dysregulated state in major depression. *PloS one* 2010; **5**(4)**:** e9970.

172. Brennand KJ, Simone A, Jou J, Gelboin-Burkhart C, Tran N, Sangar S *et al.* Modelling schizophrenia using human induced pluripotent stem cells. *Nature* 2011; **473**(7346)**:** 221-225.

173. Der-Avakian A, D'Souza MS, Potter DN, Chartoff EH, Carlezon WA, Jr., Pizzagalli DA *et al.* Social defeat disrupts reward learning and potentiates striatal nociceptin/orphanin FQ mRNA in rats. *Psychopharmacology* 2017; **234**(9-10)**:** 1603-1614.

174. Li JZ, Bunney BG, Meng F, Hagenauer MH, Walsh DM, Vawter MP *et al.* Circadian patterns of gene expression in the human brain and disruption in major depressive disorder. *Proc Natl Acad Sci U S A* 2013; **110**(24)**:** 9950-9955.

175. Tochigi M, Iwamoto K, Bundo M, Sasaki T, Kato N, Kato T. Gene expression profiling of major depression and suicide in the prefrontal cortex of postmortem brains. *Neuroscience research* 2008; **60**(2)**:** 184-191.

176. Treadwell JA, Singh SM. Microarray analysis of mouse brain gene expression following acute ethanol treatment. *Neurochem Res* 2004; **29**(2)**:** 357-369.

177. Sokolov BP, Jiang L, Trivedi NS, Aston C. Transcription profiling reveals mitochondrial, ubiquitin and signaling systems abnormalities in postmortem brains from subjects with a history of alcohol abuse or dependence. *Journal of neuroscience research* 2003; **72**(6)**:** 756-767.

178. Licznerski P, Duric V, Banasr M, Alavian KN, Ota KT, Kang HJ *et al.* Decreased SGK1 Expression and Function Contributes to Behavioral Deficits Induced by Traumatic Stress. *PLoS biology* 2015; **13**(10)**:** e1002282.

179. Mehta D, Klengel T, Conneely KN, Smith AK, Altmann A, Pace TW *et al.* Childhood maltreatment is associated with distinct genomic and epigenetic profiles in posttraumatic stress disorder. *Proceedings of the National Academy of Sciences of the United States of America* 2013; **110**(20)**:** 8302-8307.

180. Karayiorgou M, Altemus M, Galke BL, Goldman D, Murphy DL, Ott J *et al.* Genotype determining low catechol-O-methyltransferase activity as a risk factor for obsessive-compulsive disorder. *Proceedings of the National Academy of Sciences of the United States of America* 1997; **94**(9)**:** 4572-4575.

181. Karayiorgou M, Sobin C, Blundell ML, Galke BL, Malinova L, Goldberg P *et al.* Family-based association studies support a sexually dimorphic effect of COMT and MAOA on genetic susceptibility to obsessive-compulsive disorder. *Biological psychiatry* 1999; **45**(9)**:** 1178-1189.

182. Rotondo A, Mazzanti C, Dell'Osso L, Rucci P, Sullivan P, Bouanani S *et al.* Catechol o-methyltransferase, serotonin transporter, and tryptophan hydroxylase gene polymorphisms in bipolar disorder patients with and without comorbid panic disorder. *Am J Psychiatry* 2002; **159**(1)**:** 23-29.

183. Denys D, Van Nieuwerburgh F, Deforce D, Westenberg H. Association between the dopamine D2 receptor TaqI A2 allele and low activity COMT allele with obsessive-compulsive disorder in males. *European neuropsychopharmacology : the journal of the European College of Neuropsychopharmacology* 2006; **16**(6)**:** 446-450.

184. Katerberg H, Lochner C, Cath DC, de Jonge P, Bochdanovits Z, Moolman-Smook JC *et al.* The role of the brain-derived neurotrophic factor (BDNF) val66met variant in the phenotypic expression of obsessive-compulsive disorder (OCD). *American journal of medical genetics Part B, Neuropsychiatric genetics : the official publication of the International Society of Psychiatric Genetics* 2009; **150B**(8)**:** 1050-1062.

185. Pooley EC, Fineberg N, Harrison PJ. The met(158) allele of catechol-O-methyltransferase (COMT) is associated with obsessive-compulsive disorder in men: case-control study and meta-analysis. *Molecular psychiatry* 2007; **12**(6)**:** 556-561.

186. Gothelf D, Michaelovsky E, Frisch A, Zohar AH, Presburger G, Burg M *et al.* Association of the low-activity COMT 158Met allele with ADHD and OCD in subjects with velocardiofacial syndrome. *The international journal of neuropsychopharmacology* 2007; **10**(3)**:** 301-308.

187. Mynett-Johnson LA, Murphy VE, Claffey E, Shields DC, McKeon P. Preliminary evidence of an association between bipolar disorder in females and the catechol-O-methyltransferase gene. *Psychiatric genetics* 1998; **8**(4)**:** 221-225.

188. Burdick KE, Funke B, Goldberg JF, Bates JA, Jaeger J, Kucherlapati R *et al.* COMT genotype increases risk for bipolar I disorder and influences neurocognitive performance. *Bipolar disorders* 2007; **9**(4)**:** 370-376.

189. Soronen P, Silander K, Antila M, Palo OM, Tuulio-Henriksson A, Kieseppa T *et al.* Association of a nonsynonymous variant of DAOA with visuospatial ability in a bipolar family sample. *Biol Psychiatry* 2008; **64**(5)**:** 438-442.

190. Shifman S, Bronstein M, Sternfeld M, Pisante A, Weizman A, Reznik I *et al.* COMT: a common susceptibility gene in bipolar disorder and schizophrenia. *American journal of medical genetics Part B, Neuropsychiatric genetics : the official publication of the International Society of Psychiatric Genetics* 2004; **128B**(1)**:** 61-64.

191. Hukic DS, Frisen L, Backlund L, Lavebratt C, Landen M, Traskman-Bendz L *et al.* Cognitive manic symptoms in bipolar disorder associated with polymorphisms in the DAOA and COMT genes. *PloS one* 2013; **8**(7)**:** e67450.

192. Clelland CL, Drouet V, Rilett KC, Smeed JA, Nadrich RH, Rajparia A *et al.* Evidence that COMT genotype and proline interact on negative-symptom outcomes in schizophrenia and bipolar disorder. *Transl Psychiatry* 2016; **6**(9)**:** e891.

193. Hamilton SP, Slager SL, Heiman GA, Deng Z, Haghighi F, Klein DF *et al.* Evidence for a susceptibility locus for panic disorder near the catechol-O-methyltransferase gene on chromosome 22. *Biological psychiatry* 2002; **51**(7)**:** 591-601.

194. Domschke K, Freitag CM, Kuhlenbaumer G, Schirmacher A, Sand P, Nyhuis P *et al.* Association of the functional V158M catechol-O-methyl-transferase polymorphism with panic disorder in women. *The international journal of neuropsychopharmacology* 2004; **7**(2)**:** 183-188.

195. Rothe C, Koszycki D, Bradwejn J, King N, Deluca V, Tharmalingam S *et al.* Association of the Val158Met catechol O-methyltransferase genetic polymorphism with panic disorder. *Neuropsychopharmacology : official publication of the American College of Neuropsychopharmacology* 2006; **31**(10)**:** 2237-2242.

196. Stein DJ, Newman TK, Savitz J, Ramesar R. Warriors versus worriers: the role of COMT gene variants. *CNS spectrums* 2006; **11**(10)**:** 745-748.

197. Domschke K, Deckert J, O'Donovan M C, Glatt SJ. Meta-analysis of COMT val158met in panic disorder: ethnic heterogeneity and gender specificity. *American journal of medical genetics Part B, Neuropsychiatric genetics : the official publication of the International Society of Psychiatric Genetics* 2007; **144B**(5)**:** 667-673.

198. Wray NR, James MR, Dumenil T, Handoko HY, Lind PA, Montgomery GW *et al.* Association study of candidate variants of COMT with neuroticism, anxiety and depression. *American journal of medical genetics Part B, Neuropsychiatric genetics : the official publication of the International Society of Psychiatric Genetics* 2008; **147B**(7)**:** 1314-1318.

199. Hettema JM, An SS, Bukszar J, van den Oord EJ, Neale MC, Kendler KS *et al.* Catechol-O-methyltransferase contributes to genetic susceptibility shared among anxiety spectrum phenotypes. *Biological psychiatry* 2008; **64**(4)**:** 302-310.

200. Kang EH, Song YJ, Kim KJ, Shim HB, Park JE, Yu BH. Sympathetic nervous function and the effect of the catechol-O-methyltransferase Val(158)Met polymorphism in patients with panic disorder. *Journal of affective disorders* 2010; **123**(1-3)**:** 337-340.

201. Howe AS, Buttenschon HN, Bani-Fatemi A, Maron E, Otowa T, Erhardt A *et al.* Candidate genes in panic disorder: meta-analyses of 23 common variants in major anxiogenic pathways. *Molecular psychiatry* 2016; **21**(5)**:** 665-679.

202. Shifman S, Bronstein M, Sternfeld M, Pisante-Shalom A, Lev-Lehman E, Weizman A *et al.* A highly significant association between a COMT haplotype and schizophrenia. *American journal of human genetics* 2002; **71**(6)**:** 1296-1302.

203. Sun X, Jia Y, Zhang X, Xu Q, Shen Y, Li Y. Multi-locus association study of schizophrenia susceptibility genes with a posterior probability method. *Science in China Series C, Life sciences* 2005; **48**(3)**:** 263-269.

204. Talkowski ME, Kirov G, Bamne M, Georgieva L, Torres G, Mansour H *et al.* A network of dopaminergic gene variations implicated as risk factors for schizophrenia. *Human molecular genetics* 2008; **17**(5)**:** 747-758.

205. Hoenicka J, Garrido E, Martinez I, Ponce G, Aragues M, Rodriguez-Jimenez R *et al.* Gender-specific COMT Val158Met polymorphism association in Spanish schizophrenic patients. *American journal of medical genetics Part B, Neuropsychiatric genetics : the official publication of the International Society of Psychiatric Genetics* 2010; **153B**(1)**:** 79-85.

206. Glessner JT, Reilly MP, Kim CE, Takahashi N, Albano A, Hou C *et al.* Strong synaptic transmission impact by copy number variations in schizophrenia. *Proc Natl Acad Sci U S A* 2010; **107**(23)**:** 10584-10589.

207. Wright GE, Niehaus DJ, van der Merwe L, Koen L, Korkie LJ, Kinnear CJ *et al.* Association of MB-COMT polymorphisms with schizophrenia-susceptibility and symptom severity in an African cohort. *Progress in neuro-psychopharmacology & biological psychiatry* 2012; **39**(1)**:** 163-169.

208. Chen J, Calhoun VD, Perrone-Bizzozero NI, Pearlson GD, Sui J, Du Y *et al.* A pilot study on commonality and specificity of copy number variants in schizophrenia and bipolar disorder. *Translational psychiatry* 2016; **6**(5)**:** e824.

209. Funke B, Malhotra AK, Finn CT, Plocik AM, Lake SL, Lencz T *et al.* COMT genetic variation confers risk for psychotic and affective disorders: a case control study. *Behavioral and brain functions : BBF* 2005; **1:** 19.

210. Rujescu D, Giegling I, Gietl A, Hartmann AM, Moller HJ. A functional single nucleotide polymorphism (V158M) in the COMT gene is associated with aggressive personality traits. *Biological psychiatry* 2003; **54**(1)**:** 34-39.

211. Perroud N, Jaussent I, Guillaume S, Bellivier F, Baud P, Jollant F *et al.* COMT but not serotonin-related genes modulates the influence of childhood abuse on anger traits. *Genes, brain, and behavior* 2010; **9**(2)**:** 193-202.

212. Vassos E, Collier DA, Fazel S. Systematic meta-analyses and field synopsis of genetic association studies of violence and aggression. *Mol Psychiatry* 2014; **19**(4)**:** 471-477.

213. Ono H, Shirakawa O, Nushida H, Ueno Y, Maeda K. Association between catechol-O-methyltransferase functional polymorphism and male suicide completers. *Neuropsychopharmacology : official publication of the American College of Neuropsychopharmacology* 2004; **29**(7)**:** 1374-1377.

214. Jia CX, Zhao ZT, Hu MH, Gao LJ, Wang XT. [A paired case-control study on related factors to attempted suicide]. *Zhonghua liu xing bing xue za zhi = Zhonghua liuxingbingxue zazhi* 2005; **26**(5)**:** 339-343.

215. Baud P, Courtet P, Perroud N, Jollant F, Buresi C, Malafosse A. Catechol-O-methyltransferase polymorphism (COMT) in suicide attempters: a possible gender effect on anger traits. *American journal of medical genetics Part B, Neuropsychiatric genetics : the official publication of the International Society of Psychiatric Genetics* 2007; **144B**(8)**:** 1042-1047.

216. Lee HY, Kim YK. Gender effect of catechol-O-methyltransferase Val158Met polymorphism on suicidal behavior. *Neuropsychobiology* 2011; **63**(3)**:** 177-182.

217. Pivac N, Pregelj P, Nikolac M, Zupanc T, Nedic G, Muck Seler D *et al.* The association between catechol-O-methyl-transferase Val108/158Met polymorphism and suicide. *Genes, brain, and behavior* 2011; **10**(5)**:** 565-569.

218. Nielsen LM, Christrup LL, Sato H, Drewes AM, Olesen AE. Genetic Influences of OPRM1, OPRD1 and COMT on Morphine Analgesia in a Multi-Modal, Multi-Tissue Human Experimental Pain Model. *Basic & clinical pharmacology & toxicology* 2017.

219. Li T, Chen CK, Hu X, Ball D, Lin SK, Chen W *et al.* Association analysis of the DRD4 and COMT genes in methamphetamine abuse. *American journal of medical genetics Part B, Neuropsychiatric genetics : the official publication of the International Society of Psychiatric Genetics* 2004; **129B**(1)**:** 120-124.

220. Weickert TW, Goldberg TE, Mishara A, Apud JA, Kolachana BS, Egan MF *et al.* Catechol-O-methyltransferase val108/158met genotype predicts working memory response to antipsychotic medications. *Biological psychiatry* 2004; **56**(9)**:** 677-682.

221. Domschke K, Zavorotnyy M, Diemer J, Nitsche S, Hohoff C, Baune BT *et al.* COMT val158met influence on electroconvulsive therapy response in major depression. *American journal of medical genetics Part B, Neuropsychiatric genetics : the official publication of the International Society of Psychiatric Genetics* 2010; **153B**(1)**:** 286-290.

222. Schosser A, Calati R, Serretti A, Massat I, Kocabas NA, Papageorgiou K *et al.* The impact of COMT gene polymorphisms on suicidality in treatment resistant major depressive disorder--a European multicenter study. *European neuropsychopharmacology : the journal of the European College of Neuropsychopharmacology* 2012; **22**(4)**:** 259-266.

223. Wong ML, Dong C, Andreev V, Arcos-Burgos M, Licinio J. Prediction of susceptibility to major depression by a model of interactions of multiple functional genetic variants and environmental factors. *Molecular psychiatry* 2012; **17**(6)**:** 624-633.

224. Kolassa IT, Kolassa S, Ertl V, Papassotiropoulos A, De Quervain DJ. The risk of posttraumatic stress disorder after trauma depends on traumatic load and the catechol-o-methyltransferase Val(158)Met polymorphism. *Biological psychiatry* 2010; **67**(4)**:** 304-308.

225. Clark R, DeYoung CG, Sponheim SR, Bender TL, Polusny MA, Erbes CR *et al.* Predicting post-traumatic stress disorder in veterans: interaction of traumatic load with COMT gene variation. *Journal of psychiatric research* 2013; **47**(12)**:** 1849-1856.

226. Nedic G, Nikolac M, Sviglin KN, Muck-Seler D, Borovecki F, Pivac N. Association study of a functional catechol-O-methyltransferase (COMT) Val108/158Met polymorphism and suicide attempts in patients with alcohol dependence. *The international journal of neuropsychopharmacology* 2011; **14**(3)**:** 377-388.

227. Beech RD, Qu J, Leffert JJ, Lin A, Hong KA, Hansen J *et al.* Altered expression of cytokine signaling pathway genes in peripheral blood cells of alcohol dependent subjects: preliminary findings. *Alcoholism, clinical and experimental research* 2012; **36**(9)**:** 1487-1496.

228. Grant P, Gabriel F, Kuepper Y, Wielpuetz C, Hennig J. Psychosis-proneness correlates with expression levels of dopaminergic genes. *European psychiatry : the journal of the Association of European Psychiatrists* 2014; **29**(5)**:** 304-306.

229. Melas PA, Rogdaki M, Osby U, Schalling M, Lavebratt C, Ekstrom TJ. Epigenetic aberrations in leukocytes of patients with schizophrenia: association of global DNA methylation with antipsychotic drug treatment and disease onset. *FASEB journal : official publication of the Federation of American Societies for Experimental Biology* 2012; **26**(6)**:** 2712-2718.

230. Huotari M, Garcia-Horsman JA, Karayiorgou M, Gogos JA, Mannisto PT. D-amphetamine responses in catechol-O-methyltransferase (COMT) disrupted mice. *Psychopharmacology* 2004; **172**(1)**:** 1-10.

231. Babovic D, O'Tuathaigh CM, O'Connor AM, O'Sullivan GJ, Tighe O, Croke DT *et al.* Phenotypic characterization of cognition and social behavior in mice with heterozygous versus homozygous deletion of catechol-O-methyltransferase. *Neuroscience* 2008; **155**(4)**:** 1021-1029.

232. Chen JS, Papaleo F, Tian QJ, Glineburg P, Song J, Liu GP *et al.* Development of Knock-in Mutant Mice Carrying Human Catechol-O-Methyltransferase Met Mutation Mimicking the Functional Val158Met Polymorphism. *Biol Psychiat* 2009; **65**(8)**:** 42s-42s.

233. Segall SK, Nackley AG, Diatchenko L, Lariviere WR, Lu X, Marron JS *et al.* Comt1 genotype and expression predicts anxiety and nociceptive sensitivity in inbred strains of mice. *Genes, brain, and behavior* 2010; **9**(8)**:** 933-946.

234. Skinner MK, Anway MD, Savenkova MI, Gore AC, Crews D. Transgenerational epigenetic programming of the brain transcriptome and anxiety behavior. *PloS one* 2008; **3**(11)**:** e3745.

235. Benes FM, Lim B, Subburaju S. Site-specific regulation of cell cycle and DNA repair in post-mitotic GABA cells in schizophrenic versus bipolars. *Proceedings of the National Academy of Sciences of the United States of America* 2009; **106**(28)**:** 11731-11736.

236. Maycox PR, Kelly F, Taylor A, Bates S, Reid J, Logendra R *et al.* Analysis of gene expression in two large schizophrenia cohorts identifies multiple changes associated with nerve terminal function. *Molecular psychiatry* 2009; **14**(12)**:** 1083-1094.

237. Sheng G, Demers M, Subburaju S, Benes FM. Differences in the circuitry-based association of copy numbers and gene expression between the hippocampi of patients with schizophrenia and the hippocampi of patients with bipolar disorder. *Archives of general psychiatry* 2012; **69**(6)**:** 550-561.

238. Arnold SE, Xie SX, Leung YY, Wang LS, Kling MA, Han X *et al.* Plasma biomarkers of depressive symptoms in older adults. *Translational psychiatry* 2012; **2:** e65.

239. Lee BH, Kim YK. Increased plasma VEGF levels in major depressive or manic episodes in patients with mood disorders. *Journal of affective disorders* 2012; **136**(1-2)**:** 181-184.

240. De Rossi P, Harde E, Dupuis JP, Martin L, Chounlamountri N, Bardin M *et al.* A critical role for VEGF and VEGFR2 in NMDA receptor synaptic function and fear-related behavior. *Molecular psychiatry* 2016; **21**(12)**:** 1768-1780.

241. Fromer M, Roussos P, Sieberts SK, Johnson JS, Kavanagh DH, Perumal TM *et al.* Gene expression elucidates functional impact of polygenic risk for schizophrenia. *Nature neuroscience* 2016; **19**(11)**:** 1442-1453.

242. Cittaro D, Lampis V, Luchetti A, Coccurello R, Guffanti A, Felsani A *et al.* Histone Modifications in a Mouse Model of Early Adversities and Panic Disorder: Role for Asic1 and Neurodevelopmental Genes. *Scientific reports* 2016; **6:** 25131.

243. Jazwinski SM, Kim S, Dai J, Li L, Bi X, Jiang JC *et al.* HRAS1 and LASS1 with APOE are associated with human longevity and healthy aging. *Aging cell* 2010; **9**(5)**:** 698-708.

244. Flory JD, Donohue D, Muhie S, Yang R, Miller SA, Hammamieh R *et al.* Gene expression associated with suicide attempts in US veterans. *Translational psychiatry* 2017; **7**(9)**:** e1226.

245. McBride WJ, Kimpel MW, Schultz JA, McClintick JN, Edenberg HJ, Bell RL. Changes in gene expression in regions of the extended amygdala of alcohol-preferring rats after binge-like alcohol drinking. *Alcohol* 2010; **44**(2)**:** 171-183.

246. Su YA, Wu J, Zhang L, Zhang Q, Su DM, He P *et al.* Dysregulated mitochondrial genes and networks with drug targets in postmortem brain of patients with posttraumatic stress disorder (PTSD) revealed by human mitochondria-focused cDNA microarrays. *Int J Biol Sci* 2008; **4**(4)**:** 223-235.

247. Deelen J, Uh HW, Monajemi R, van Heemst D, Thijssen PE, Bohringer S *et al.* Gene set analysis of GWAS data for human longevity highlights the relevance of the insulin/IGF-1 signaling and telomere maintenance pathways. *Age* 2013; **35**(1)**:** 235-249.

248. Law AJ, Wang Y, Sei Y, O'Donnell P, Piantadosi P, Papaleo F *et al.* Neuregulin 1-ErbB4-PI3K signaling in schizophrenia and phosphoinositide 3-kinase-p110delta inhibition as a potential therapeutic strategy. *Proceedings of the National Academy of Sciences of the United States of America* 2012; **109**(30)**:** 12165-12170.

249. Meda SA, Ruano G, Windemuth A, O'Neil K, Berwise C, Dunn SM *et al.* Multivariate analysis reveals genetic associations of the resting default mode network in psychotic bipolar disorder and schizophrenia. *Proceedings of the National Academy of Sciences of the United States of America* 2014; **111**(19)**:** E2066-2075.

250. Malki K, Keers R, Tosto MG, Lourdusamy A, Carboni L, Domenici E *et al.* The endogenous and reactive depression subtypes revisited: integrative animal and human studies implicate multiple distinct molecular mechanisms underlying major depressive disorder. *BMC medicine* 2014; **12:** 73.

251. Zhao Z, Xu J, Chen J, Kim S, Reimers M, Bacanu SA *et al.* Transcriptome sequencing and genome-wide association analyses reveal lysosomal function and actin cytoskeleton remodeling in schizophrenia and bipolar disorder. *Molecular psychiatry* 2015; **20**(5)**:** 563-572.

252. Darby MM, Yolken RH, Sabunciyan S. Consistently altered expression of gene sets in postmortem brains of individuals with major psychiatric disorders. *Translational psychiatry* 2016; **6**(9)**:** e890.

253. Karssen AM, Her S, Li JZ, Patel PD, Meng F, Bunney WE, Jr. *et al.* Stress-induced changes in primate prefrontal profiles of gene expression. *Mol Psychiatry* 2007; **12**(12)**:** 1089-1102.

254. Wu Q, Huang JH. Ectopic expression of Smurf2 and acceleration of age-related intervertebral disc degeneration in a mouse model. *Journal of neurosurgery Spine* 2017; **27**(1)**:** 116-126.

255. Uhl GR, Drgon T, Liu QR, Johnson C, Walther D, Komiyama T *et al.* Genome-wide association for methamphetamine dependence: convergent results from 2 samples. *Arch Gen Psychiatry* 2008; **65**(3)**:** 345-355.

256. Vrijenhoek T, Buizer-Voskamp JE, van der Stelt I, Strengman E, Genetic R, Outcome in Psychosis C *et al.* Recurrent CNVs disrupt three candidate genes in schizophrenia patients. *American journal of human genetics* 2008; **83**(4)**:** 504-510.

257. Wang KS, Liu XF, Aragam N. A genome-wide meta-analysis identifies novel loci associated with schizophrenia and bipolar disorder. *Schizophr Res* 2010; **124**(1-3)**:** 192-199.

258. Glessner JT, Wang K, Cai G, Korvatska O, Kim CE, Wood S *et al.* Autism genome-wide copy number variation reveals ubiquitin and neuronal genes. *Nature* 2009; **459**(7246)**:** 569-573.

259. Bremer A, Giacobini M, Eriksson M, Gustavsson P, Nordin V, Fernell E *et al.* Copy number variation characteristics in subpopulations of patients with autism spectrum disorders. *American journal of medical genetics Part B, Neuropsychiatric genetics : the official publication of the International Society of Psychiatric Genetics* 2011; **156**(2)**:** 115-124.

260. Shao L, Vawter MP. Shared gene expression alterations in schizophrenia and bipolar disorder. *Biological psychiatry* 2008; **64**(2)**:** 89-97.

261. Rautiainen MR, Paunio T, Repo-Tiihonen E, Virkkunen M, Ollila HM, Sulkava S *et al.* Genome-wide association study of antisocial personality disorder. *Translational psychiatry* 2016; **6**(9)**:** e883.

262. Cole SW, Hawkley LC, Arevalo JM, Sung CY, Rose RM, Cacioppo JT. Social regulation of gene expression in human leukocytes. *Genome Biol* 2007; **8**(9)**:** R189.

263. Kuan PF, Waszczuk MA, Kotov R, Marsit CJ, Guffanti G, Gonzalez A *et al.* An epigenome-wide DNA methylation study of PTSD and depression in World Trade Center responders. *Translational psychiatry* 2017; **7**(6)**:** e1158.

264. Surget A, Wang Y, Leman S, Ibarguen-Vargas Y, Edgar N, Griebel G *et al.* Corticolimbic transcriptome changes are state-dependent and region-specific in a rodent model of depression and of antidepressant reversal. *Neuropsychopharmacology* 2009; **34**(6)**:** 1363-1380.

265. Zhao Z, Xu J, Chen J, Kim S, Reimers M, Bacanu SA *et al.* Transcriptome sequencing and genome-wide association analyses reveal lysosomal function and actin cytoskeleton remodeling in schizophrenia and bipolar disorder. *Molecular psychiatry* 2015; **20**(5)**:** 563-572.

266. Stelzhammer V, Alsaif M, Chan MK, Rahmoune H, Steeb H, Guest PC *et al.* Distinct proteomic profiles in post-mortem pituitary glands from bipolar disorder and major depressive disorder patients. *J Psychiatr Res* 2015; **60:** 40-48.

267. Bergen SE, O'Dushlaine CT, Ripke S, Lee PH, Ruderfer DM, Akterin S *et al.* Genome-wide association study in a Swedish population yields support for greater CNV and MHC involvement in schizophrenia compared with bipolar disorder. *Mol Psychiatry* 2012; **17**(9)**:** 880-886.

268. Fernandez-Castillo N, Cabana-Dominguez J, Soriano J, Sanchez-Mora C, Roncero C, Grau-Lopez L *et al.* Transcriptomic and genetic studies identify NFAT5 as a candidate gene for cocaine dependence. *Transl Psychiatry* 2015; **5:** e667.

269. Lewohl JM, Wang L, Miles MF, Zhang L, Dodd PR, Harris RA. Gene expression in human alcoholism: microarray analysis of frontal cortex. *Alcohol Clin Exp Res* 2000; **24**(12)**:** 1873-1882.

270. Canli T, Wen R, Wang X, Mikhailik A, Yu L, Fleischman D *et al.* Differential transcriptome expression in human nucleus accumbens as a function of loneliness. *Molecular psychiatry* 2016.

271. Bell RL, Kimpel MW, McClintick JN, Strother WN, Carr LG, Liang T *et al.* Gene expression changes in the nucleus accumbens of alcohol-preferring rats following chronic ethanol consumption. *Pharmacol Biochem Behav* 2009; **94**(1)**:** 131-147.

272. Scharf JM, Yu D, Mathews CA, Neale BM, Stewart SE, Fagerness JA *et al.* Genome-wide association study of Tourette's syndrome. *Molecular psychiatry* 2013; **18**(6)**:** 721-728.

273. Sommer JU, Schmitt A, Heck M, Schaeffer EL, Fendt M, Zink M *et al.* Differential expression of presynaptic genes in a rat model of postnatal hypoxia: relevance to schizophrenia. *European archives of psychiatry and clinical neuroscience* 2010; **260 Suppl 2:** S81-89.

274. Jung JY, Kohane IS, Wall DP. Identification of autoimmune gene signatures in autism. *Translational psychiatry* 2011; **1:** e63.

275. Cao-Lei L, Massart R, Suderman MJ, Machnes Z, Elgbeili G, Laplante DP *et al.* DNA methylation signatures triggered by prenatal maternal stress exposure to a natural disaster: Project Ice Storm. *PloS one* 2014; **9**(9)**:** e107653.

276. Yehuda R, Cai G, Golier JA, Sarapas C, Galea S, Ising M *et al.* Gene expression patterns associated with posttraumatic stress disorder following exposure to the World Trade Center attacks. *Biol Psychiatry* 2009; **66**(7)**:** 708-711.

277. Hammamieh R, Chakraborty N, Gautam A, Miller SA, Muhie S, Meyerhoff J *et al.* Transcriptomic analysis of the effects of a fish oil enriched diet on murine brains. *PLoS One* 2014; **9**(3)**:** e90425.

278. Le-Niculescu H, Case NJ, Hulvershorn L, Patel SD, Bowker D, Gupta J *et al.* Convergent functional genomic studies of omega-3 fatty acids in stress reactivity, bipolar disorder and alcoholism. *Translational psychiatry* 2011; **1:** e4.

279. Adkins DE, Aberg K, McClay JL, Bukszar J, Zhao Z, Jia P *et al.* Genomewide pharmacogenomic study of metabolic side effects to antipsychotic drugs. *Mol Psychiatry* 2011; **16**(3)**:** 321-332.

280. Youngs RM, Chu MS, Meloni EG, Naydenov A, Carlezon WA, Jr., Konradi C. Lithium administration to preadolescent rats causes long-lasting increases in anxiety-like behavior and has molecular consequences. *J Neurosci* 2006; **26**(22)**:** 6031-6039.

281. Rybakowski JK, Czerski P, Dmitrzak-Weglarz M, Kliwicki S, Leszczynska-Rodziewicz A, Permoda-Osip A *et al.* Clinical and pathogenic aspects of candidate genes for lithium prophylactic efficacy. *Journal of psychopharmacology* 2012; **26**(3)**:** 368-373.

282. Le-Niculescu H, Balaraman Y, Patel SD, Ayalew M, Gupta J, Kuczenski R *et al.* Convergent functional genomics of anxiety disorders: translational identification of genes, biomarkers, pathways and mechanisms. *Translational psychiatry* 2011; **1:** e9.

283. Jakovcevski M, Bharadwaj R, Straubhaar J, Gao G, Gavin DP, Jakovcevski I *et al.* Prefrontal cortical dysfunction after overexpression of histone deacetylase 1. *Biological psychiatry* 2013; **74**(9)**:** 696-705.

284. Takahashi Y, Washiyama K, Kobayashi T, Hayashi S. Gene expression in the brain from fluoxetine-injected mouse using DNA microarray. *Annals of the New York Academy of Sciences* 2006; **1074:** 42-51.

285. Sugawara H, Iwamoto K, Bundo M, Ishiwata M, Ueda J, Kakiuchi C *et al.* Effect of mood stabilizers on gene expression in lymphoblastoid cells. *Journal of neural transmission* 2010; **117**(2)**:** 155-164.

286. Melka MG, Laufer BI, McDonald P, Castellani CA, Rajakumar N, O'Reilly R *et al.* The effects of olanzapine on genome-wide DNA methylation in the hippocampus and cerebellum. *Clinical epigenetics* 2014; **6**(1)**:** 1.

287. Herteleer L, Zwarts L, Hens K, Forero D, Del-Favero J, Callaerts P. Mood stabilizing drugs regulate transcription of immune, neuronal and metabolic pathway genes in Drosophila. *Psychopharmacology* 2016; **233**(9)**:** 1751-1762.

288. Lagus M, Gass N, Saharinen J, Saarela J, Porkka-Heiskanen T, Paunio T. Gene expression patterns in a rodent model for depression. *Eur J Neurosci* 2010; **31**(8)**:** 1465-1473.

289. Ogden CA, Rich ME, Schork NJ, Paulus MP, Geyer MA, Lohr JB *et al.* Candidate genes, pathways and mechanisms for bipolar (manic-depressive) and related disorders: an expanded convergent functional genomics approach. *Mol Psychiatry* 2004; **9**(11)**:** 1007-1029.

290. Feher LZ, Kalman J, Puskas LG, Gyulveszi G, Kitajka K, Penke B *et al.* Impact of haloperidol and risperidone on gene expression profile in the rat cortex. *Neurochemistry international* 2005; **47**(4)**:** 271-280.

291. Rangaraju S, Levey DF, Nho K, Jain N, Andrews KD, Le-Niculescu H *et al.* Mood, stress and longevity: convergence on ANK3. *Molecular psychiatry* 2016; **21**(8)**:** 1037-1049.
